# A decreasing glacier mass balance gradient from the edge of the Upper Tarim Basin to the Karakoram during 2000–2014

**DOI:** 10.1038/s41598-017-07133-8

**Published:** 2017-07-27

**Authors:** Hui Lin, Gang Li, Lan Cuo, Andrew Hooper, Qinghua Ye

**Affiliations:** 1 Institute of Space and Earth Information Science, The Chinese University of Hong Kong, Hong Kong S.A.R., China; 2 Geography and Resource Management, The Chinese University of Hong Kong, Hong Kong S.A.R., China; 3 Shenzhen Research Institute, The Chinese University of Hong Kong, Shenzhen, 518057 China; 4 Institute of Tibetan Plateau Research, Chinese Academy of Sciences, Beijing, 100101 China; 5 CAS Center for Excellence in Tibetan Plateau Earth Sciences, Beijing, 100101 China; 6 Key Laboratory of Tibetan Environment Changes and Land Surface Processes, Institute of Tibetan Plateau Research, Chinese Academy of Sciences, Beijing, 100101 China; 7 COMET, School of Earth and Environment, University of Leeds, Leeds, United Kingdom

## Abstract

In contrast to the glacier mass losses observed at other locations around the world, some glaciers in the High Mountains of Asia appear to have gained mass in recent decades. However, changes in digital elevation models indicate that glaciers in Karakoram and Pamir have gained mass, while recent laser altimetry data indicate mass gain centred on West Kunlun. Here, we obtain results that are essentially consistent with those from altimetry, but with two-dimensional observations and higher resolution. We produced elevation models using radar interferometry applied to bistatic data gathered between 2011 and 2014 and compared them to a model produced from bistatic data collected in 2000. The glaciers in West Kunlun, Eastern Pamir and the northern part of Karakoram experienced a clear mass gain of 0.043 ± 0.078~0.363 ± 0.065 m w.e. yr^−1^. The Karakoram showed a near-stable mass balance in its western part (−0.020 ± 0.064 m w.e. yr^−1^), while the Eastern Karakoram showed mass loss (−0.101 ± 0.058 m w.e. yr^−1^). Significant positive glacier mass balances are noted along the edge of the Upper Tarim Basin and indicate a decreasing gradient from northeast to southwest.

## Introduction

Glaciers and ice sheets throughout the world are experiencing degradation and have contributed approximately 29 ± 13% of the observed sea level increase from 2003 to 2009^1^. The High Mountains of Asia (HMA) contain the world’s largest low-latitude, high-altitude glaciers in the world and are dominated by continental climatic systems, such as the westerlies, the Indian Monsoon and the East Asian Monsoon^2, 3^. Glaciers in the HMA are also the headwater sources of several great rivers, and the HMA is therefore also known as ‘Asian Water Tower’^4^. In contrast to other sub-regions of the HMA, including the Himalayas, Eastern Nyainqentanglha, Spiti Lahaul and Tien Shan, which are experiencing glacier mass loss, the Pamir-Karakoram-Kunlun region includes glaciers that have gained mass, although the details are debated^1, 5–9^. For the Karakoram and the Western Pamir, comparison of digital elevation models (DEMs) from the Shuttle Radar Topographic Mission (SRTM) in 2000 with stereo photogrammetry-based DEMs derived from 2012 SPOT/HRS data suggested a positive mass balance. This was termed the ‘Karakoram anomaly’^5^ and later the ‘Karakoram-Pamir anomaly’^6^. However, a satellite laser altimetry mission, ICESat/GLAS, indicated that a positive mass balance existed in Western Kunlun and Eastern Pamir, but stopped at the edge of the Karakoram, based on the use of a 1 × 1° grid^7, 8^. A similar result was obtained using the same dataset without gridding^1^.

Since 2011, the German Aerospace Centre (DLR) has operated the twin X-band satellites, TerraSAR-X and TanDEM-X (TSX/TDX), in bistatic mode. This mode is similar to the working mode of the SRTM used in 2000, yielding better results regarding glacier height change measurements than ICESat/GLAS laser altimetry or SPOT/HRS photogrammetry^10, 11^. Following previous studies on the ‘Karakoram-Pamir anomaly’, we divide the study site into six sub-regions, including West Kunlun, Extended West Kunlun (including zones A-G), Eastern Karakoram, Western Karakoram, Hindu Kush, and Pamir^5, 6^. We analyse 39 pairs of bistatic SAR images obtained during 2011 and 2014 to derive decadal glacier height changes using the SAR interferometry (InSAR) technique, subtracting SRTM elevations observed in 2000 (Supplementary Fig. S1). The study period used covers a slightly longer time period than previous researches^6, 7^. By presuming a density of 850 ± 60 kg/m^3^ in both the accumulation and ablation zones, we convert the decadal glacier height changes to glacier mass balances^12^.

**Figure 1:**
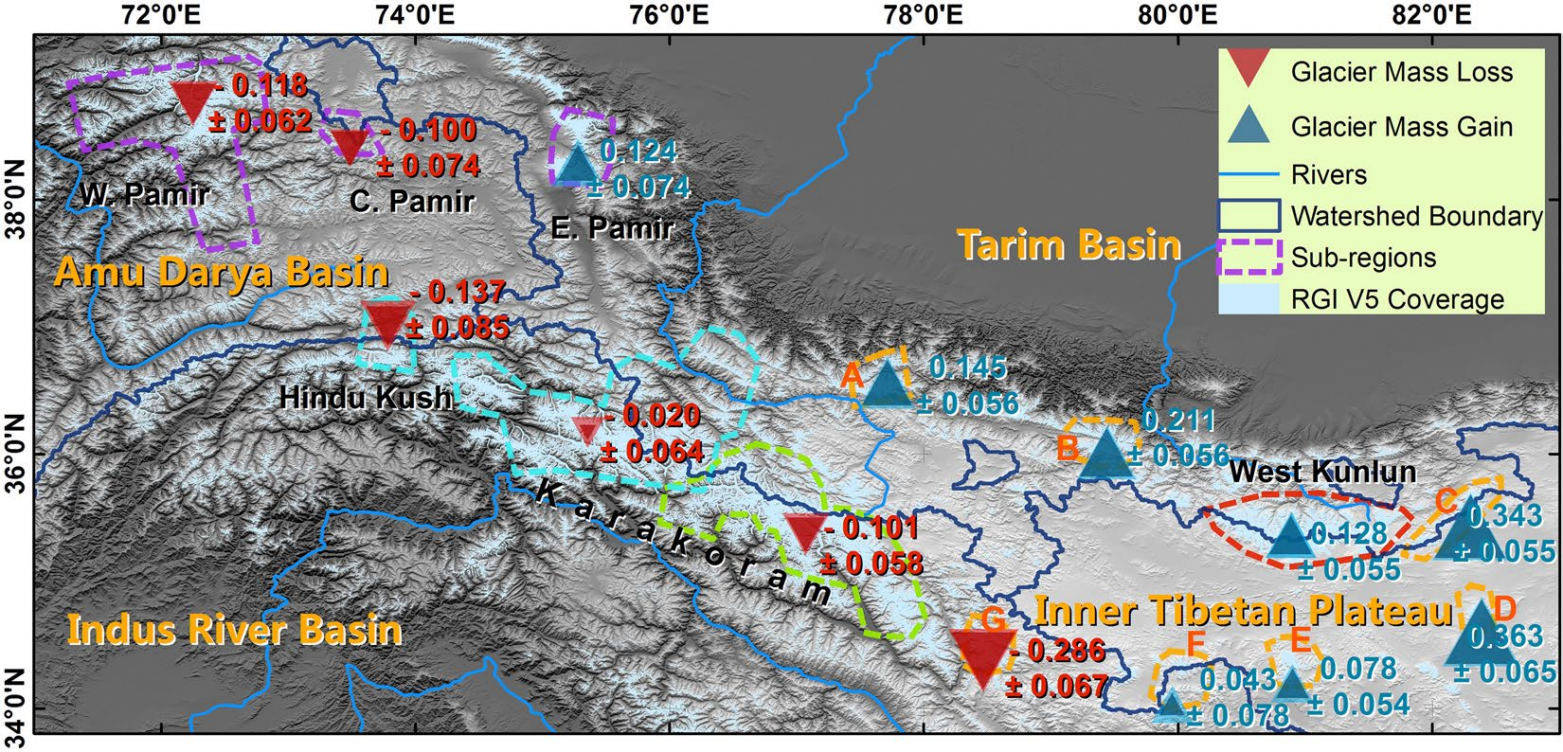
Glacier mass balance in the Karakoram and its surroundings during 2000~2014. The areas of the darker parts of triangles indicate glacier mass balance in terms of m w.e. (water equivalent) yr^−1^, whereas the lighter parts indicate the estimated standard error. The coloured dashed lines indicate the boundaries of each sub-region covered by TSX/TDX images. We subdivided the Extended West Kunlun, which surrounds the West Kunlun, into zones A-G. The Pamir was separated into western, central and eastern parts, which are shown with purple dashed lines. All figures are generated by Gang Li. This figure was generated with ArcGIS 10.2 software (http://www.esri.com/software/arcgis/arcgis-for-desktop).

**Figure 2:**
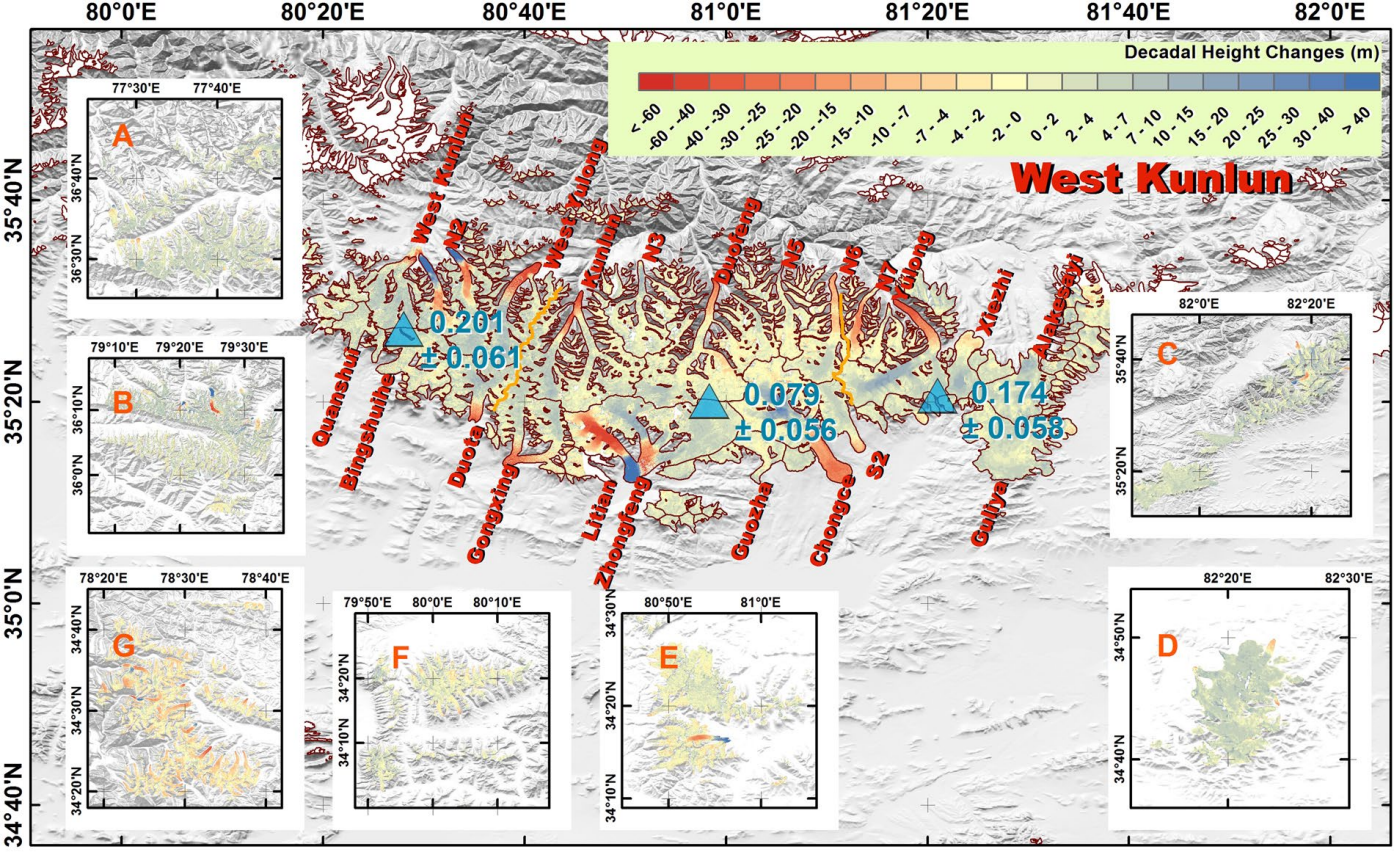
Decadal glacier height changes for the West Kunlun region and its extent during 2000~2014. Locations of A-G are indicated in Fig. 1. Yellow lines separate this region into western, central and eastern parts. Their annual glacier mass balances are shown in units of m w.e. yr^−1^. Glacierized areas without observations are shown in white. This figure was generated with ArcGIS 10.2 software (http://www.esri.com/software/arcgis/arcgis-for-desktop).

The glacier mass balance pattern is heterogeneous at the sub-regional scale (Fig. 1). Only West Kunlun, Extended West Kunlun and Eastern Pamir show glacier mass gain, whereas most of Karakoram, Hindu Kush and Western Pamir lost ice mass. West Kunlun showed a positive mass balance (0.128 ± 0.055 m w.e. yr^−1^), and the surrounding area (A-F) also experienced a mass gain that varied from 0.043 ± 0.078 to 0.363 ± 0.065 m w.e. yr^−1^, with a decreasing gradient from northeast to southwest (Fig. 1, Supplementary Fig. S10). Along the edge of the Upper Tarim Basin in Extended West Kunlun, a more positive mass balance was observed (Fig. 1, zones A, B, C, and D); in almost all elevation bins except those at lowest elevations, height changes were similar and positive (a~d in Supplementary Fig. S19). In the West Kunlun, of the 23 large glaciers that were examined, only three of them showed slight negative mass balances (Fig. 2, Supplementary Table S4). Surging and quiescent glaciers detected by the height change pattern largely agree with results derived from feature tracking^13^. Yulong is the only glacier which is identified as a quiescent glacier in our height change pattern, not identified as such by previous feature tracking^13^. Surging and quiescent glaciers occupy almost half of West Kunlun’s glacierized area, which implies surging is common. Glacier height changes also agree well with results derived from ICESat/GLAS within its footprints^14, 15^. Within the Extended West Kunlun (zone G), which is close to the Eastern Karakoram and Spiti-Lahaul (Fig. 1 and Supplementary Fig. S11), the glacier experienced rapid degradation at a rate of −0.286 ± 0.067 m w.e. yr^−1^. Nevertheless, glacier mass balances in all studied sub-regions or sub-groups were still less negative than the HMA average^8^ of −0.37 ± 0.10 m w.e. yr^−1^.

**Figure 3:**
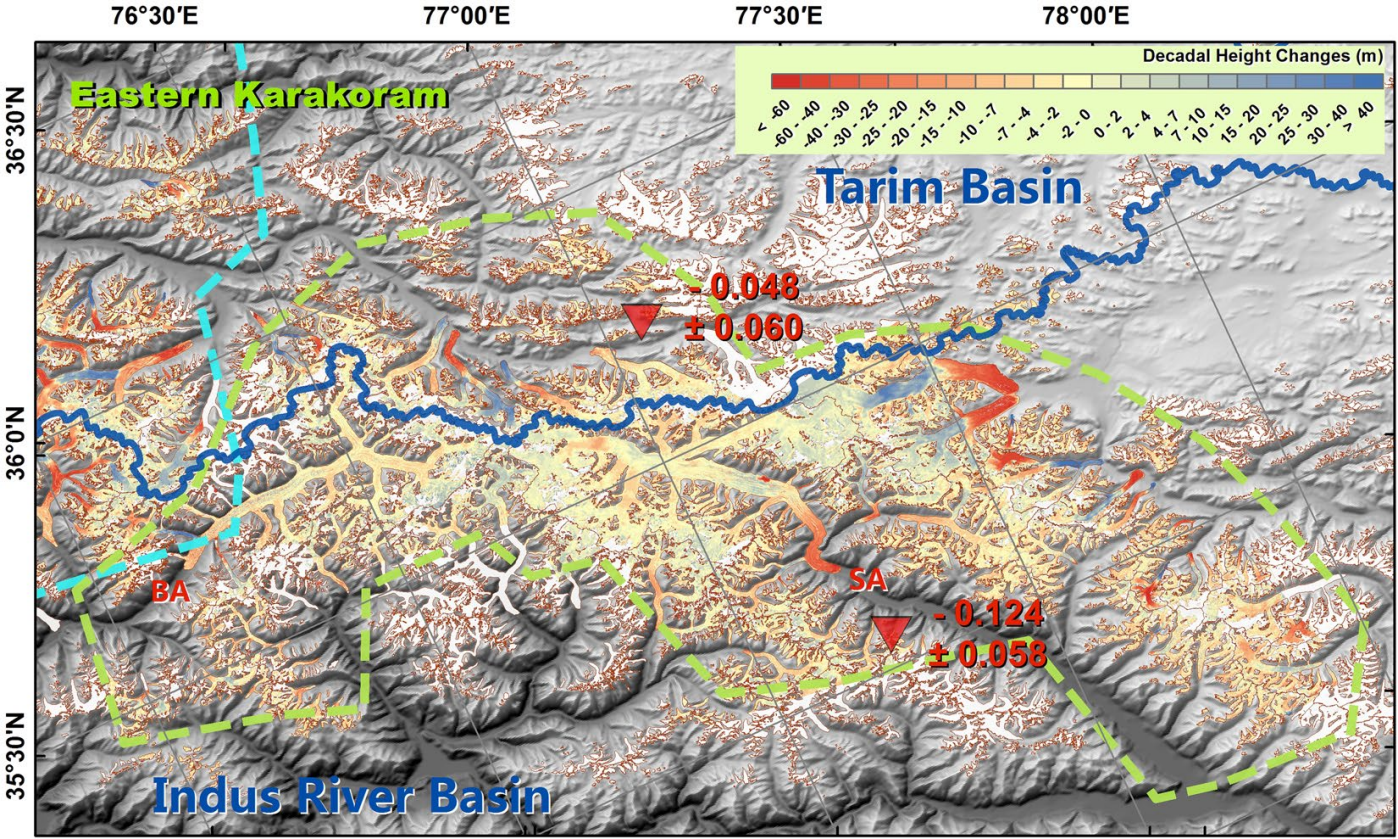
As Fig. 2, but for Eastern Karakoram. The blue line naturally separates this region into the Upper Tarim Basin and the Upper Indus Basin. The boundary of the Eastern Karakoram region is indicated with a green dashed line. SA: Siachen; BA, Baltoro. This figure was generated with ArcGIS 10.2 software (http://www.esri.com/software/arcgis/arcgis-for-desktop).

**Figure 4:**
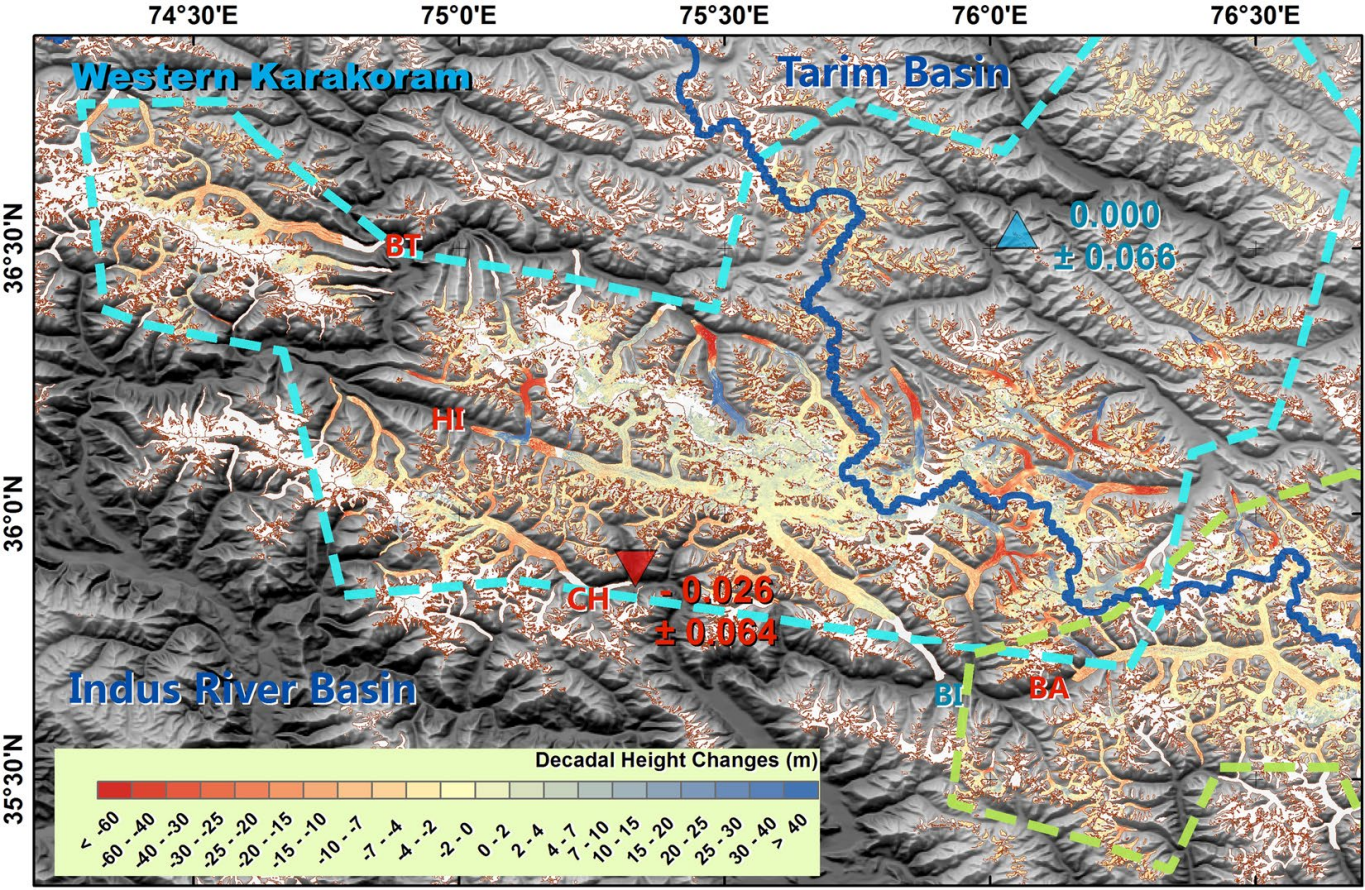
As Fig. 3, but for the Western Karakoram. BI, Biafo; CH, Chogo Lungma; HI, Hispar; BT, Batura. This figure was generated with ArcGIS 10.2 software (http://www.esri.com/software/arcgis/arcgis-for-desktop).

A large number of glaciers in Karakoram surged or experienced quiescent phases after surging, mostly on the northern slope or within the Upper Tarim Basin^16^. Taking these glaciers into account by adding them to the regional mass balance regarding area as weight^6^, the mass balances of glaciers in the Eastern and Western Karakoram regions were −0.101 ± 0.058 and −0.020 ± 0.064 m w.e. yr^−1^, respectively. These results were closer to the ICESat/GLAS-derived results^7, 8^ than the results derived from topographic differencing between SRTM and stereo SPOT/HRS^5, 6^ data, even though our study period was similar to that used in the latter analysis. The latter analysis yielded a positive mass balance in both the Eastern and the Western Karakoram of 0.11 ± 0.14 and 0.09 ± 0.18 m w.e. yr^−1^, respectively^6^. The central part of Karakoram was more stable than the fringing regions to both the west and east (Supplementary Table S3). Most of the mass gain occurred on the northern slope of the Karakoram Mountains. For both the Eastern and Western Karakoram, the glacier mass balance was more negative in the Upper Indus Basin than in the Upper Tarim Basin (Figs 3 and 4; Supplementary Tables S5 and S6). Glacier mass balances were 0.000 ± 0.066 and −0.048 ± 0.060 m w.e. yr^−1^ in the western and eastern parts inside the Upper Tarim region, respectively (Figs 3 and 4). At the far northeastern part of the Karakoram, which is covered by frame 2014–02–08n (Supplementary Fig. S1), close to the edge of the Upper Tarim, a significant positive mass balance was found at 0.114 ± 0.070 m w.e. yr^−1^, with similar rates of height increase in all elevation bins (Supplementary Fig. S29). This also suggests that the anomaly centre was not within the central Karakoram, but at the southeastern edge of the Upper Tarim^7^. Glacier mass balance in the Hindu Kush region has been under debate; our results show a negative total mass balance of −0.134 ± 0.085 m w.e. yr^−1^.

**Figure 5:**
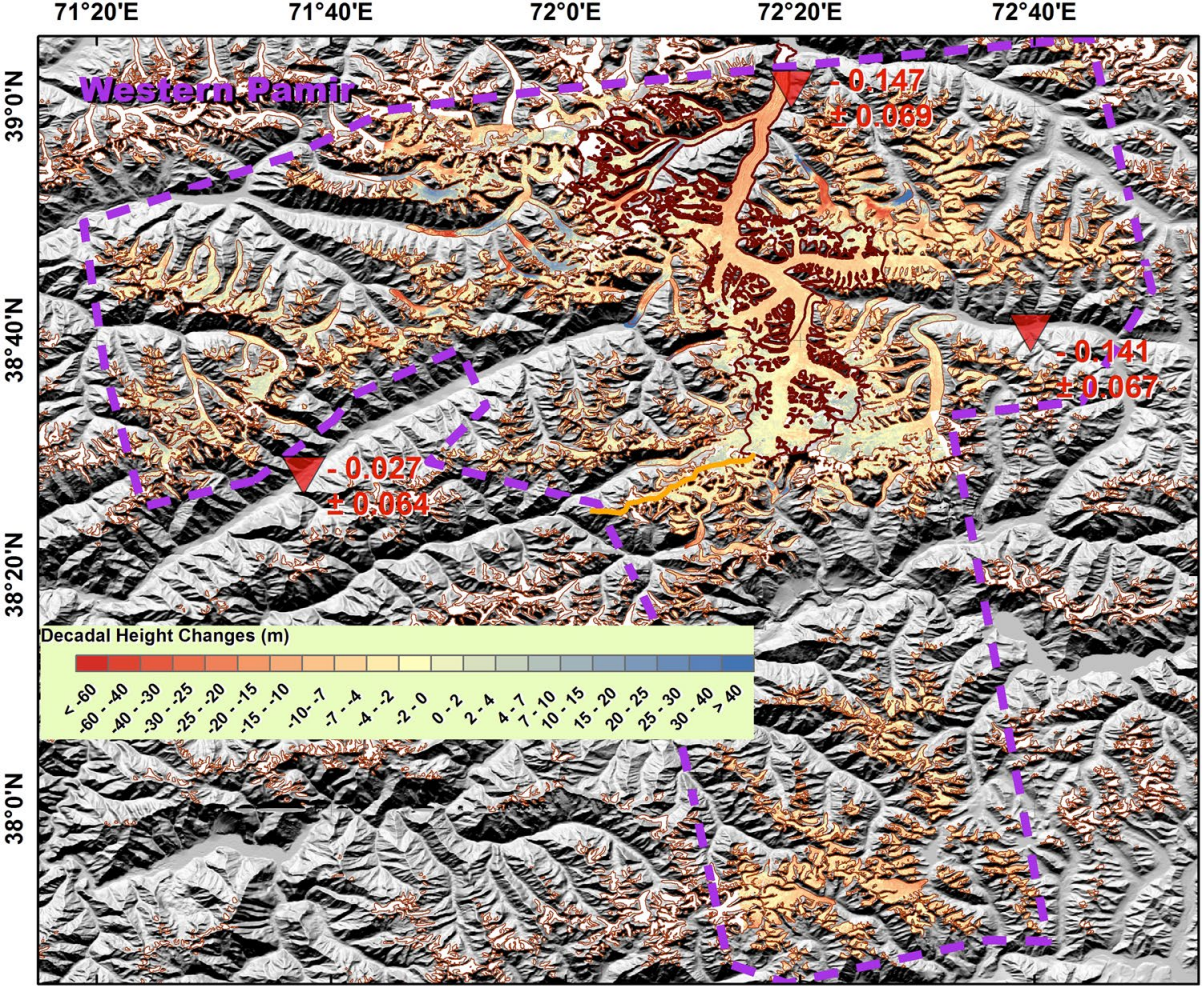
As Fig. 2, but for the Western Pamir region. The Fedchenko Glacier is indicated by the bold boundary. The yellow line separates the rest of the glaciers into two sub-regions. The purple dashed line represents the TSX/TDX coverage. This figure was generated with ArcGIS 10.2 software (http://www.esri.com/software/arcgis/arcgis-for-desktop).

**Figure 6:**
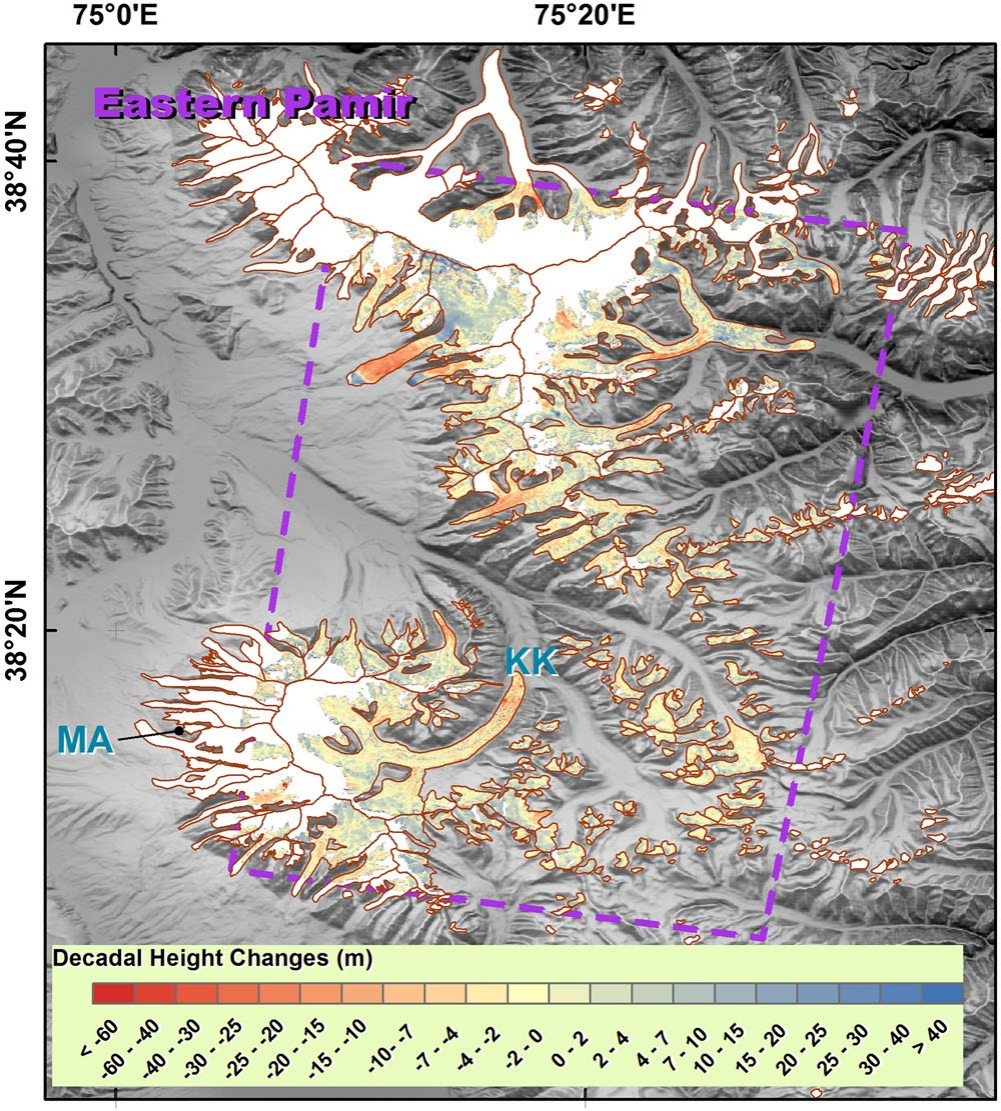
As Fig. 2, but for the Eastern Pamir region. The purple dashed line represents the TSX/TDX coverage. KK, Kekesayi glacier; MA, Muztag Ata glacier. This figure was generated with ArcGIS 10.2 software (http://www.esri.com/software/arcgis/arcgis-for-desktop).

The Pamir region is divided into western, central and eastern sub-regions. Our findings suggest negative mass balances in both the Western and the Central Pamir regions, and a positive mass balance in Eastern Pamir, with values of −0.118 ± 0.032, −0.100 ± 0.087, and 0.124 ± 0.086 m w.e. yr^−1^, respectively. Gardelle *et al*.^6^ suggested that there was a positive mass balance in Western Pamir of 0.14 ± 0.14 m w.e. yr^−1^, and that the Fedchenko Glacier, which is the largest glacier in this sub-region (Fig. 5 and Supplementary Fig. S14), was nearly stable. However, Gardner *et al*.^1^ and Kääb *et al*.^7^ identified negative sub-regional mass balances of −0.13 ± 0.11 and −0.48 ± 0.14 m w.e. yr^−1^ via satellite laser altimetry. Their major discrepancy lies with glaciers west to Fedchenko. Gardner *et al*.’s results^1^ present positive changes for a lot number of footprints, while Kääb *et al*.’s results^7^ are negative there. Our results suggest Fedchenko Glacier experienced a negative mass balance of −0.147 ± 0.069 m w.e. yr^−1^, as well as significant thinning in the ablation zone (Supplementary Fig. S37). Surging and quiescent glaciers are also common in Western Pamir, especially west of the Fedchenko Glacier (Fig. 5). This region, where Gardner *et al*.^1^ and Kääb *et al*.^7^’s discrepancy lies, presents an almost stable mass balance of −0.027 ± 0.064 m w.e. yr^−1^. Previous research using optical satellite identification^17^ shows stable or advancing glaciers in Eastern Pamir. *In-situ* observations within our study period in Eastern Pamir show positive average height changes for the Muztag Ata Glacier^3^. Despite the positive mass balance in both the accumulation and ablation zones, glacier thinning was significant in the very lowest region of the Kekesayi Glacier (Fig. 6 and Supplementary Fig. S41), which is the largest glacier in the Eastern Pamir. This result is similar to the finding of Holzer *et al*., who used photogrammetry^18^.

**Figure 7:**
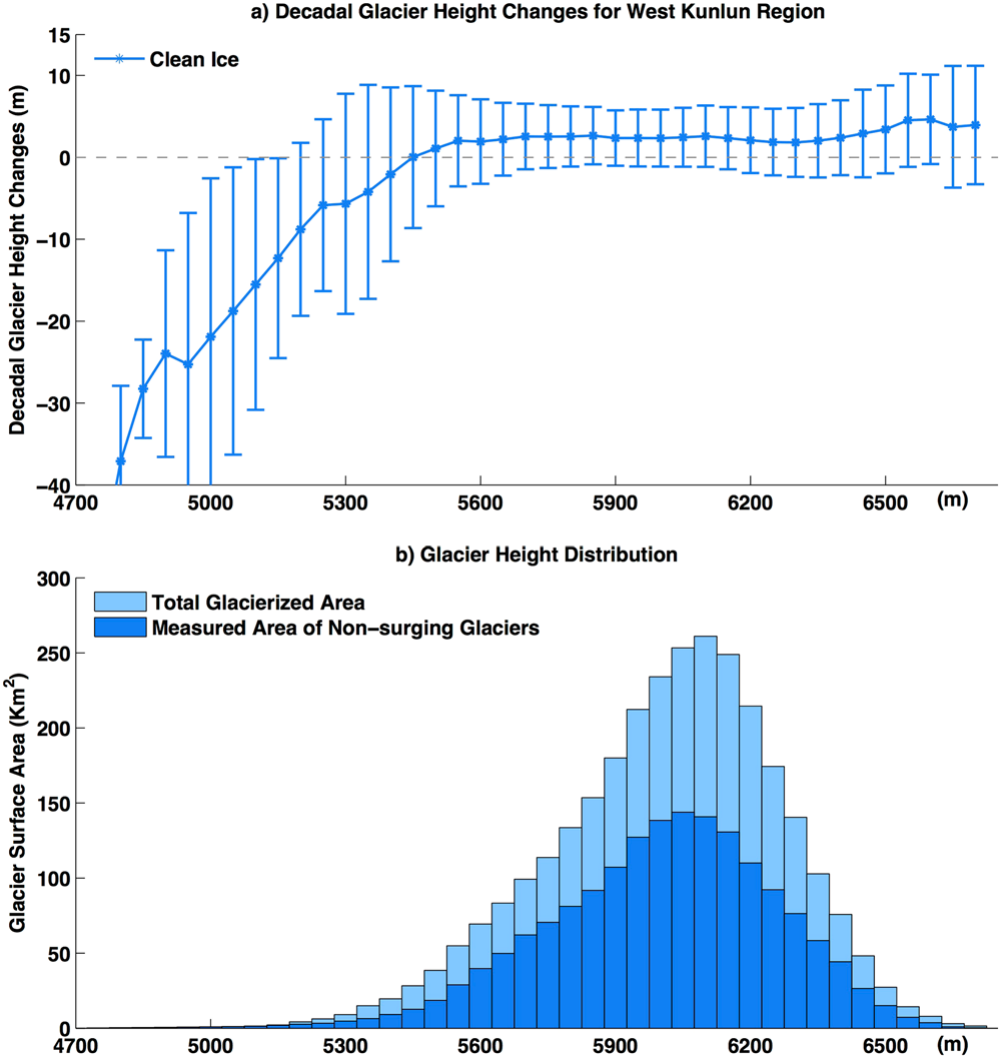
Glacier height changes in different elevation layers in the Western Kunlun. (**a**) Changes in glacier height for non-surging glaciers in each elevation bin. Error bars only indicate standard deviation in height changes in each elevation bin. (**b**) Glacier height distributions for both total glacierized area and the measured area of non-surging glaciers.

Our derived results suggest that the anomalous region is centred on the southern and western edges of the Upper Tarim basin, rather than the Karakoram region. This heterogeneous pattern of glacier mass balance is more similar to the previous 1° × 1° gridded results^7^ derived from ICESat/GLAS satellite laser altimetry than to the results of topographic differencing, despite the small differences in study periods and coverage investigated. This implies that the anomaly indicates strengthening of the westerlies^3^ and increased moisture in the Tarim region^19^. The increase in lake elevation south of the West Kunlun region during almost the same study period also suggests that increasing precipitation could be responsible for the observed glacier mass gain^20, 21^. Large glaciers in the Eastern Pamir and the West Kunlun regions, such as the Kekesayi and the Duofeng Glaciers (Figs 2 and 6), suffered from obvious thinning of up to metres or tens of metres in one decade in their lowest sections. Their rates of height changes in the accumulation zones were identical, indicating that they were not surging glaciers experiencing a quiescent phase. Feature tracking to the Duofeng glacier also confirms this by deriving flow rates^13^. For the West Kunlun region, after removing glaciers that were surging or experiencing a quiescent phase after surging, depending on their flow rates^13^ and height change patterns, the glacier height changes show homogeneously increasing rates in every elevation bin above 5450 m, whereas areas below 5400 m reflect thinning (Fig. 7).

**Figure 8:**
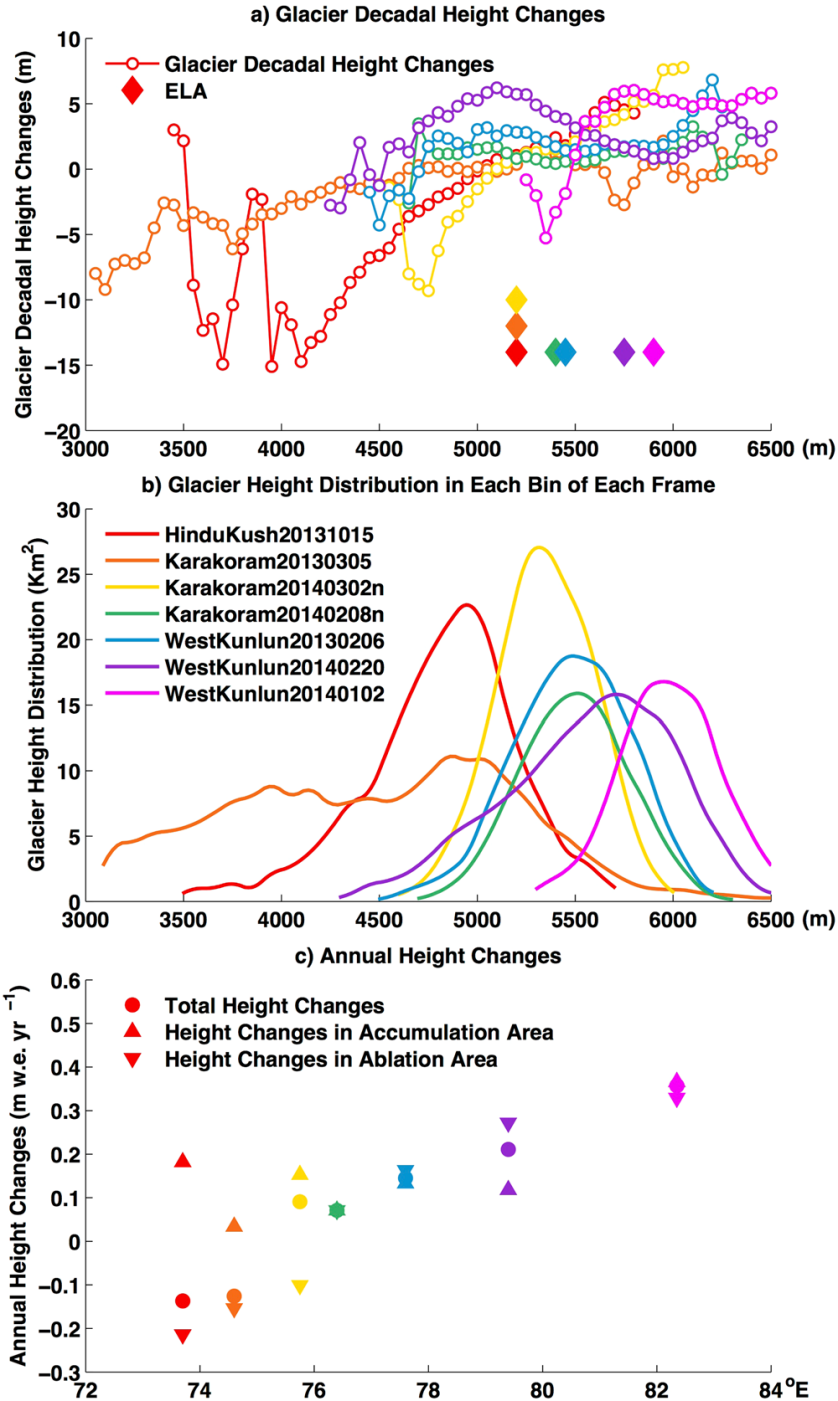
Glacier height changes in different elevation layers along 36.5 N°. (**a**) Glacier height changes in each elevation bin for seven pairs of bistatic images along 36.5 N° (shown in different colours) from the Hindu Kush to the eastern extent of the West Kunlun. The specific position of the coverage refers to Figure S1 in the supplementary. (**b**) Glacier height distribution for seven pairs of image coverages. Different colours indicate different frames. (**c**) Annual glacier height changes for the total region, accumulation area and ablation area in seven pairs of bistatic images along 36.5 N°. The horizontal axis indicates the longitude at the centre of each image.

GCMs simulations do not show a strong mass balance anomaly along the edge of the Upper Tarim region; instead, they suggest that the major anomaly region occurs along 36.5 N° in Western Karakoram^22^. In contrast, seven TSX/TDX images in our study region, covering the Hindu Kush region to the eastern extent of West Kunlun, show that the mass balance increases from west to the east (Fig. 8, Supplementary Figs S1, S19a–c, S23, S27, S28, S29). This is similar to the finding using ICESat/GLAS observations along 36 N°^7^. The northernmost part of Western Karakoram (coverage 2014-03-02n and 2014-02-08n; Supplementary Fig. S1) still presents a positive glacier mass balance. The drainage divide between the Indus Basin and the Tarim Basin seems to represent the limit of this mass balance anomaly. A high proportion of annual streamflow comes from glacier and snow melt in the Upper Indus River, the Upper Tarim River and the Upper Amu Darya River, as the dry desert in the lowland does not produce significant runoff ^23–25^. Climate change-induced snow and glacier changes could therefore be detrimental and may lead to potential conflict in the long run, due to shifts in the seasonal distribution of flow and annual yield changes^26–28^, as the population is projected to grow in central Asia^25, 29^. Some recent studies have already reported that enhanced glacier and snow melt has contributed to an increase in streamflow in the Upper Indus River in recent decades^30–33^.

## Methods

### Bistatic SAR Interferometry

We utilized 39 pairs of X-band SAR images (Supplementary Fig. S1) and used SRTM data from Feb 2000 to detect glacier height changes using bistatic SAR interferometry (InSAR). In the case of bistatic InSAR, because the two images in a single pair are obtained at the same point in time, only the topographic residual phase exists in the differential interferogram. After unwrapping using a minimum cost flow method^34^, we transformed the topographic residual phase directly into height changes^10^. To tie the DEMs to the same reference frame, we assumed no height changes occurred in the off-glacier region in the whole image. Additionally, the off-glacier region was also employed to estimate and remove a bilinear ramp due to orbital errors. The RGI V5.0 dataset was used to provide the boundaries of the glaciers; manual corrections according to Landsat images were performed for several surging and stagnant flow glaciers’ terminus when calculating volume changes and mass balance^35^. The normalized differential snow index (NDSI) of cloud-free end-of-summer Landsat images was applied to identify clean ice glaciers using a threshold of 0.4. Foreshortening, layover, and shadowed regions in the SAR data were de-correlated, and the voids in the SRTM were masked out. Due to the complex topography, a lower proportion of pixels in the higher elevation section can be measured effectively. Therefore, for each elevation bin of 50 m, we calculated the mean height change and calculated the normalized averaged rates of glacier height change for the region of interest.

### Penetration depth and seasonal effect estimation

Microwaves can penetrate snow, firn and ice to a depth that depends on the density, water content and microwave frequency used^36^. Because X-band SRTM is not available everywhere, due to its narrow swath widths, we used C-band SRTM for height change estimation. For each sub-region, we applied C- and X-band SRTM to estimate and remove the penetration depth difference on glaciers individually in each 50-m elevation bin (Supplementary Figs S2–S4). The datum difference between C- and X-band SRTM was estimated and removed by aligning the off-glacier region^37^. The clean ice and the debris-covered ice were treated separately (Supplementary Figs S3 and S4). The estimated average penetration depth differences in the West Kunlun, Karakoram and Pamir regions are 2.84 ± 0.13 m, 2.41 ± 0.17 m, and 1.88 ± 0.29 m, respectively. To avoid the effects of seasonal snow on the penetration depth estimates, we mainly adopted TSX/TDX images obtained in Jan, Feb and Mar. Four pairs of images obtained during different months, with a region of overlap in West Kunlun, were employed to estimate the seasonal effect (Supplementary Figs S5 and S6). We adopted a height change correction of −0.28 m, −0.21 m, −0.14 m, −0.07 m, 0.07 m and 0.14 m for October, November, December, January, March and April, respectively (Supplementary Fig. S1 and Table S1).

### Error estimation

The glacier height change estimates include bias and random error. The former includes differences in the penetration depths associated with the C- and X-bands, the off-glacier region datum and seasonal variations. We performed an error propagation similar to that used in the study of Gardelle *et al*.^6^; autocorrelation distances of 2000 m and 500 m were chosen for the differencing operations between the two SRTM bands and the SRTM and TSX/TDX DEMs, respectively. An error of 0.15 m/month was introduced for the seasonal snow effect estimation. The random error depends on the number of effective measurements at each glacier and on the standard deviation of the elevation differences within the off-glacier region, where no height changes were presumed.

## Electronic supplementary material

Supplementary Information
