# Supplementary material for: A decreasing glacier mass balance gradient from the edge of the Upper Tarim Basin to the Karakoram during 2000–2014
Source: Sci Rep. 2017 Jul 27;7:6712. doi: 10.1038/s41598-017-07133-8 (PMC5532235; doi:10.1038/s41598-017-07133-8)
Supplement: Supplementary file 1 — Supplementary Information [file 41598_2017_7133_MOESM1_ESM.doc]

**Supplementary information:**

**A decreasing glacier mass balance gradient from the edge of the Upper Tarim Basin to the Karakoram during 2000-2014**

**Hui Lin^1, 2, 3^, Gang Li^1*^, Lan Cuo^4, 5, 6^, Andrew Hooper^7^, Qinghua Ye^4^**

**1, Institute of Space and Earth Information Science, The Chinese University of Hong Kong, Hong Kong SAR, China**

**2, Geography and Resource Management, The Chinese University of Hong Kong, Hong Kong S.A.R., China**

**3, Shenzhen Research Institute, The Chinese University of Hong Kong, Shenzhen 518057, China**

**4，Institute of Tibetan Plateau Research, The Chinese Academy of Sciences, Beijing 100101, China**

**5, CAS Center for Excellence in Tibetan Plateau Earth Sciences, Beijing 100101, China**

**6, Key Laboratory of Tibetan Environment Changes and Land Surface Processes Institute of Tibetan Plateau Research, CAS, Beijing 100101, China**

**7，COMET, School of Earth and Environment, University of Leeds, Leeds, United Kingdom**

*** Correspondence to: ligang@link.cuhk.edu.hk**

**1. Study Sites**

Our study sites are located in the Karakoram-Pamir region and its surrounding areas, where the glacier mass balance is still under debate^1-5^. Despite having no distinct boundary, we separated our study site into six sub-regions, including West Kunlun (Red), West Kunlun’s surroundings (Orange), Eastern Karakoram (Green), Western Karakoram (Cyan), Hindu Kush (Cyan), and Pamir (Purple) (Figure S1). The study site spans 71 - 83 °E and 34 - 39 °N. West Kunlun and its surroundings are located in the Upper Tarim Basin and the Inner Tibetan Plateau endorheic region, whereas the Karakoram and the Hindu Kush are mainly located in the Upper Indus and northeastern section of the Tarim basin. The Western and the Central Pamir region are located in the Amu Darya Basin, whereas the Eastern Pamir is located within the Upper Tarim basin. The image coverage for different study sites is marked with different colours.


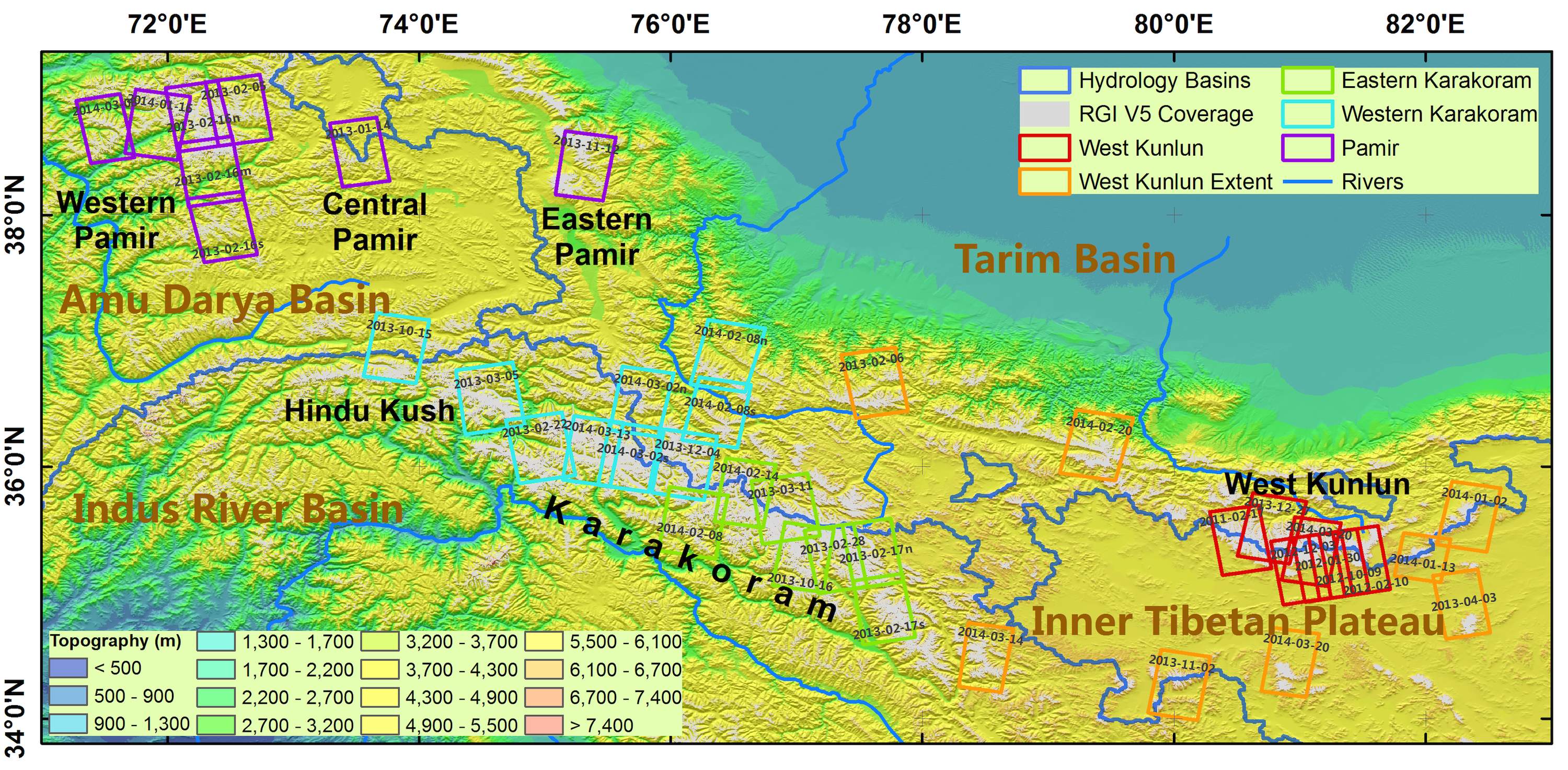


Supplementary Figure S1. Study sites and bistatic SAR image coverage. The dates at which the bistatic SAR images were obtained are labelled on the image coverage boxes. The Hindu Kush coverage is labelled with the same colour as the Western Karakoram group. Randolph glacier inventory V5.0 shows in grey^6^. This figure was generated with ArcGIS 10.2 software (<http://www.esri.com/software/arcgis/arcgis-for-desktop>).

**2. Data and methods**

**2.1 DEMs and SAR data**

To derive decadal height changes, we applied C- (5.6 cm) and X-band (3.1 cm) Shuttle Radar Topography Mission (SRTM) and X-band bistatic TerraSAR-X/TanDEM-X (TSX/TDX) in CoSSC (Coregistered Single look Slant range Complex) format^7, 8^. SRTM DEMs were formed from data acquired by dual-frequency SAR equipment on board the Shuttle Endeavour in 2000 Feb^8^. For different purposes, we used the void-filled SRTM V4 (http://srtm.csi.cgiar.org/), non-void filled SRTM (http://earthexplorer.usgs.gov/) and X-band SRTM DEMs (http://eoweb.dlr.de:8080/free_SRTM_X-band_data.html). The first was regarded as glacier height in 2000 and also as the reference DEM for InSAR processing. We used the non-void filled SRTM to mask out regions where the SRTM V4 was not truly contributed to by SRTM observations, but was filled by other sources, such as ASTER DEM. The resolution of these two DEM datasets is 3-arc (90 m) and they are formed from C-band (5.6 cm) data. We employed the X-band SRTM in 1-arc resolution, provided by the German Aerospace Centre (DLR), for evaluating and removing the penetration depth difference with respect to C-band SRTM, on snow, firn and ice^9^.

Thirty-nine pairs of X-band bistatic TSX/TDX data points in CoSSC format were obtained from DLR. The frame sizes of these images were approximately 30 * 50 km in the ground range and azimuth directions, respectively. Single look images resolution was ~2.5 m. The location and coverage of these images can be found in Figure S1, and more detailed information regarding these images can be found in Table S1. To avoid seasonal variation induced by melting and snow coverage, we mainly chose images that were taken in February, or adjacent months. Bistatic image pairs with an effective perpendicular baseline above 100 m and less than 200 m were preferred, considering both the height ambiguity and coherence of SAR interferometry. The effective perpendicular baseline is half of the real perpendicular baseline because the topographic phase is half in bistatic mode compared to monostatic mode^10^.

Supplementary Table S1. Basic information of the bistatic TSX/TDX images in CoSSC format used in this research. WK: West Kunlun, WKE: West Kunlun Extent, EKK: Eastern Karakoram, WKK: Western Karakoram, HK: Hindu Kush, PMR: Pamir, including the western, central and eastern groups.

| Region | Date | Orbit | A/D | Effective Perpendicular  Baseline (m) | Height  Ambiguity (m) | Master Satellite |
| --- | --- | --- | --- | --- | --- | --- |
| WK | 2011-02-17 | 22 | A | 197 | -40.5 | TSX |
| WK | 2012-01-30 | 113 | A | 84 | -68.1 | TSX |
| WK | 2012-02-10 | 113 | A | 84 | 73.7 | TDX |
| WK | 2012-10-09 | 113 | A | 191 | 30.6 | TDX |
| WK | 2012-12-03 | 113 | A | 172 | -31.3 | TSX |
| WK | 2013-12-27 | 166 | D | 103 | -48.4 | TDX |
| WK | 2014-03-20 | 90 | D | 160 | 48.0 | TDX |
|  |  |  |  |  |  |  |
| WKE | 2013-02-06 | 98 | A | 127 | 56.9 | TDX |
| WKE | 2014-02-20 | 166 | D | 133 | 54.7 | TSX |
| WKE | 2014-01-02 | 90 | D | 106 | 55.6 | TSX |
| WKE | 2014-01-13 | 90 | D | 115 | -55.1 | TDX |
| WKE | 2013-04-03 | 113 | A | 193 | 37.5 | TDX |
| WKE | 2014-03-20 | 90 | D | 160 | 47.9 | TSX |
| WKE | 2013-11-02 | 166 | D | 91 | 62.5 | TSX |
| WKE | 2014-03-14 | 166 | D | 149 | 54.5 | TSX |
|  |  |  |  |  |  |  |
| EKK | 2013-02-17s | 98 | A | 120 | 57.0 | TDX |
| EKK | 2013-02-17n | 98 | A | 120 | 56.9 | TDX |
| EKK | 2013-02-28 | 98 | A | 115 | -55.6 | TSX |
| EKK | 2013-10-16 | 75 | D | 96 | -75.8 | TDX |
| EKK | 2013-03-11 | 98 | A | 112 | -52.9 | TSX |
| EKK | 2014-02-14 | 75 | D | 125 | -65.1 | TDX |
| EKK | 2014-02-08 | 151 | D | 134 | -40.6 | TDX |
|  |  |  |  |  |  |  |
| WKK | 2014-02-08n | 151 | D | 133 | -40.1 | TDX |
| WKK | 2014-02-08s | 151 | D | 133 | -40.7 | TDX |
| WKK | 2013-12-04 | 151 | D | 76 | -75.3 | TDX |
| WKK | 2014-03-02n | 151 | D | 143 | -44.3 | TDX |
| WKK | 2014-03-02s | 151 | D | 143 | -44.3 | TDX |
| WKK | 2014-03-13 | 151 | D | 155 | 43.8 | TSX |
| WKK | 2013-02-22 | 7 | A | 117 | -54.4 | TSX |
| WKK | 2013-03-05 | 7 | A | 110 | -53.7 | TSX |
|  |  |  |  |  |  |  |
| HK | 2013-10-15 | 60 | D | 96 | -64.1 | TDX |
|  |  |  |  |  |  |  |
| PMR | 2014-03-08 | 83 | A | 55 | 95.3 | TDX |
| PMR | 2014-01-16 | 136 | D | 117 | -54.3 | TDX |
| PMR | 2013-02-16n | 83 | A | 122 | 52.2 | TDX |
| PMR | 2013-02-16m | 83 | A | 122 | 52.3 | TDX |
| PMR | 2013-02-16s | 83 | A | 122 | 52.4 | TDX |
| PMR | 2013-02-05 | 83 | A | 128 | -53.3 | TSX |
| PMR | 2013-01-14 | 83 | A | 146 | 55.5 | TDX |
| PMR | 2013-11-12 | 151 | D | 93 | 81.6 | TSX |

**2.2 Bistatic Differential SAR Interferometry**

SAR Interferometry (InSAR) was employed to derive glacier height changes using the reference DEM (SRTM V4) and bistatic TSX/TDX images. The InSAR method is usually employed to derive ground deformation from earthquakes and landslides because the topographic residual phase is often much smaller than the deformation phase; therefore, the topographic residual phase is often presumed to be zero when the deformation is sufficiently high^10^. When detecting slight deformation, such as when performing multi-temporal SAR Interferometry, the topographic residual phase should be accurately estimated and be removed^11^. In bistatic mode InSAR, after estimating the flat-earth and topographic phase using the referencing DEM (SRTM in this study), only the topographic residual phase and possible orbital ramp remain. We estimated the orbital ramp by applying the FFT method to the D-InSAR interferogram and re-estimated the orbit. Then, after removing the orbital ramp and phase unwrapping, we directly transferred the topographic residual phase to topographic height changes. To solve the resolution difference between the reference DEM SRTM (~90 m) and bistatic TSX/TDX (~2.5 m), we applied 4 × 6 multi-look processing when forming an interferogram with TSX/TDX images and performed 8 × 8 oversampling when simulating the topographic residual phase and co-registering the simulated and real SAR images.

Misalignment between two DEMs can result in a false ‘hill-shade’ phenomenon in DEM differencing processing^8, 12^. To estimate and remove the ‘hill-shade’ effect, we accurately co-registered the simulated and real SAR images in the geocoding step to refine the look-up-table between the SAR and geographic coordinates. The Normalized Cross Correlation (NCC) method in the frequency domain was applied for co-registering with search windows at 1024 by 1024 pixels. The RMSE was approximately 0.2 pixels (multi-look image) in SAR coordinates (~2.25 m).

We presumed no deformation or height change occurred at the off-glacier region during the study period. Usually, due to orbital error of TSX/TDX satellites, different height systems between TSX/TDX orbital information (refer to WGS84 ellipsoid) and reference DEM (refer to WGS84 geoid), systematic spatial ramp shows in the height change maps. Although TSX/TDX orbital information is accurate, at full frame scale, still it needs to be evaluated and be removed. In this study, almost all TSX/TDX full frames present systematic ramp less than one fringe, which make height change map correction less than corresponding height ambiguity (Table S1). We applied the RGI V5.0 dataset to separate the glacier region from the off-glacier regions^6^. Several glaciers can be easily identified as surge type on height changing maps; we therefore manually changed their fronts. For the off-glacier region, we applied formula 1 to fit its height differences and then subtract the trend from the whole frame:


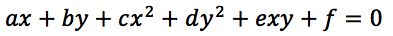
 (1)

where x and y are geographic coordinates in longitude and latitude, and a to f are constants estimated from the off-glacier data.

**2.3 Penetration depth evaluation**

The penetration depth difference between the C- and X-band SAR was estimated by comparing the C- and X-band SRTM. The X-band SRTM suffered from a narrow swathwidth and failed to cover the whole region, which, in addition due to the complex topography at the Karakoram and the Pamir, leads to de-correlation in InSAR processing for generating the SRTM; therefore, we only selected three regions to estimate the penetration depth differences, including West Kunlun, the Karakoram (Near the Hispar Glacier) and Western Pamir. Images of West Kunlun and its extent apply the penetration information of West Kunlun, whereas those of Karakoram and the Hindu Kush region apply the penetration information of Karakoram and those of the Pamir region apply the penetration information of Western Pamir. The penetration depth difference varies when snow/firn/ice density or water content changes; debris-covered regions also demonstrate different penetration depths. We therefore analysed Landsat images (table S2) obtained at the end of summer to separate clean-ice and debris-covered glaciers. The normalized difference snow index (NDSI (TM2-TM5)/(TM2+TM5)) with a threshold of 0.4 was chosen for this task^13^. We analysed the penetration depth in each 50 m elevation bin and plotted the depths with the glacier height distribution (Figure S2-S4). The error bar indicates the standard deviation of the penetration depth difference in each elevation bin. Many gross errors were found in the X-band SRTM because of the geometry distortion of the SAR observation and unwrapping errors. We removed this region after comparing C- and X-band SRTM by presuming that the difference between C- and X-band SRTMs followed a normal distribution. SRTM V3 non-void filled at 1 arc resolution was employed in this step. We also horizontally shift the X-band SRTM according to co-registration of C- and X-band SRTMs in the aim of mitigating false hill-shade phenomenon. At the Karakoram site X-band SRTM shift 0.2 pixels to east, and for Pamir it shifts 0.125 pixels to south and 0.125 pixels to west. For each sub-region, we use the off-glacier region to evaluate datum difference between X- and C- band SRTM by presuming penetration depth difference at off-glacier region is zero. For the West Kunlun region, only clean ice part were analysed since almost no debris were found. For the Karakoram and the Pamir regions, both the clean ice part and the debris-covered part were analysed. Estimated average penetration depth differences in West Kunlun, Karakoram and Pamir are 2.84 ± 0.13 m, 2.41 ± 0.17 m, and 1.88 ± 0.29 m, respectively. Kääb et al.^3^ derived penetration depth of 2.4 ± 0.4 m at Hindu Kush, and 2.4 ± 0.3 m at Karakoram. Gardelle et al.^2^ derived penetration depth of 3.4 m at Karakoram and 1.8 m at Pamir.

The results of this section are the penetration depth differences in each 50 m elevation bin in three regions including West Kunlun, Karakoram, and Pamir. Due to the fact that X-band SRTM only covers a fraction of study site, it is necessary to apply penetration depth difference results to adjacent areas. However, if these two areas are not close enough, the elevation distributions of glacierized area are different. For instance, glacier height distribution is ~400 m lower in West Kunlun A (20130206, Figure S1) than in SRTM-X coverage at West Kunlun (Figure S2). We shift the output in elevations when applying them for penetration depth difference correction in some regions. They are, - 400 m for West Kunlun Extent A and B, - 200 m for Hindu Kush, and - 300 m for Western Pamir. This means that the penetration depth difference in the elevation bin of 4400 and 4450 m estimated in the West Kunlun region is applied to the elevation bin of 4000 and 4050 m in West Kunlun Extent A and B. The average correct elevations are 2.38 m for West Kunlun; 2.22 m, 2.60 m, 2.46 m, 2.05 m, 2.45 m, 2.94 m, 1.91 m for West Kulun Extent A-G; 3.19 m and 2.46 m for Eastern and Western Karakoram; 2.29 m for Hindu Kush; 2.00 m, 1.90 m, and 2.28 m for Western, Central and Eastern Pamir.

Supplementary Table S2. Basic information of Landsat images applied for identifying clean-ice glaciers.

| Satellite & Payload | Path | Row | Observation Date | Sub-region |
| --- | --- | --- | --- | --- |
| Landsat5 TM | 146 | 035 &036 | 1997-08-21 | WKE |
| Landsat5 TM | 147 | 035 & 036 | 2009-08-13 | Karakoram |
| Landsat5 TM | 147 | 036 | 2009-08-13 | Karakoram |
| Landsat7 ETM+ | 148 | 035 | 2001-07-21 | Karakoram |
| Landsat5 TM | 148 | 036 | 2009-08-04 | Karakoram |
| Landsat8 OLI | 149 | 035 | 2013-10-09 | Karakoram |
| Landsat5 TM | 150 | 035 & 036 | 2013-10-09 | Karakoram |
| Landsat5 TM | 149 | 033 & 034 | 2009-02-07 | Pamir |
| Landsat5 TM | 150 | 033 | 2008-10-02 | Pamir |
| Landsat5 TM | 151 | 033 | 2011-10-02 | Pamir |
| Landsat 7 ETM+ | 151 | 034 | 2000-08-24 | Pamir |
| Landsat 7 ETM+ | 152 | 033 | 2000-09-16 | Pamir |


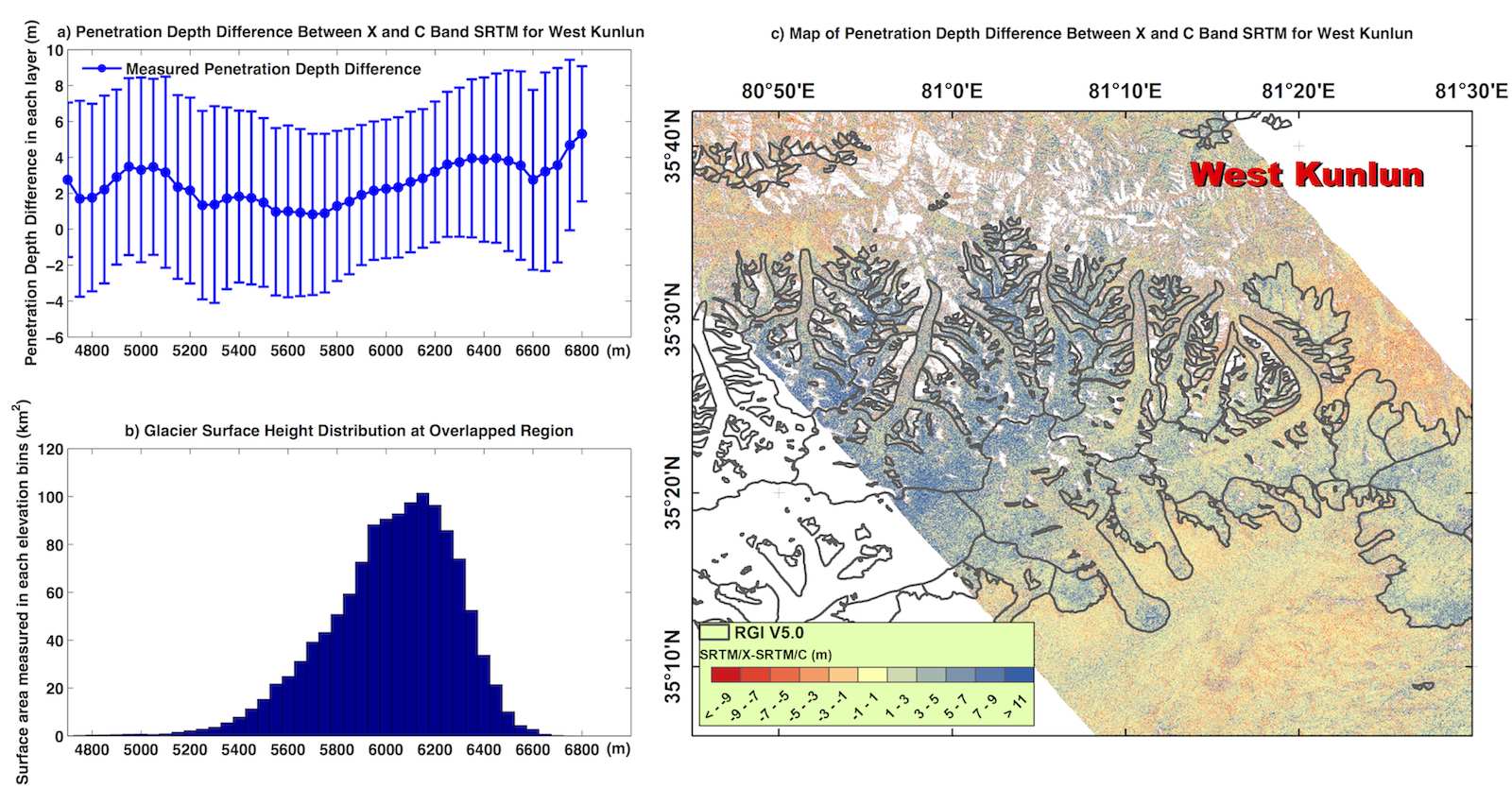


Supplementary Figure S2. a) X-band SRTM minus C-band SRTM in the West Kunlun region in each elevation bin. b) Glacier surface height distribution. c) Maps of penetration difference between X and C band SRTM in the West Kunlun region. The grey line indicates the RGI V5.0 glacier boundary. Sub-plot C was generated with ArcGIS 10.2 software (<http://www.esri.com/software/arcgis/arcgis-for-desktop>).


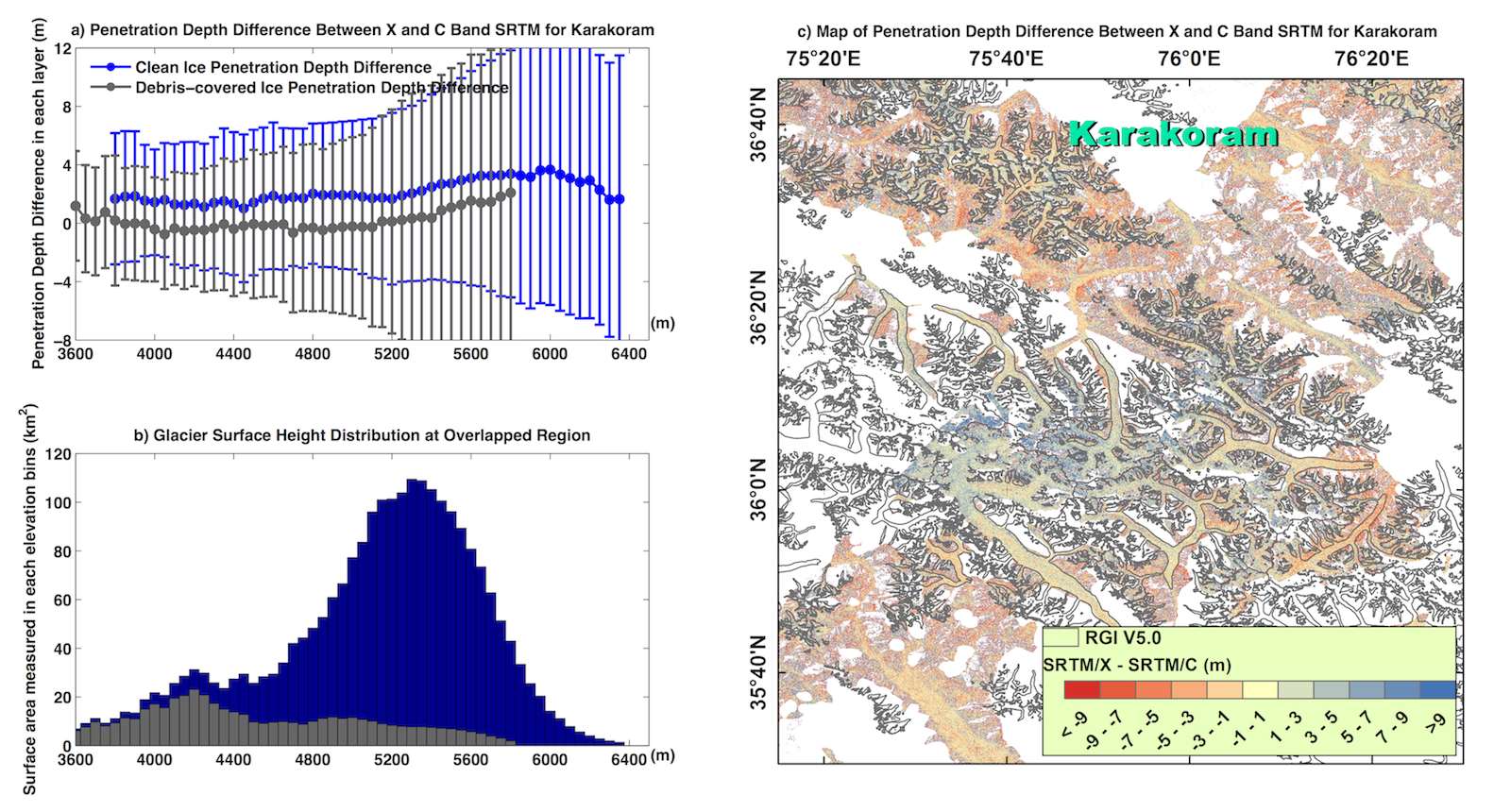


Supplementary Figure S3. a) X-band SRTM minus C-band SRTM at the Karakoram region in each elevation bin. b) Glacier surface height distribution. c) Maps of penetration difference between X and C band SRTM in the Karakoram region. The grey line indicates the RGI V5.0 glacier boundary. Sub-plot C was generated with ArcGIS 10.2 software (<http://www.esri.com/software/arcgis/arcgis-for-desktop>).


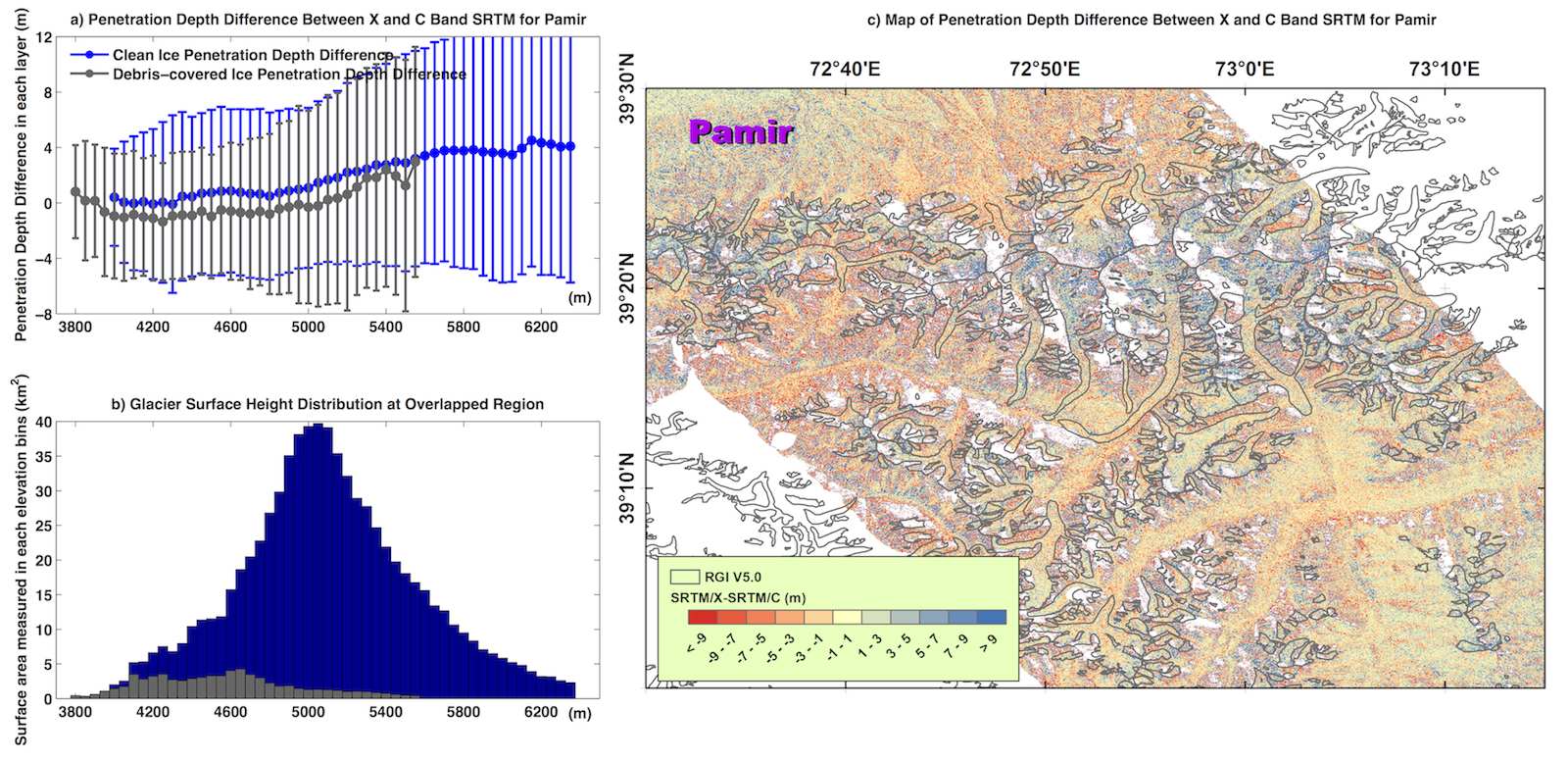


Supplementary Figure S4. a) X-band SRTM minus C-band SRTM at the Pamir region in each elevation bin. b) Glacier surface height distribution. c) Maps of penetration difference between X and C band SRTM in the Pamir region. The grey line indicates the RGI V5.0 glacier boundary. Sub-plot C was generated with ArcGIS 10.2 software (<http://www.esri.com/software/arcgis/arcgis-for-desktop>).

**2.4 Removing foreshortening overlay and shadow regions**

Foreshortening, layover and shadow regions of bistatic TSX/TDX interferograms cannot be used because of the poor quality of coherence for topographic measurement. We calculated the angle between the LOS (line of sight) and local topographic normal direction, called as local incidence angle, using formula 2:


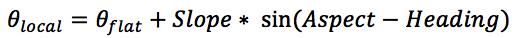
 (2)

where θ_local_ is the local incidence angle and θ_flat_ is the incidence angle on flat terrain. The heading is the orbital azimuth direction with respect to north, which is approximately 190° for descending images and -10° for ascending images. Observation with local incidence angle between 18°and 72° were accepted.

**2.5 Seasonal effect evaluation**

We selected images that were obtained in February or adjacent months to limit possible seasonal glacier height variation or snow cover affects. Summer images were not chosen because of significant precipitation and melting. In West Kunlun, we obtained images for an overlapped region from September to March (2013-09-03, 2012-10-09, 2012-12-03, 2012-12-03 and 2014-03-20). First, we formed an average TSX/TDX DEM with five pairs of images; then, we performed InSAR processing for these five pairs of images with respect to the average TSX/TDX DEM. The Chongce Glacier and the S2 Glacier are two large glaciers that are located within the overlapped region. Because the glacier mass can migrate within a single glacier over a short period, we took the average height change within one single glacier to evaluate the seasonal effect. At the Chongce Glacier, the average height differences were 1.282 m, -0.069 m, -0.174 m, and -0.394 m for the image pairs on 2013-09-03, 2012-12-03, 2012-01-30, and 2014-03-20, respectively. For the S2 Glacier, the height differences were 1.215 m, 0.067 m, -0.204 m, and -0.328 m for the image pairs on 2013-09-03, 2012-10-09, 2012-01-30, and 2014-03-20, respectively. After applying a least-square adjustment, we obtained a monthly trend of -0.07m from October to next March (Figure S6). We therefore applied a seasonal correction of -0.28 m, -0.21 m, -0.14 m, -0.07 m, 0.07 m and 0.14 m for images obtained in October, November, December, January, March and April, respectively. Since it is possible that summer does not end in September, we did not use images obtained in September. Glacier height changes with respect to the average TSX/TDX DEM and DEM on 2012-12-03 are shown in figure S5.


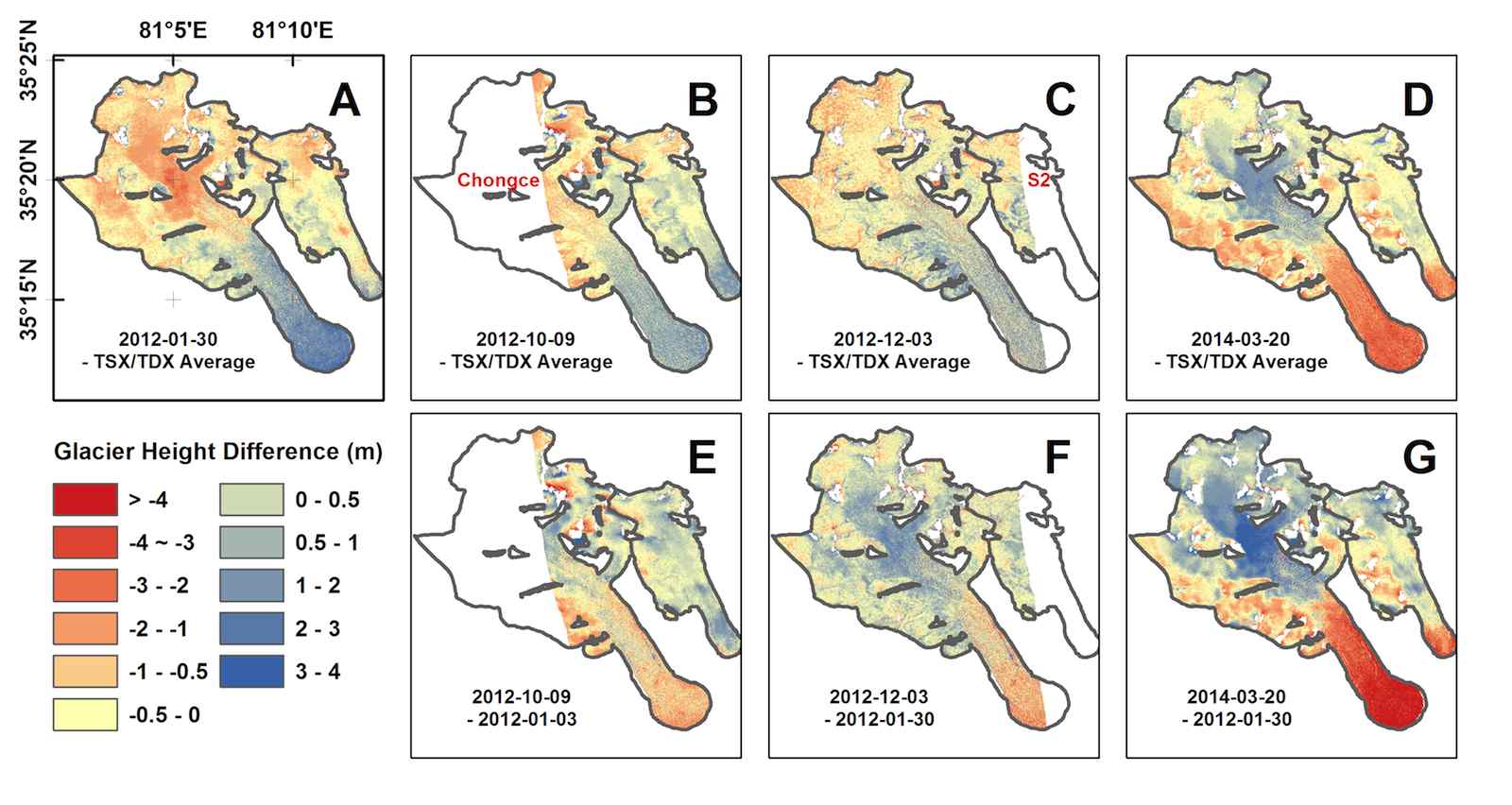


Supplementary Figure S5. Seasonal variation of glacier height. A, B, C, and D present the glacier height difference between specific dates and average TSX/TDX DEM. E, F and G present glacier height differences between specific dates and DEM on 2012-01-03. This figure was generated with ArcGIS 10.2 software (<http://www.esri.com/software/arcgis/arcgis-for-desktop>).

**
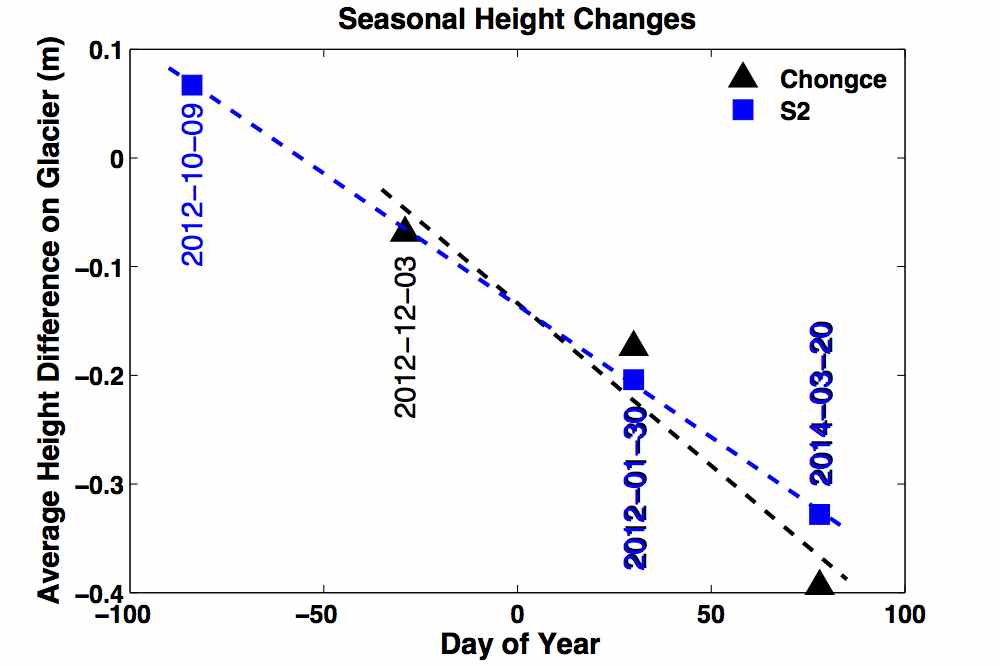
**

Supplementary Figure S6. Least-square adjustment to glacier height differences for the Chongce and the S2 glacier at the West Kunlun.

Penetration depth was corrected by different values per 50-m elevation bin according to the output of section 2.3. Then, we calculated the average decadal glacier height changes using the glacierized area in each elevation bin as a weighting function. It is presumed that the glacier height changes were the same at the same elevation bin in the same glacier, sub-region or sub-group. When calculating glacier height changes, some parts can be covered by the interferograms more than once; therefore, averaging was conducted to calculate the height changes.

**2.6 Transferring glacier height changes to glacier mass balance**

For each sub-region, we produced a mosaic of glacier height changes derived by different frames. Some parts can be covered by the interferograms more than once; therefore, averaging was conducted to calculate the height changes. For each sub-region, we calculated the average height changes in each 50-m elevation bin, and then according to glacierized area in each 50-m elevation bin, we calculated total glacier volume changes following formula 3.


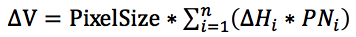
 (3)

Where V is total glacier volume changes, H_i_ is average glacier height changes observed at ith elevation bin, PN_i_ is total glacierized pixel numbers in ith elevation bin. Average glacier height changes are calculated by dividing V by total glacierized area following formula 4.


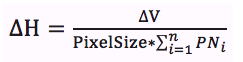
 (4)

We convert annual average glacier height changes into glacier mass balance with a density assumption of 850 ± 60 kg/m^3^. Surging and quiescent glaciers are treated separately, and their mass balance are added to the regional glacier mass balance using area as a weight. For West Kunlun, West Kunlun Extent A-G and Eastern Pamir, RGI V5.0 applied satellite images obtained after 2010 to identify its boundary. For the remainder of regions, satellite images mostly obtained in 1999, 2000 and 2001 were applied. To check the average glacierized area, we obtained Landsat images obtained around the end of our study period for Karakoram, Hindu Kush and Pamir, Western and Central Pamir and Landsat images obtained around 2000 for the rest of the areas. Basic information of these Landsat images is listed in table S3. It is found that almost all normal type glaciers and several surge-type glaciers have stable glacier fronts during our study periods. We manually identify their glacier front changes. For both surge-type glaciers and quiescent glaciers, a maximum area is applied in calculating glacier volume changes, and in transferring glacier volume changes to average glacier height changes, average area is applied. The average area equals to area in RGI V5.0 dataset and plus or minus half of the changing area manually identified with Landsat images in Table S3.

Supplementary Table S3. Basic information of Landsat images applied for identifying clean-ice glaciers.

| Satellite & Payload | Path | Row | Observation Date | Sub-region |
| --- | --- | --- | --- | --- |
| Landsat 7 ETM+ | 145 | 035 &036 | 2000-12-04 | West Kunlun |
| Landsat 7 ETM+ | 147 | 035 | 1999-12-16 | West Kunlun Extent |
| Landsat 7 ETM+ | 146 | 035 | 2000-10-08 | West Kunlun Extent |
| Landsat 8 OLI | 147 | 035 & 036 | 2013-12-13 | Karakoram |
| Landsat 8 OLI | 148 | 035 & 036 | 2013-07-14 | Karakoram |
| Landsat 8 OLI | 149 | 035 & 036 | 2013-10-09 | Karakoram |
| Landsat 8 OLI | 150 | 034 & 036 | 2014-10-03 | Hindu Kush |
| Landsat 8 OLI | 151 | 033 | 2014-08-07 | Western & Central Pamir |
| Landsat 8 OLI | 152 | 033 | 2013-09-12 | Western Pamir |
| Landsat 8 OLI | 149 | 033 & 034 | 2000-10-29 | Eastern Pamir |

**2.7 Error estimation and cross validation**

We measured glacier height changes instead of directly measuring the glacier mass balance. Basically, we evaluated errors for each item using the principle of error propagation. The error in glacier height change consists of a systemic component and a random component. The systemic component contains a penetration estimation error, seasonal effects, and a datum chosen for the off-glacier region. To estimate the error of the difference between the C- and X-band SRTM for each sub-region, we calculated the standard deviation for each elevation bin (every 50 m) and then weighted the RMSE of the penetration depth. The error penetration depth estimation was then calculated as formula 5


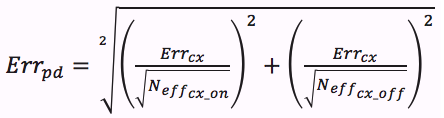
 (5)

where Err_pd_ is the error of the penetration depth, Err_cx_ is the weighted RMSE of the penetration depth for one pixel, Neffcx_on is the effective measurement number on glaciers while Neffcx_off is effective measurement number in the off-glacier area. (
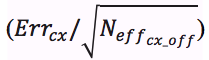
 ) is the error in estimating datum differences between the two DEMs. C- and X-band DEMs were released in the EGM96 and WGS84 vertical datum, respectively. After removing the datum difference, the vertical offset varies from -20 to 20 m^14^. Datum errors are larger than relative errors, especially for X-band SRTM^14^. Therefore we align the off-glacier region to remove such datum differences and datum errors in both the C- and X-band SRTM dataset. We applied a correlation distance of 1000 m for the differencing process of C- and X-band SRTM by calculating a variogram in the off-glacier region. The Err_cx_ values for West Kunlun, Karakoram and Pamir are 4.35 m, 6.33 m and 7.24 m, respectively, with Err_pd_ values of 0.127 m, 0.170 m and 0.263 m. Seasonal snow can affect the glacier height measurement; we added 0.15 m/month to those images not observed in February when SRTM was obtained. Because InSAR is a relative measurement, a reference region needs to be defined. We selected the entire off-glacier region, except for water bodies, as the reference region for each frame, in which it was presumed no height changes occurred. For each frame of the bistatic TSX/TDX images, we plotted a histogram of the height difference in the off-glacier region, which generally followed a normal distribution (figure S7). The error of the data used for selecting the bistatic D-InSAR was then calculated with formula 6:


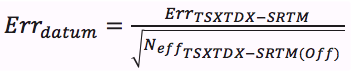
 (6)

where Err_TSXTDX-SRTM_ is the standard deviation of the height difference of the off-glacier region and Neff_TSXTDX-SRTM(off)_ is the effective number of measurements for the off-glacier region, assuming 500 m as an correlation distance. Then, for each frame, the systemic part of error was calculated with formula 7:


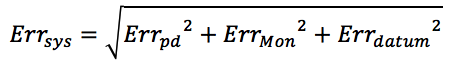
 (7)


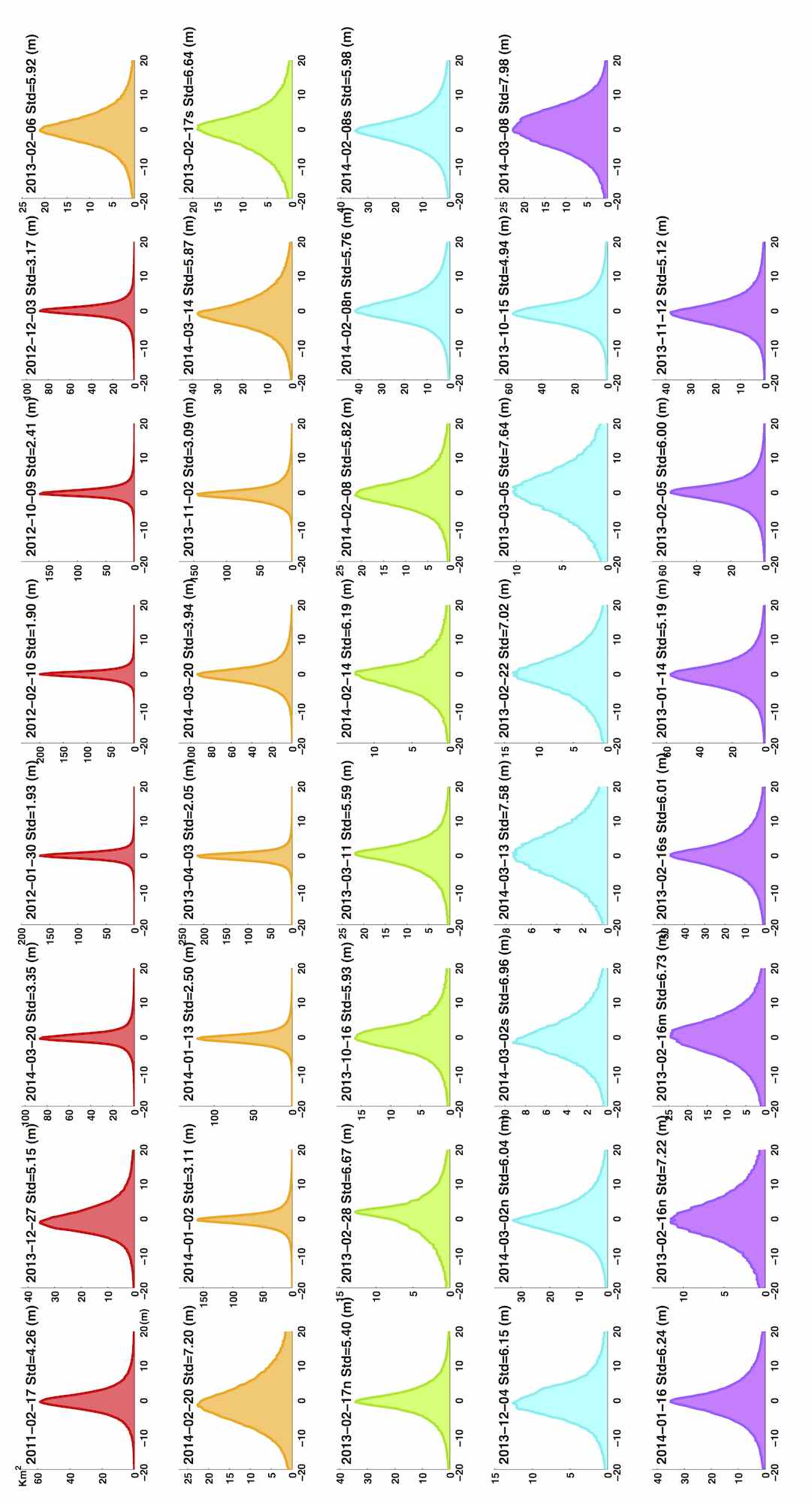


Supplementary Figure S7. Histogram of height differences for the off-glacier region, for each pair of bistatic SAR images. Different colours of histograms indicate images for different groups of West Kunlun (Red), West Kunlun Extent (Orange), Eastern Karakoram (Green), West Karakoram and Hindu Kush (Cyan) and Pamir (Purple). In each sub-plot, the X-axis indicates height differences and the Y-axis indicates the area measured in units of square kilometres. Std in each sub-plot is Err_TSXTDX-SRTM_ for each acquisition.

The random error depends on E_rrTSXTDX-SRTM_ and the area measured in the glacier region, which is calculated with formula 8:


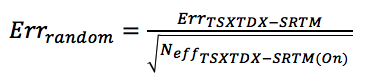
 (8)

where Neff_TSXTDX-SRTM(On)_ is the effective number of measurements, assuming 500 m as the correlation distance, for the glacier area for each sub-region, frame or one single glacier. Err_TSXTDX-SRTM_ is different for each frame as their perpendicular baselines and height ambiguity are different. Usually, the frames with longer perpendicular baselines give a smaller standard deviation. The histogram of the height difference deviation in the off-glacier region indicates a higher measurement precision than differencing processing of SRTM and SPOT/HRS DEM^1, 2^.

Propagating error of absolute height changes to error of height changing annual rates follows formula 9:


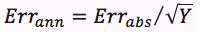
 (9)

where Err_ann_ is error for height changing annual rate, Err_abs_ is error of absolute height changes, which could be composed by result of formula 7 and 8. Y is duration between SRTM acquisition time and TSX/TDX acquisition time in unit of year.

Propagation error of height changing annual rates to glacier mass balance by presuming density of 850 ± 60 kg/m^3^ follows formula 10:


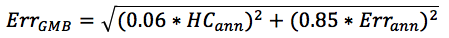
 (10)

where Err_GMB_ is error for annual geodetic glacier mass balance, and HC_ann_ is annual glacier height changing rate.

In addition, we performed cross validation to the glacier height difference results using two regions with the same track and across tracks with overlapping area coverage. Figure S8 presents the cross validation results of the West Kunlun region. Because the bistatic TSX/TDX was obtained at different points in time, we normalized the height changes into a decadal scale by presuming a linear height change rate. For the same track validation, images obtained on 2012-01-30 and 2012-12-03 were used, and for cross track validation, images obtained on 2011-02-17 and 2013-12-27 were used; both validation sets showed good agreement. Theoretically, height changes should be 1.414 times the RMSE of the TanDEM DEM, which is 2 m (https://directory.eoportal.org/web/eoportal/satellite-missions/t/tandem-x). Our results are similar to the DLR-claimed precision.


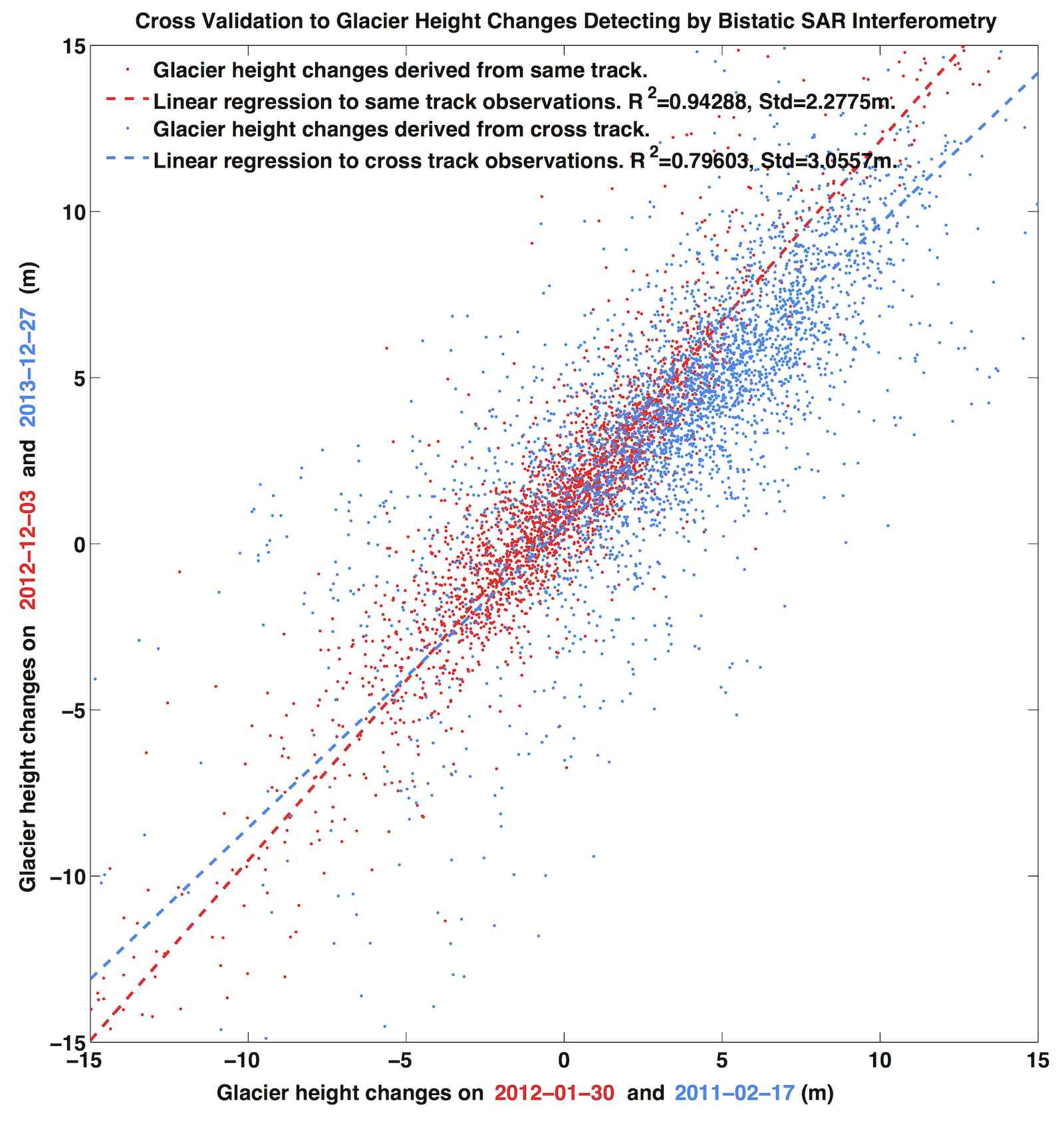


Supplementary Figure S8. Cross validation of the glacier height changes detected by bistatic InSAR. Red points are pixels in the overlap region covered by the same track coverage. Blue points are pixels in the overlap region covered by the cross track coverage.

**3. Detail Results of Glacier Height Changes**

In supplementary figures S9 to S17 we present maps of decadal glacier height changes for each region in high resolution.


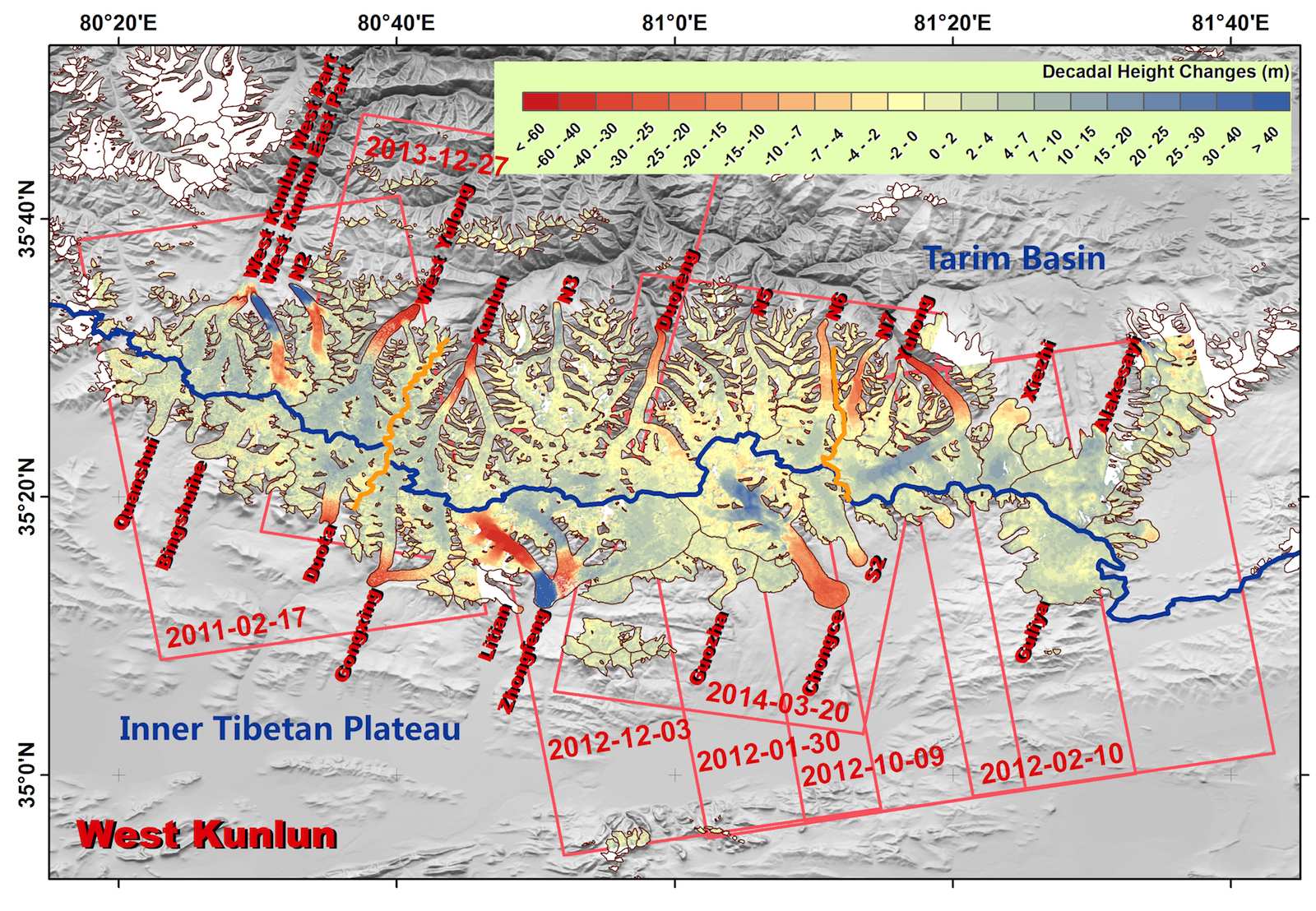


Supplementary Figure S9. Decadal glacier height changes for West Kunlun. Glacier names or IDs were marked at glacier tongues following Yasuda’s research^15^ on glacier flow rates. We separate glaciers into a western group, central group and eastern group. Divergence is shown in yellow; clearly it accumulated more mass at firn basins for western and eastern part than central part. The watershed (blue line) also separates the West Kunlun into the Tarim Basin and the Inner Tibetan Plateau (ITP) region. This figure was generated with ArcGIS 10.2 software (<http://www.esri.com/software/arcgis/arcgis-for-desktop>).


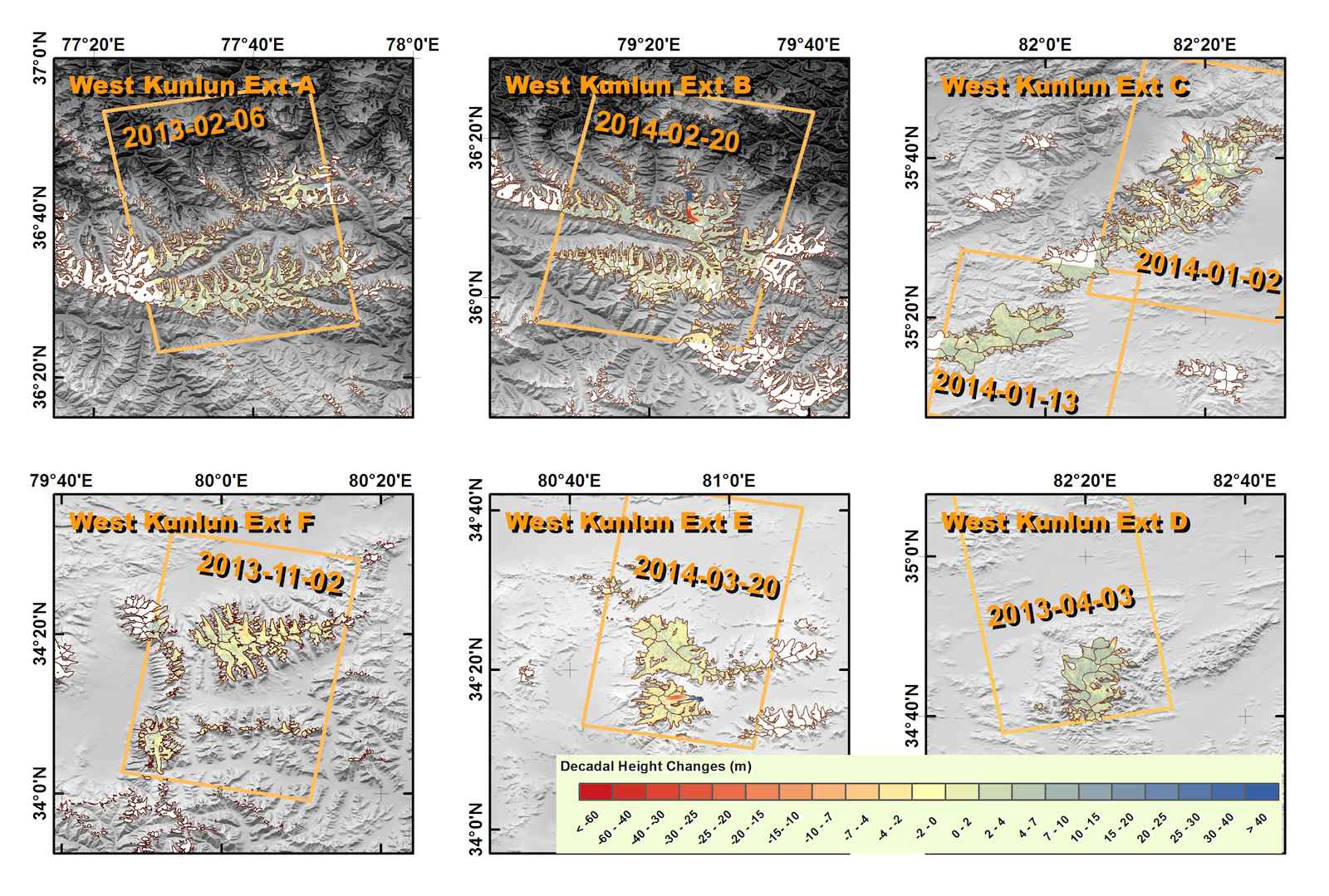


Supplementary Figure S10. Decadal height changes for the West Kunlun Extent region. No large glacier was included in this region. This figure was generated with ArcGIS 10.2 software (<http://www.esri.com/software/arcgis/arcgis-for-desktop>).


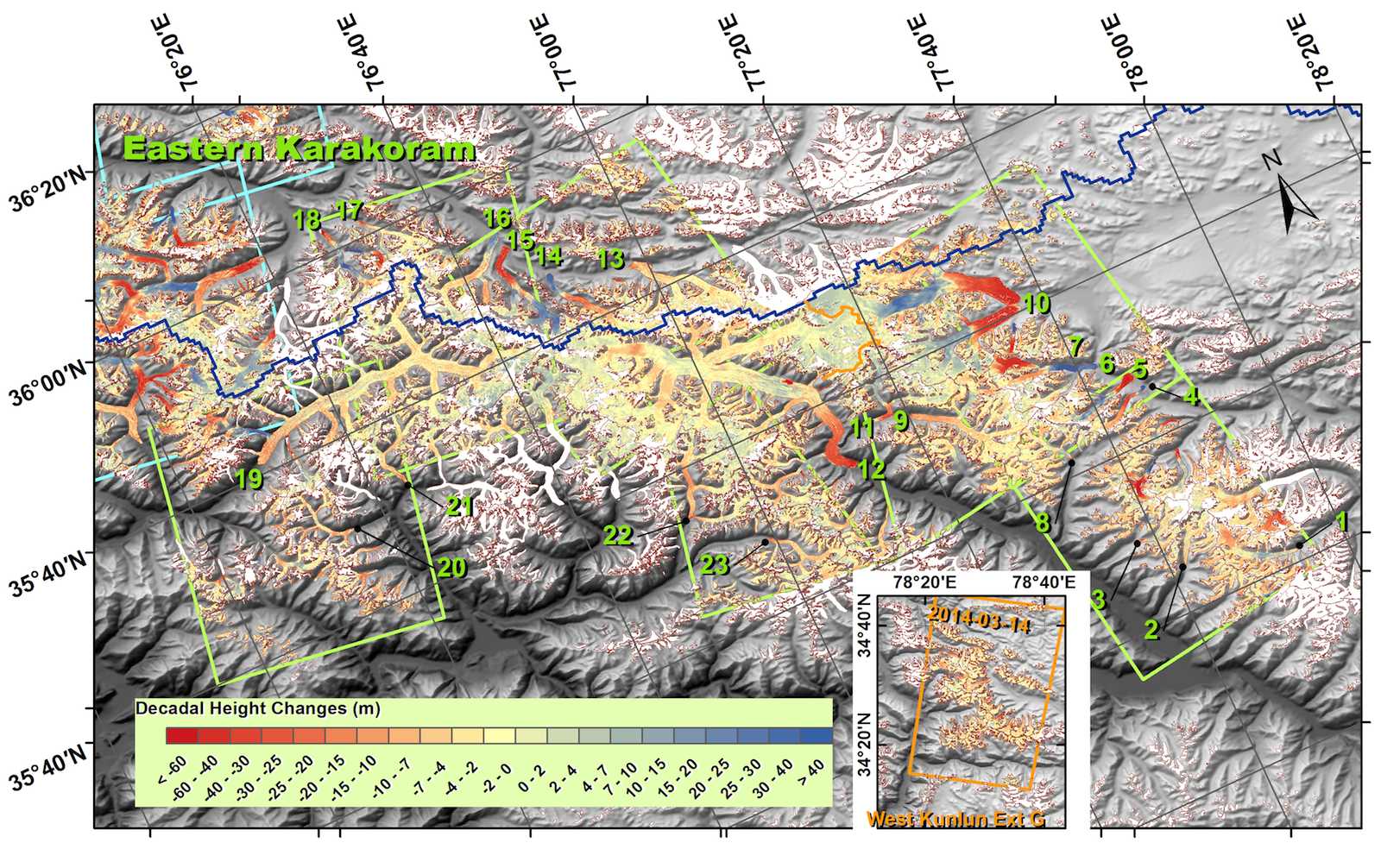


Supplementary Figure S11. Decadal glacier height changes for Eastern Karakoram and the West Kunlun Ext G. The West Kunlun Ext G region is shown in this figure due to its proximity to the Eastern Karakoram. We rotate the main maps because of glaciers distribution. Glacier names or IDs were marked at glacier tongues. We separate the glaciers into central and eastern group mainly regarding the ridge between the Siachen (No.12) and the Rimo (No.10) glaciers and the ridge between the Siachen and the North Terong (No.11) glaciers as divergence, shown with a yellow line. The watershed (plotted with a deep blue line) also separates Eastern Karakoram into the Upper Tarim region (north) and the Upper Indus region (south). This figure was generated with ArcGIS 10.2 software (<http://www.esri.com/software/arcgis/arcgis-for-desktop>).


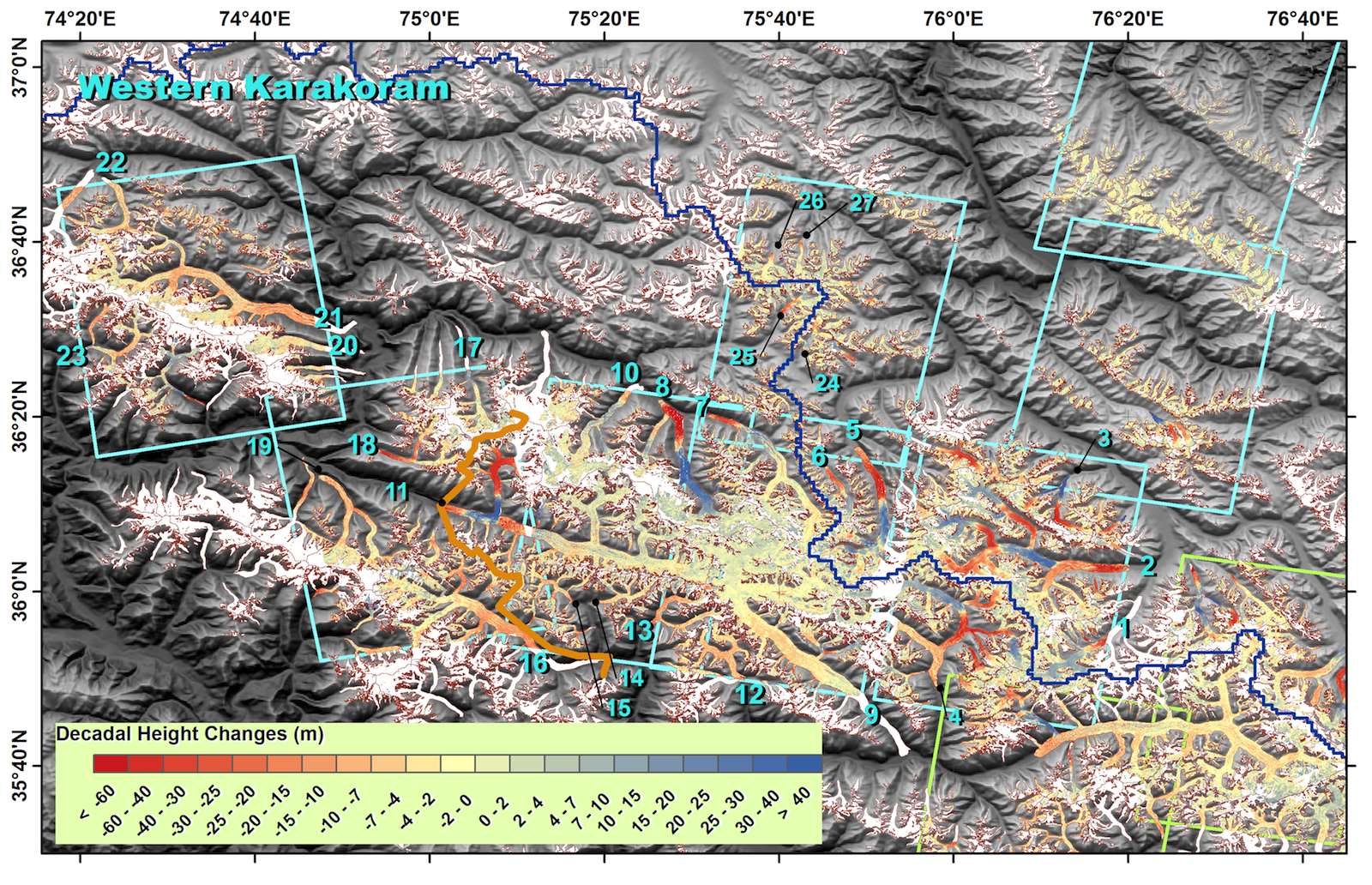


Supplementary Figure S12. Decadal glacier height changes for Western Karakoram. Glacier names or IDs were marked at glacier tongues. We separate the glaciers into western and central groups with divergence shown as a yellow line. All glaciers west of the Hispar Glacier (No.11) were classified into a western group. The watershed (plotted with a deep blue line) also separates Western Karakoram into the Upper Tarim region (north) and the Upper Indus region (south). This figure was generated with ArcGIS 10.2 software (<http://www.esri.com/software/arcgis/arcgis-for-desktop>).


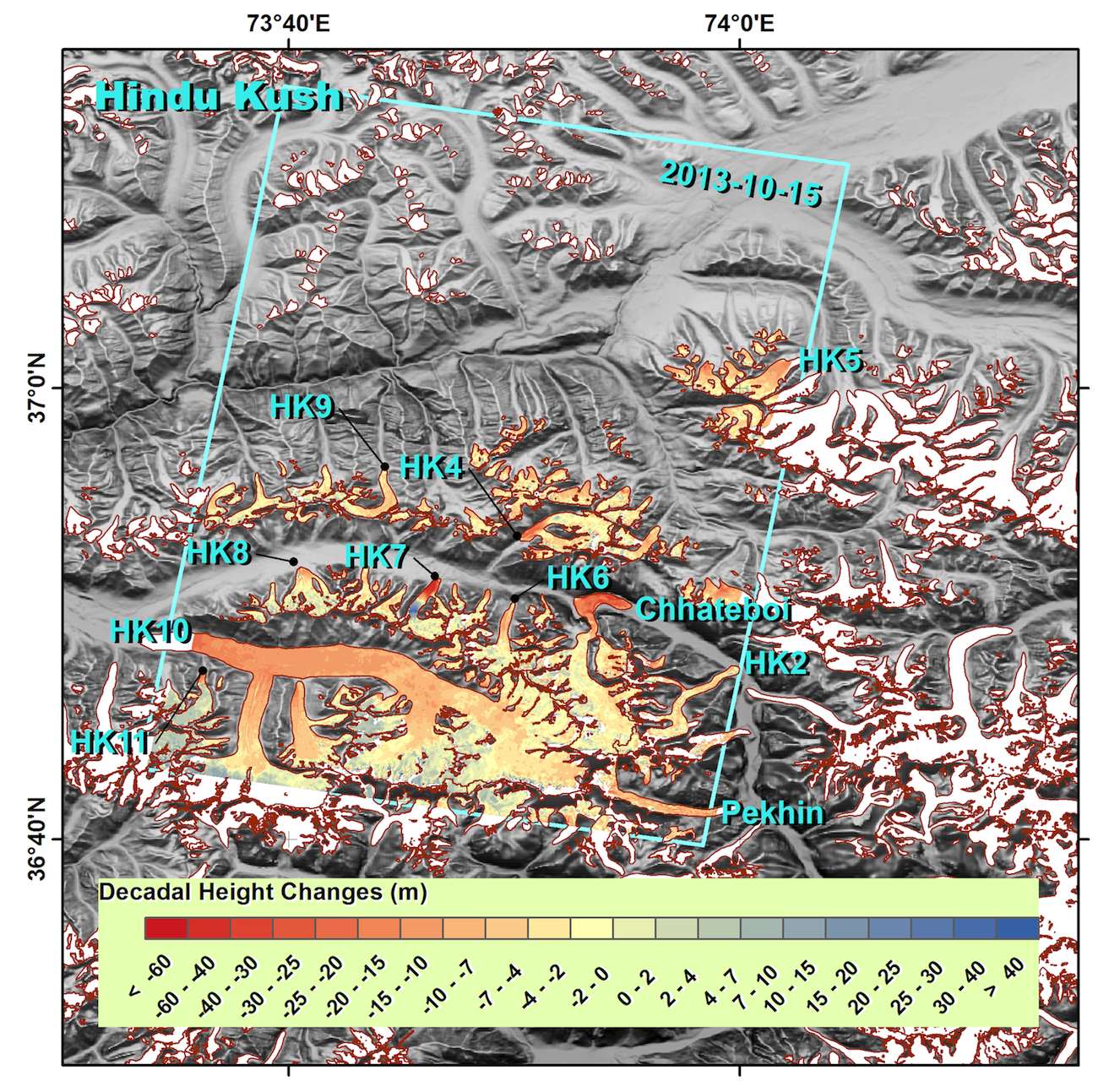


Supplementary Figure S13. Decadal glacier height changes for the Hindu Kush region. Glacier names or IDs were marked at glacier tongues. This figure was generated with ArcGIS 10.2 software (<http://www.esri.com/software/arcgis/arcgis-for-desktop>).


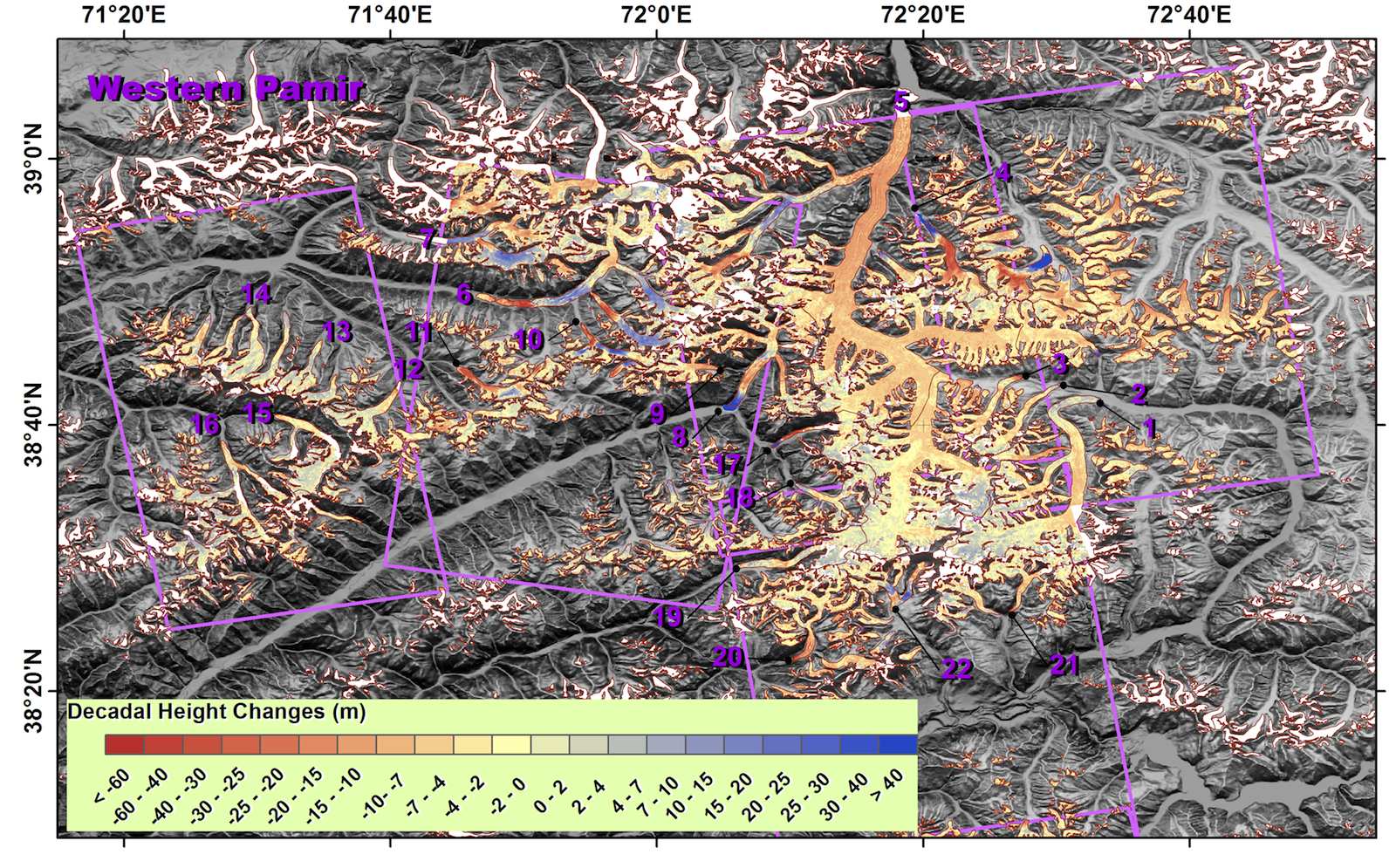


Supplementary Figure S14. Decadal glacier height changes for Western Pamir. Glacier names or IDs are marked at glacier tongues. The Fedchenko (No.5) is the largest and longest glacier in this region. We separate the Western Pamir into two different groups for analysis by regarding the Fedchenko glacier as a divide. This figure was generated with ArcGIS 10.2 software (<http://www.esri.com/software/arcgis/arcgis-for-desktop>).


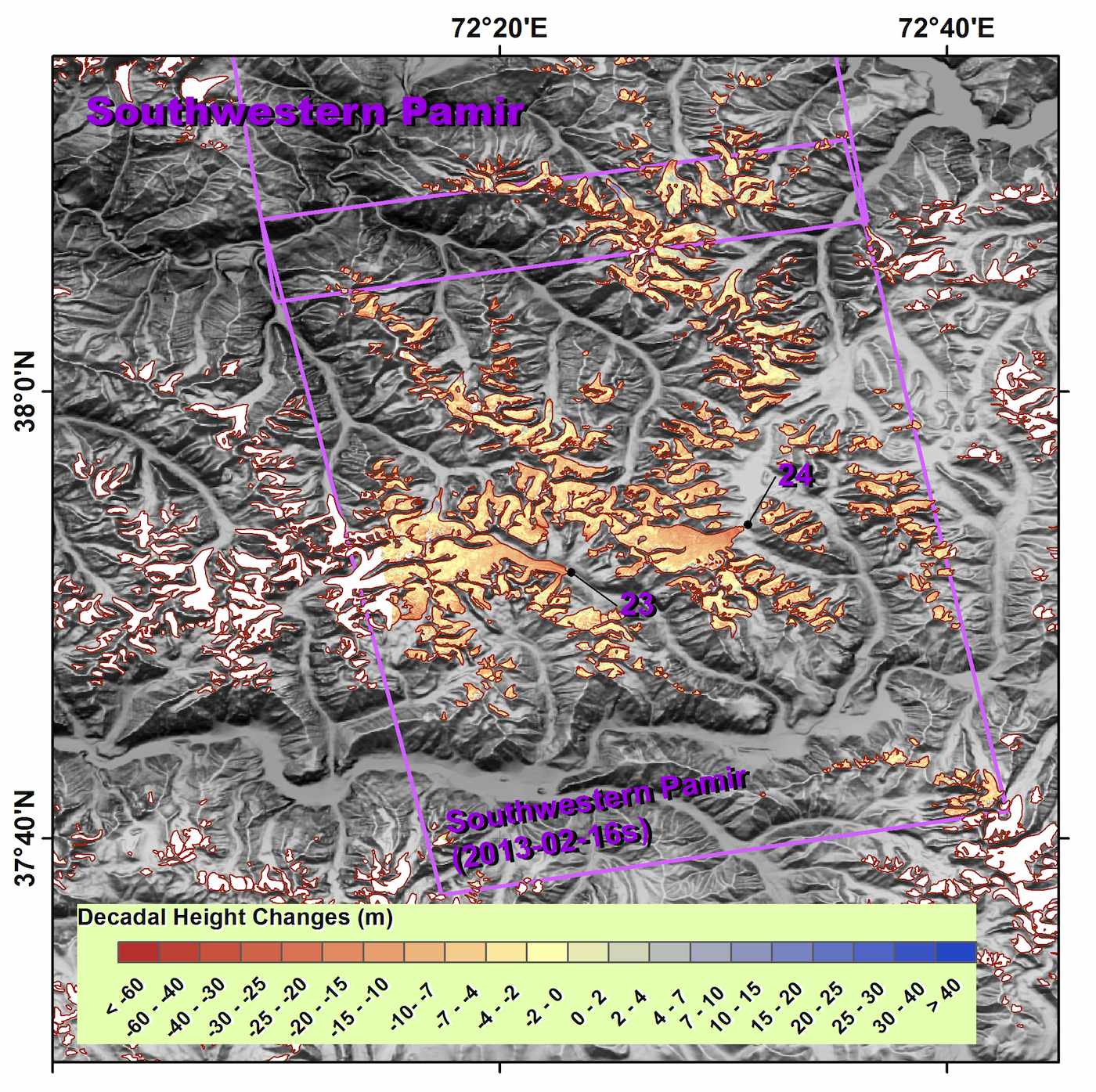


Supplementary Figure S15. Decadal glacier height changes for Western Pamir (Southern part). This figure was generated with ArcGIS 10.2 software (<http://www.esri.com/software/arcgis/arcgis-for-desktop>).


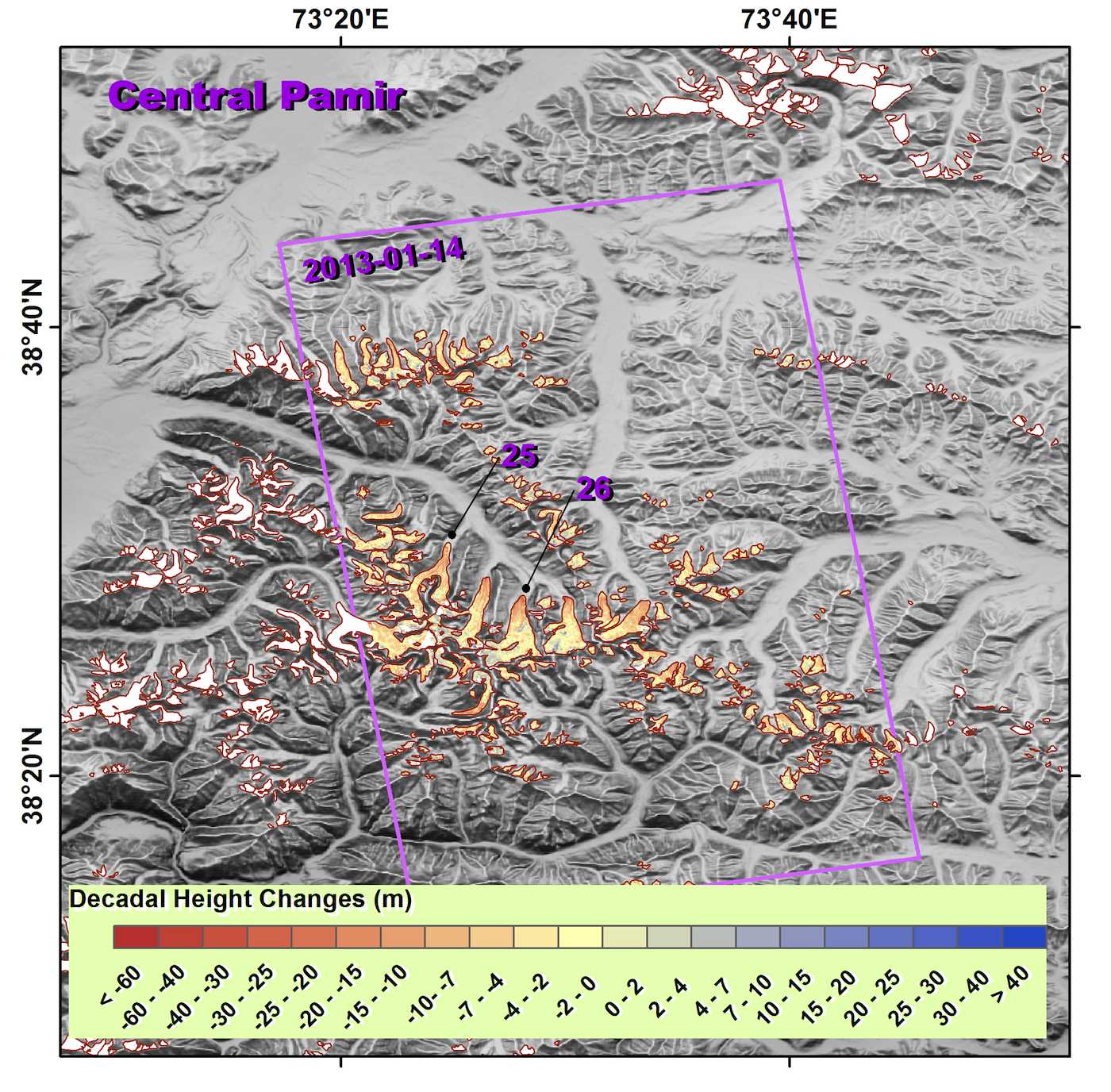


Supplementary Figure S16. Decadal glacier height changes for Central Pamir. This figure was generated with ArcGIS 10.2 software (<http://www.esri.com/software/arcgis/arcgis-for-desktop>).


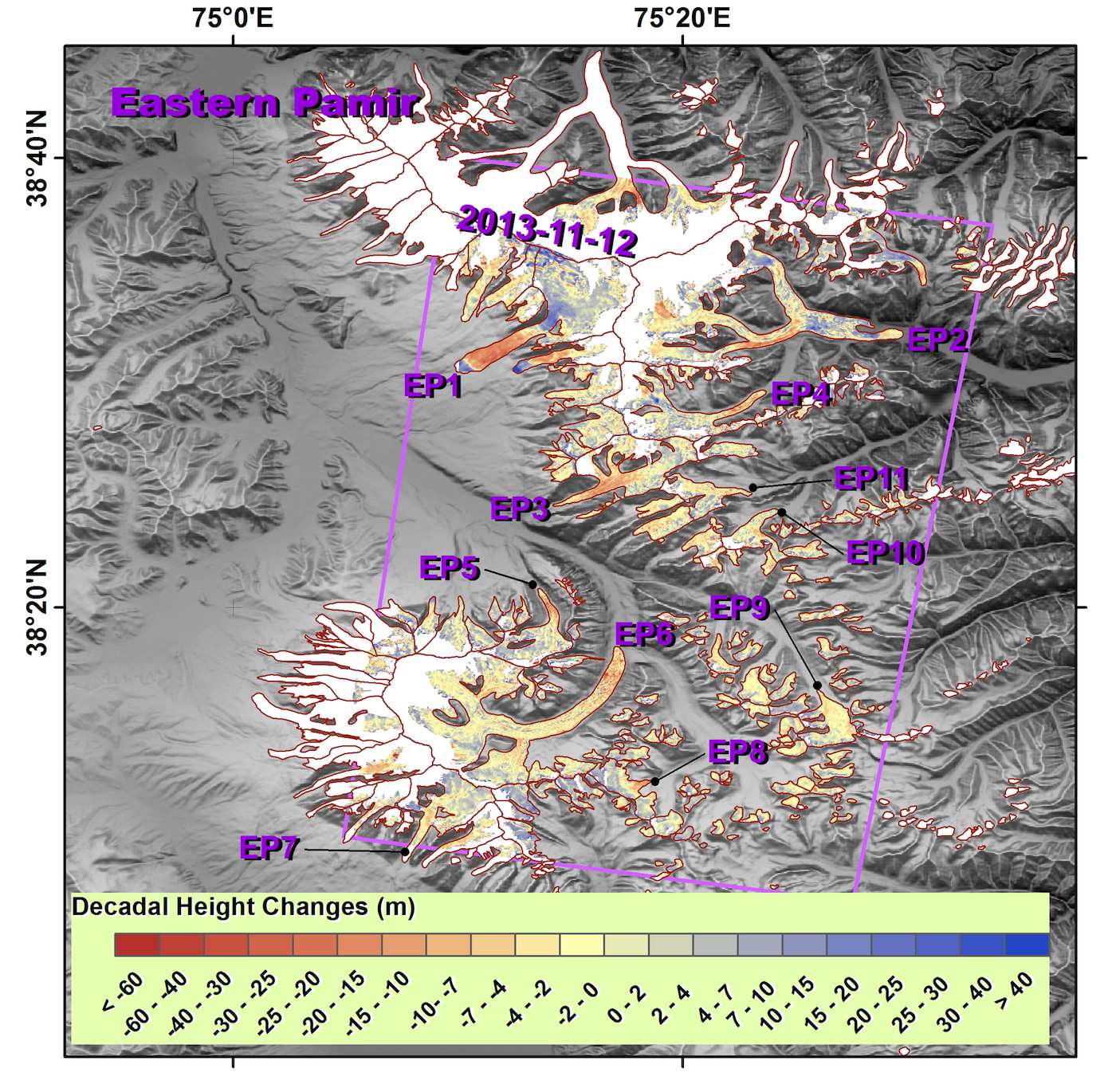


Supplementary Figure S17. Decadal glacier height changes for Eastern Pamir. Glacier IDs are marked at glacier tongues. This region belongs to the Upper Tarim Basin. This figure was generated with ArcGIS 10.2 software (<http://www.esri.com/software/arcgis/arcgis-for-desktop>).

Supplementary Table S4. Glacier mass balance in unit of m w.e. yr^-1^ for each sub-region, each group, and several great glaciers.

| Region or sub-region | Mass balance in unit of w.e. yr^-1^ | Annual height changes in unit of w.e. yr^-1^ (Accumulation zone) | Annual height changes in unit of w.e. yr^-1^ (Ablation zone) |
| --- | --- | --- | --- |
| West Kunlun (WK) | 0.128±0.055 | 0.190±0.057 | -0.038±0.055 |
| WK South (ITP) | 0.095±0.057 | 0.141±0.058 | 0.090±0.061 |
| WK North (Tarim) | 0.144±0.057 | 0.192±0.058 | 0.045±0.058 |
| WK East | 0.174±0.058 | 0.231±0.061 | 0.064±0.062 |
| WK Central | 0.079±0.056 | 0.114±0.057 | -0.001±0.059 |
| WK West | 0.201±0.061 | 0.290±0.064 | 0.072±0.067 |
| WK Extent A | 0.145±0.056 | 0.133±0.067 | 0.163±0.075 |
| WK Extent B | 0.211±0.056 | 0.118±0.082 | 0.272±0.073 |
| WK Extent C (2014-01-02) | 0.356±0.056 | 0.369±0.060 | 0.330±0.064 |
| WK Extent C (2014-01-13) | 0.329±0.054 | 0.345±0.057 | 0.322±0.060 |
| WK Extent D | 0.363±0.065 | 0.436±0.072 | 0.321±0.066 |
| WK Extent E | 0.078±0.054 | 0.092±0.061 | 0.061±0.065 |
| WK Extent F | 0.043±0.078 | 0.088±0.080 | -0.100±0.087 |
| WK Extent G | -0.286±0.067 | -0.136±0.078 | -0.473±0.084 |
| East Karakoram (EKK) | -0.101±0.058 | -0.017±0.059 | -0.261±0.062 |
| EKK (East) | -0.136±0.061 | -0.076±0.061 | -0.600±0.082 |
| EKK (Central) | -0.058±0.058 | 0.044±0.061 | -0.189±0.061 |
| EKK (Indus) | -0.124±0.058 | -0.076±0.059 | -0.259±0.062 |
| EKK (Tarim) | -0.048±0.060 | -0.046±0.067 | -0.058±0.073 |
| West Karakoram (WKK) | 0.000±0.064 | 0.116±0.068 | -0.150±0.067 |
| WKK (Central) | 0.018±0.065 | 0.119±0.067 | -0.093±0.067 |
| WKK (West) | -0.217±0.071 | -0.035±0.104 | -0.216±0.075 |
| WKK (Indus) | -0.026±0.064 | 0.129±0.070 | -0.148±0.067 |
| WKK (Tarim) | -0.020±0.064 | 0.063±0.069 | -0.195±0.077 |
| Hindu Kush | -0.137±0.085 | 0.182±0.112 | -0.214±0.088 |
| Western Pamir (WPMR) | -0.118±0.062 | 0.046±0.064 | -0.194±0.067 |
| Western Pamir (West to Fedchenko) | -0.027±0.064 | 0.095±0.068 | -0.078±0.070 |
| Western Pamir (Southeast to Fedchenko) | -0.141±0.067 | -0.067±0.070 | -0.276±0.077 |
| Central Pamir | -0.100±0.074 | 0.042±0.085 | -0.242±0.080 |
| Eastern Pamir | 0.124±0.074 | 0.179±0.088 | 0.047±0.086 |
| Duofeng (WK) | 0.040±0.062 | 0.080±0.064 | -0.108±0.084 |
| Siachen (EKK) | -0.083±0.066 | 0.058±0.066 | -0.271±0.069 |
| Baltoro (EKK) | -0.007±0.061 | 0.146±0.080 | -0.155±0.071 |
| Biafo (WKK) | 0.044±0.079 | 0.148±0.103 | -0.023±0.082 |
| Hispar (WKK) | -0.019±0.082 | 0.105±0.164 | -0.063±0.080 |
| Chogo Lungma (WKK) | -0.130±0.092 | 0.134±0.164 | -0.258±0.097 |
| Batura (WKK) | -0.263±0.069 | -0.148±0.158 | 0.340±0.095 |
| Fedchenko (WPMR) | -0.147±0.069 | -0.028±0.076 | -0.420±0.078 |
| Kekesayi (EPMR) | 0.055±0.101 | 0.188±0.192 | -0.079±0.143 |

In supplementary figures S18 ~ S38 we plot decadal height changes and their relationship with elevations at different regions, sub-regions and several great glaciers. The error bars in these figures do not indicate the error but the standard deviation in every elevation bins as glacier height changes in one certain elevation bin is not supposed to be the same. Because of voids in SRTM, looking geometry, de-correlation, and unwrapping errors, it is impossible to measure glacier height changes everywhere. We therefore plot the area measured and area intended to be measured. We did not include large surging glaciers for plotting because its glacier height changes behave differently compared to normal glaciers.


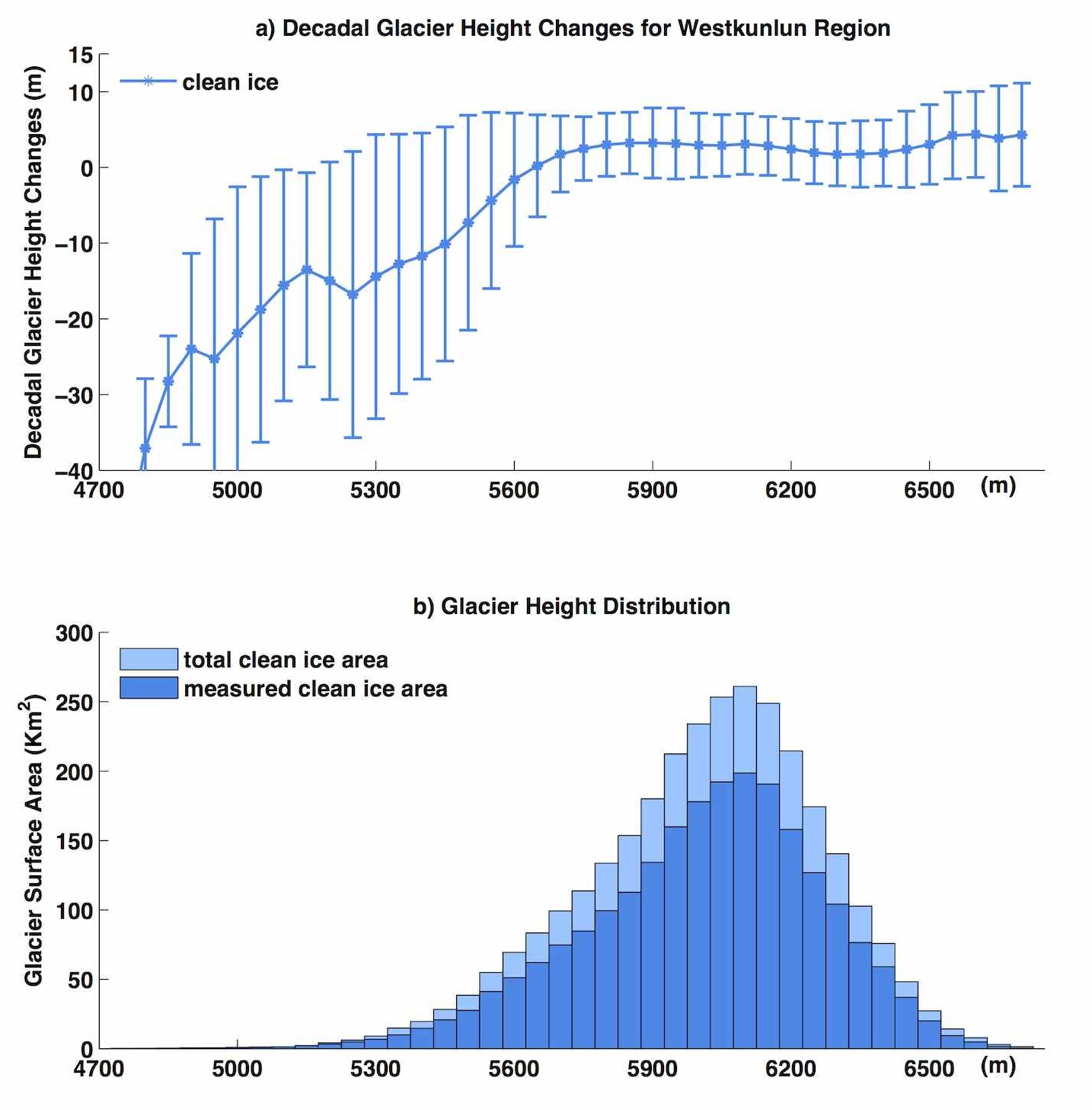


Supplementary Figure S18. Glacier height changes (a) and height distributions (b) at each elevation bin for West Kunlun. Error bars only indicate standard deviation of glacier height changes in each elevation bin.


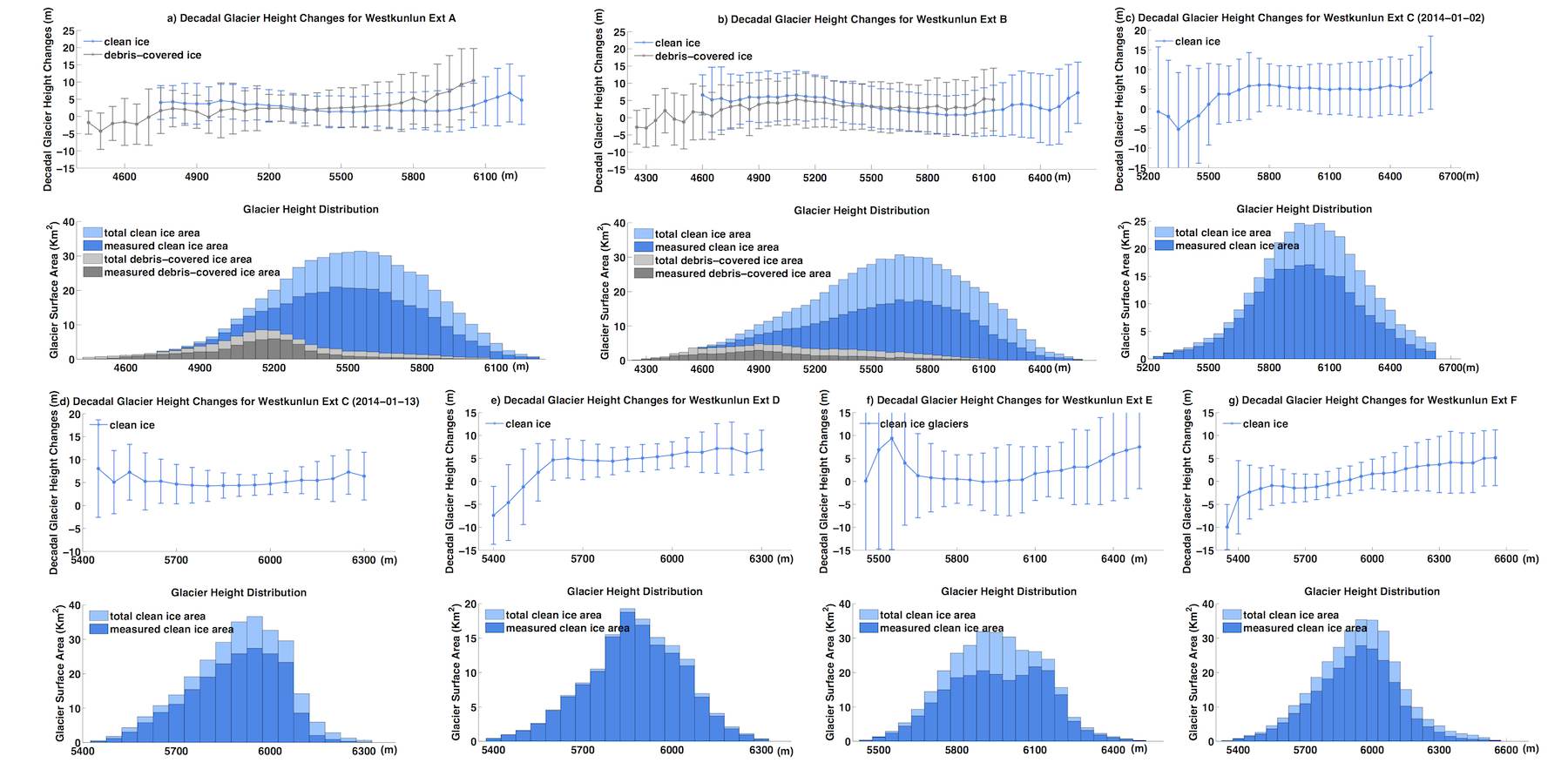


Supplementary Figure S19. Same as Figure S18 but for West Kunlun Extent A-F. For Ext A & B, the grey colour indicates glacier height changes or elevation distributions on debris-covered ice. West Kunlun C is separated into 2 sub-plots because two images have coverage.


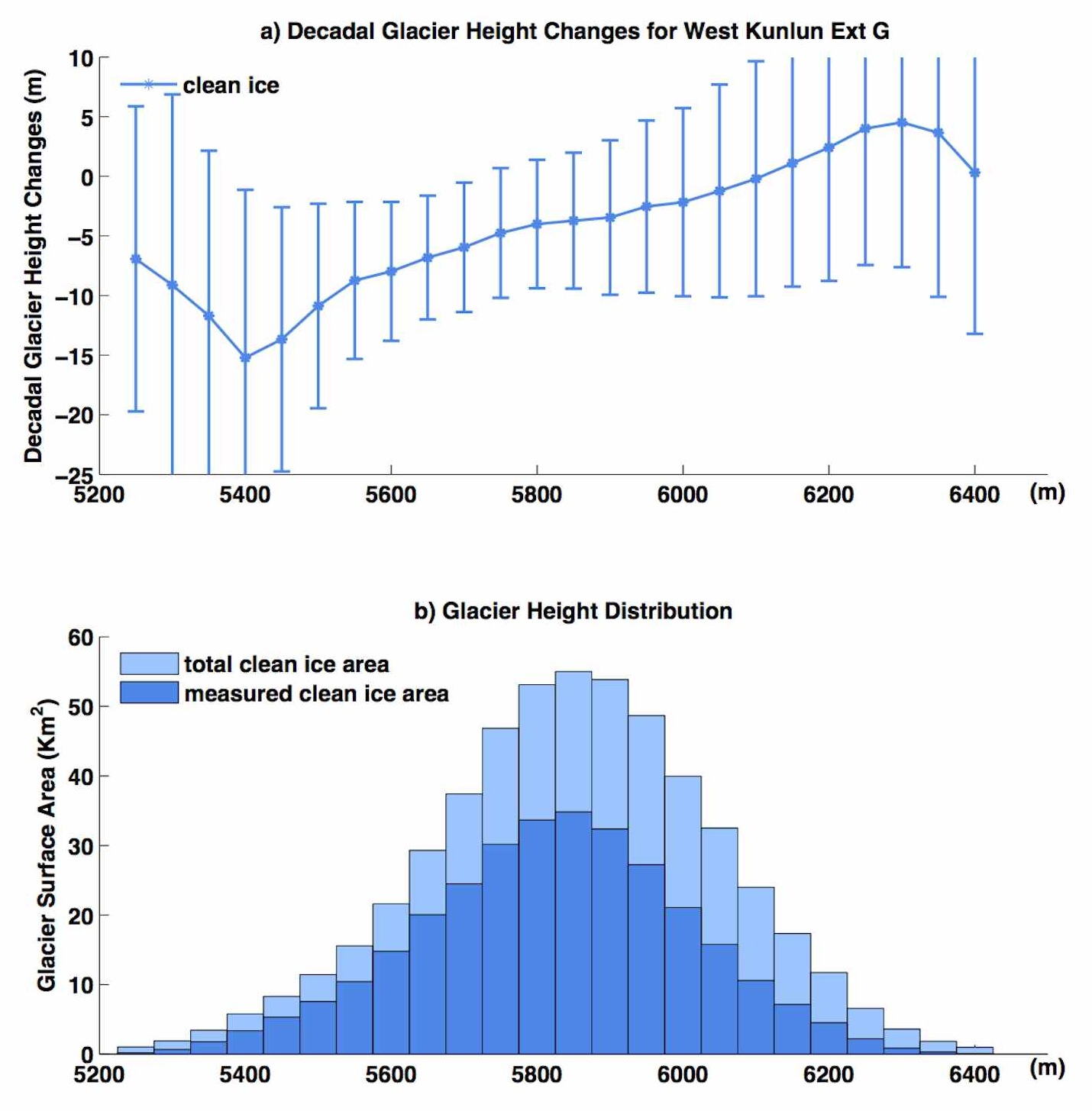


Supplementary Figure S20. Same as Figure S18 but for West Kunlun Extent G.


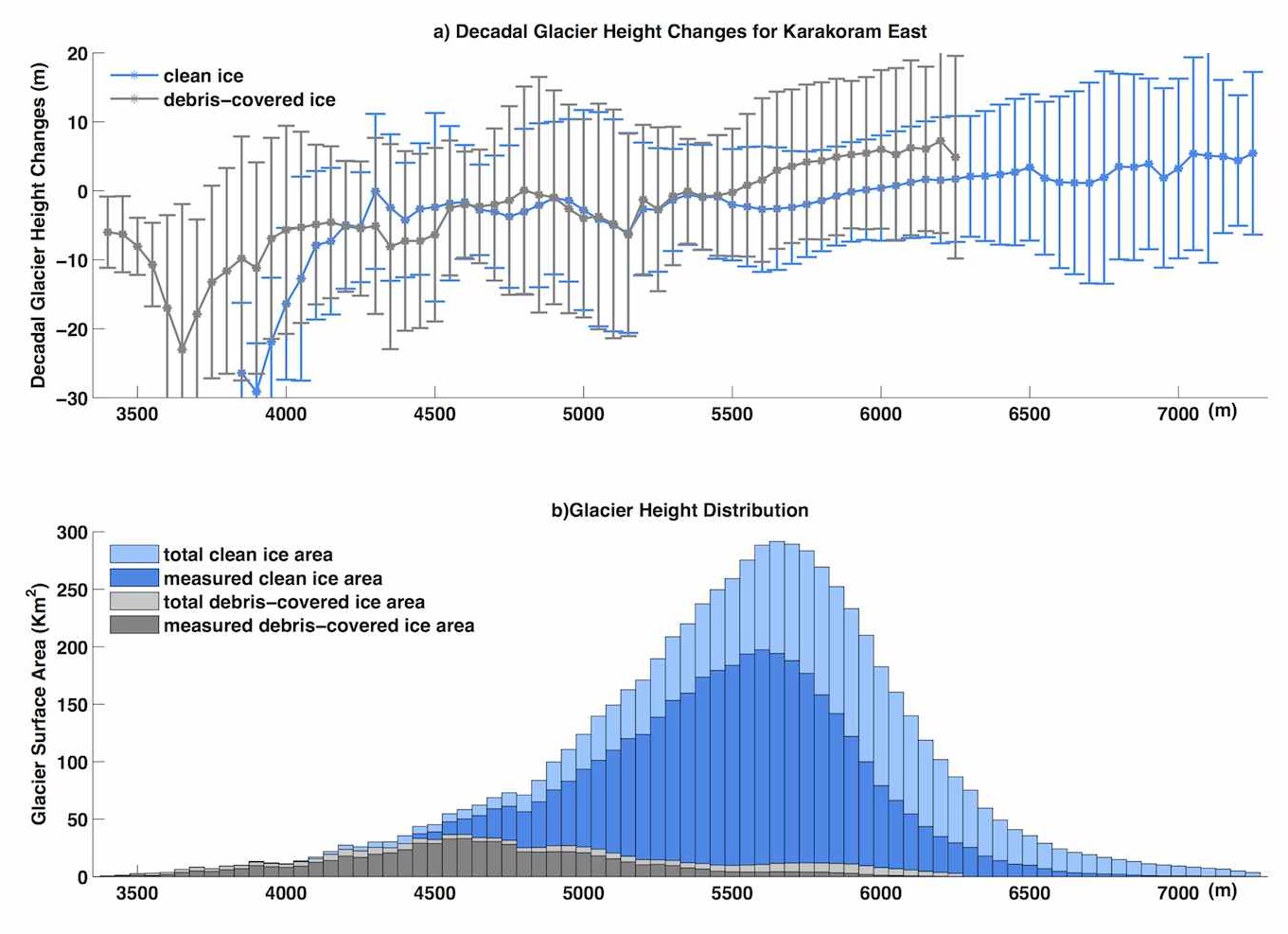


Supplementary Figure S21. Glacier height changes (a) and height distributions (b) at each elevation bin for Eastern Karakoram.


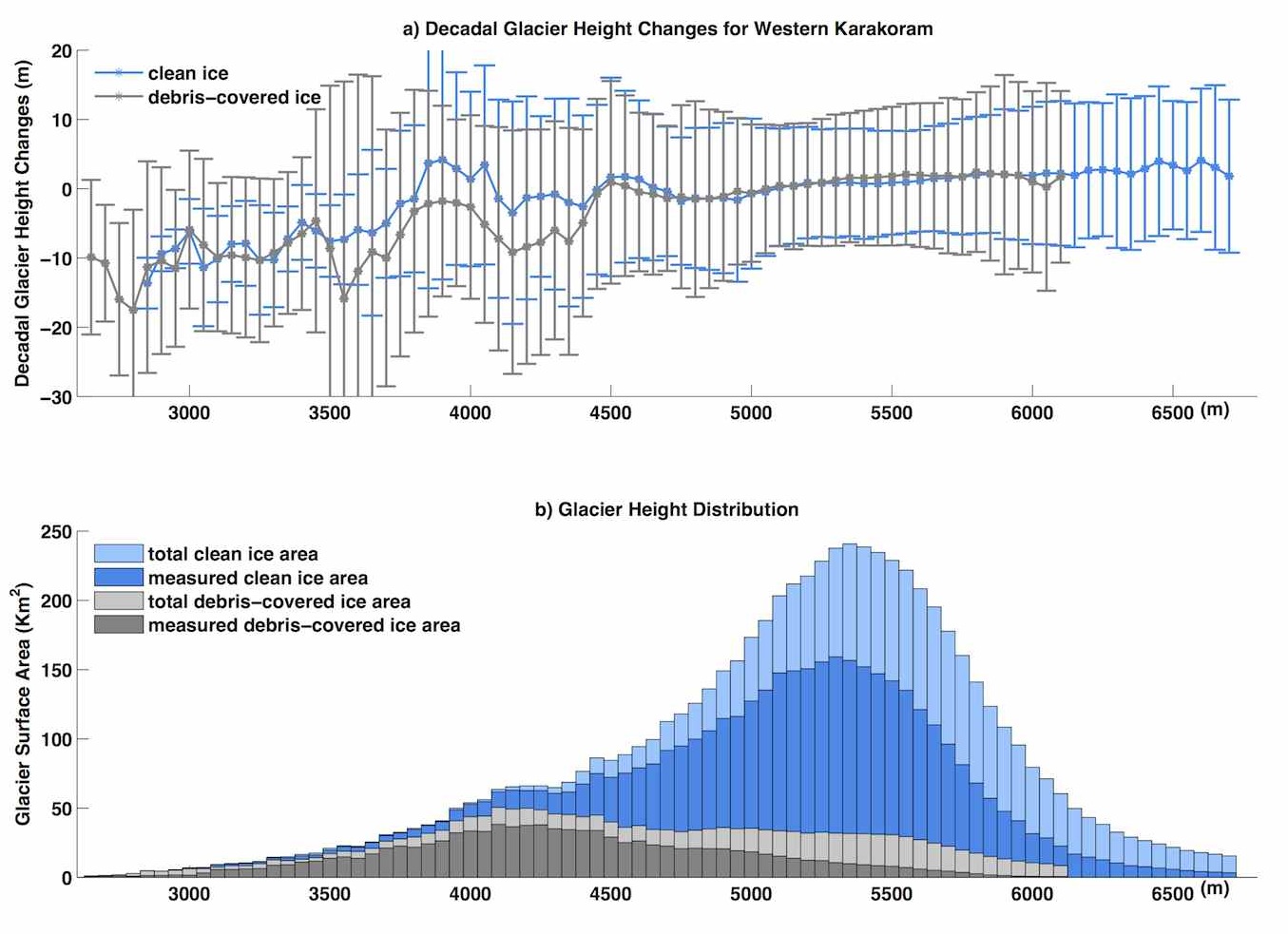


Supplementary Figure S22. Same as Figure S18 but for Western Karakoram.


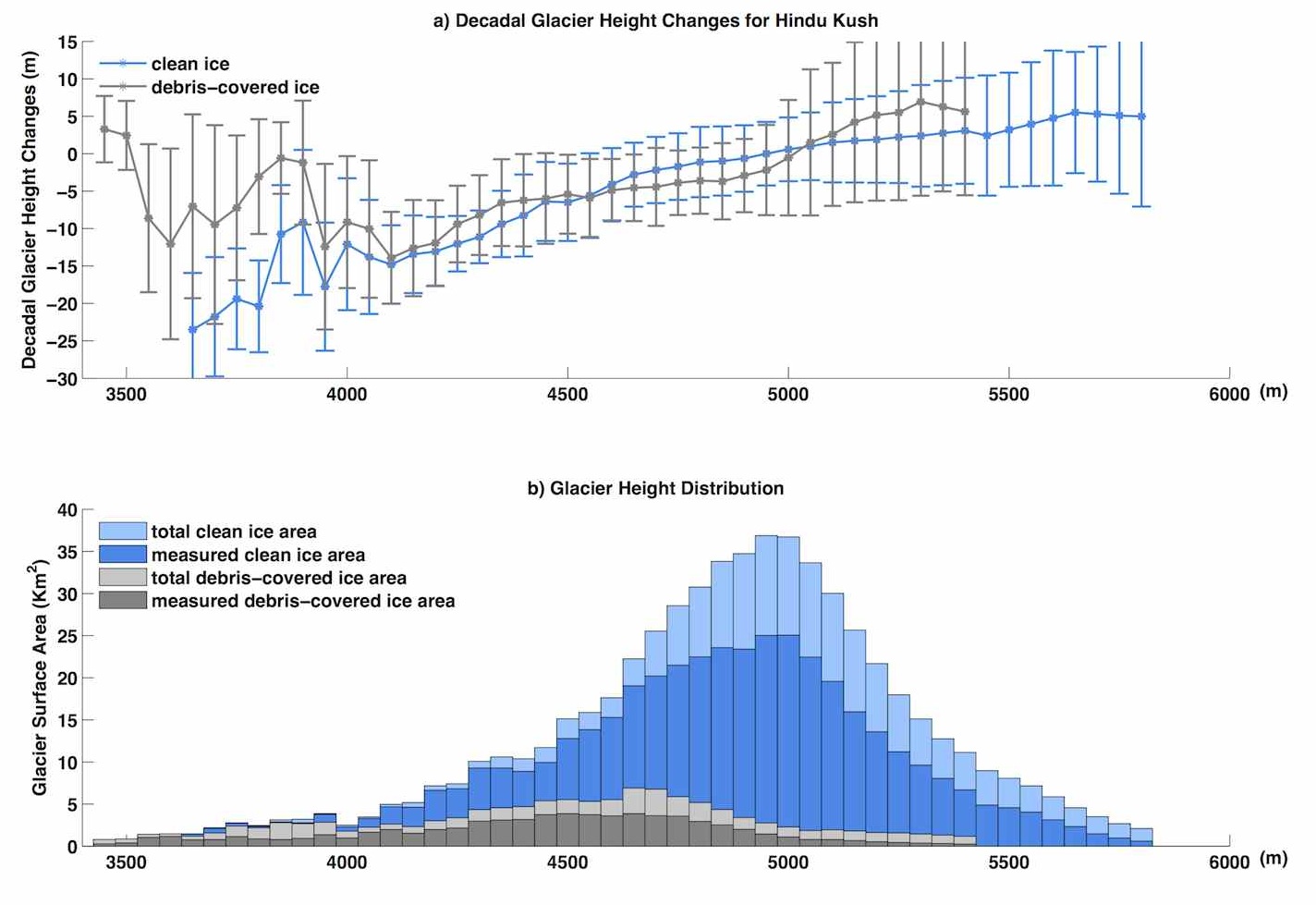


Supplementary Figure S23. Same as Figure S18 but for Hindu Kush.


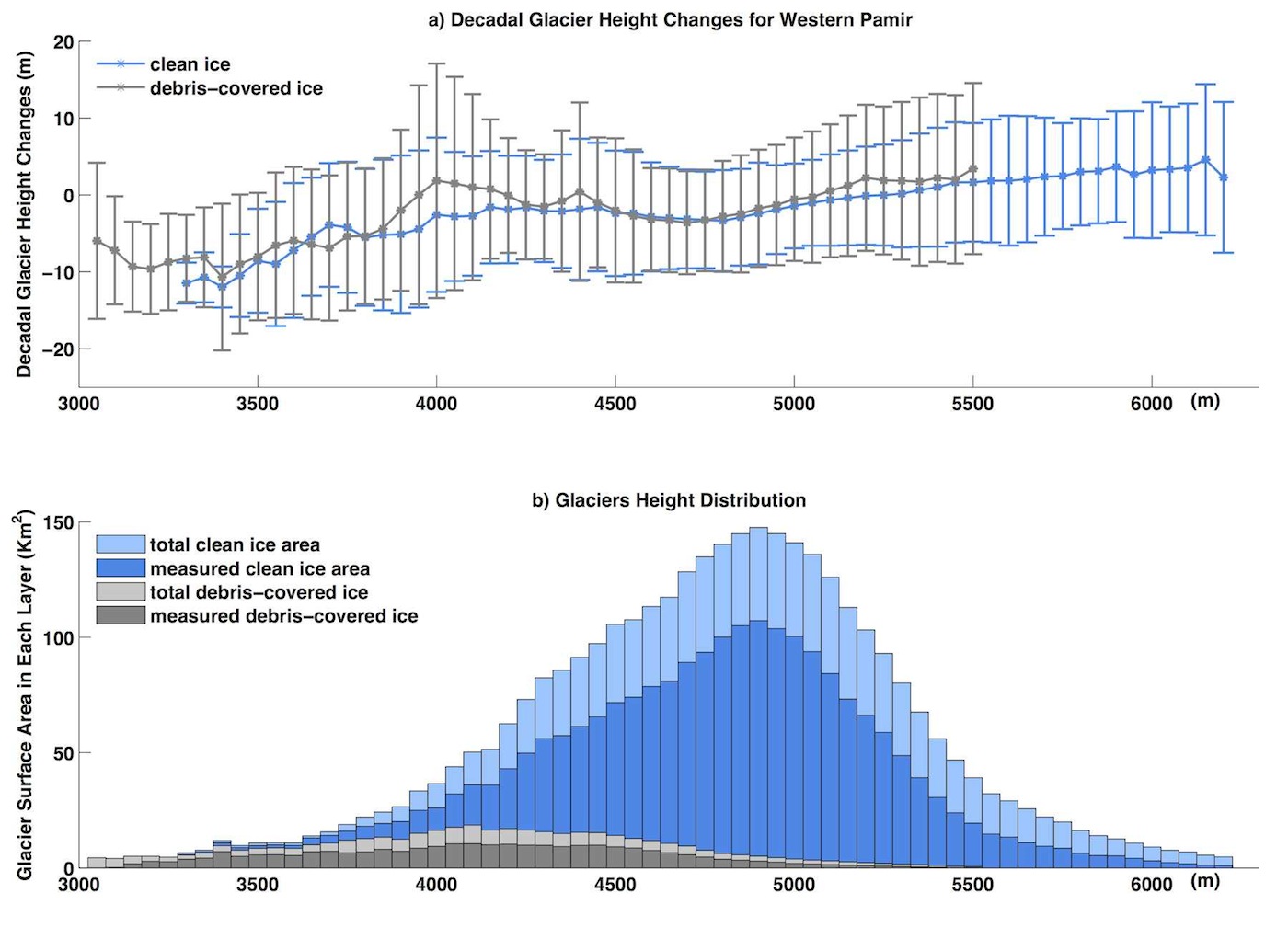


Supplementary Figure S24. Same as Figure S18 but for Western Pamir.


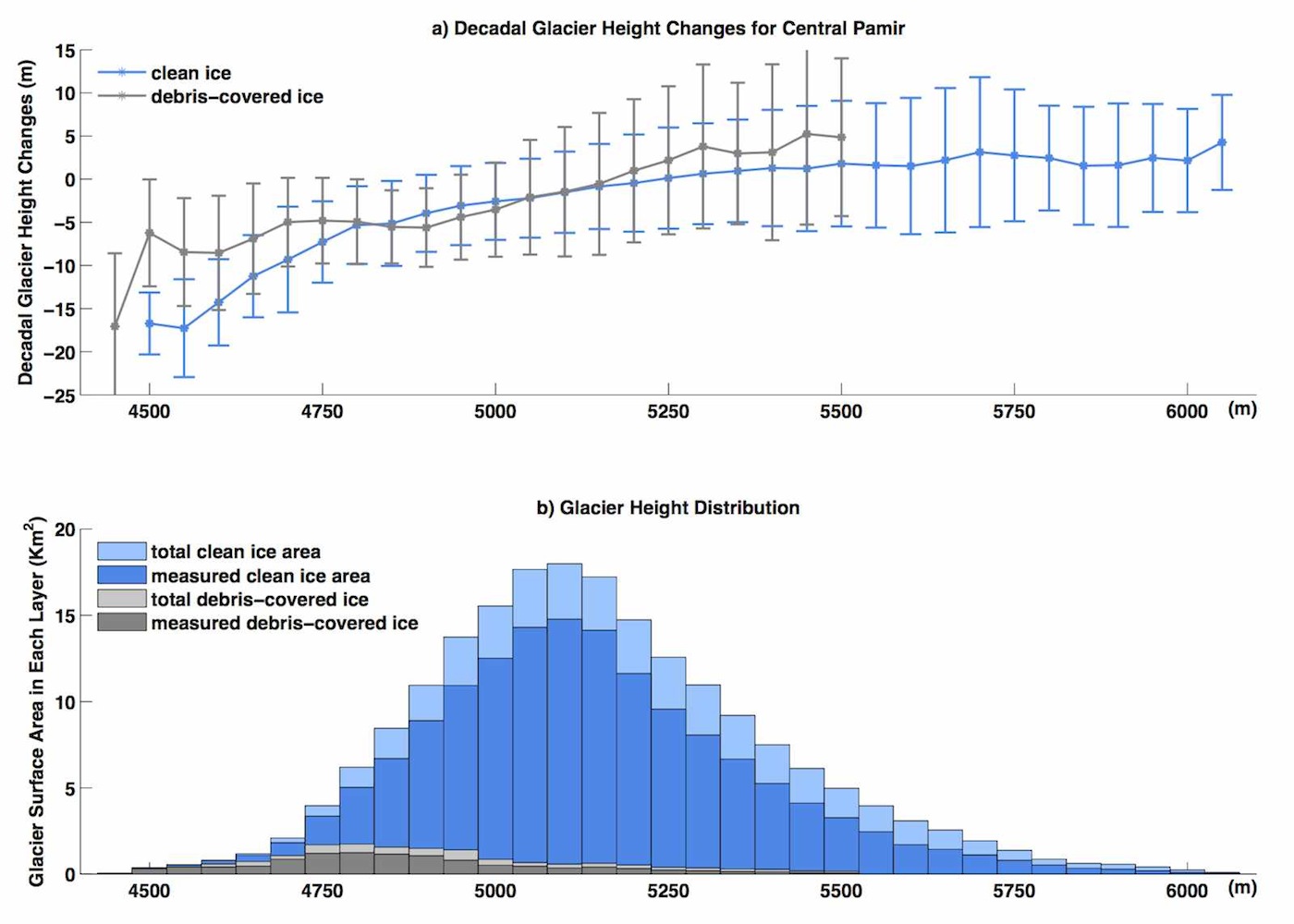


Supplementary Figure S25. Same as Figure S18 but for Central Pamir.


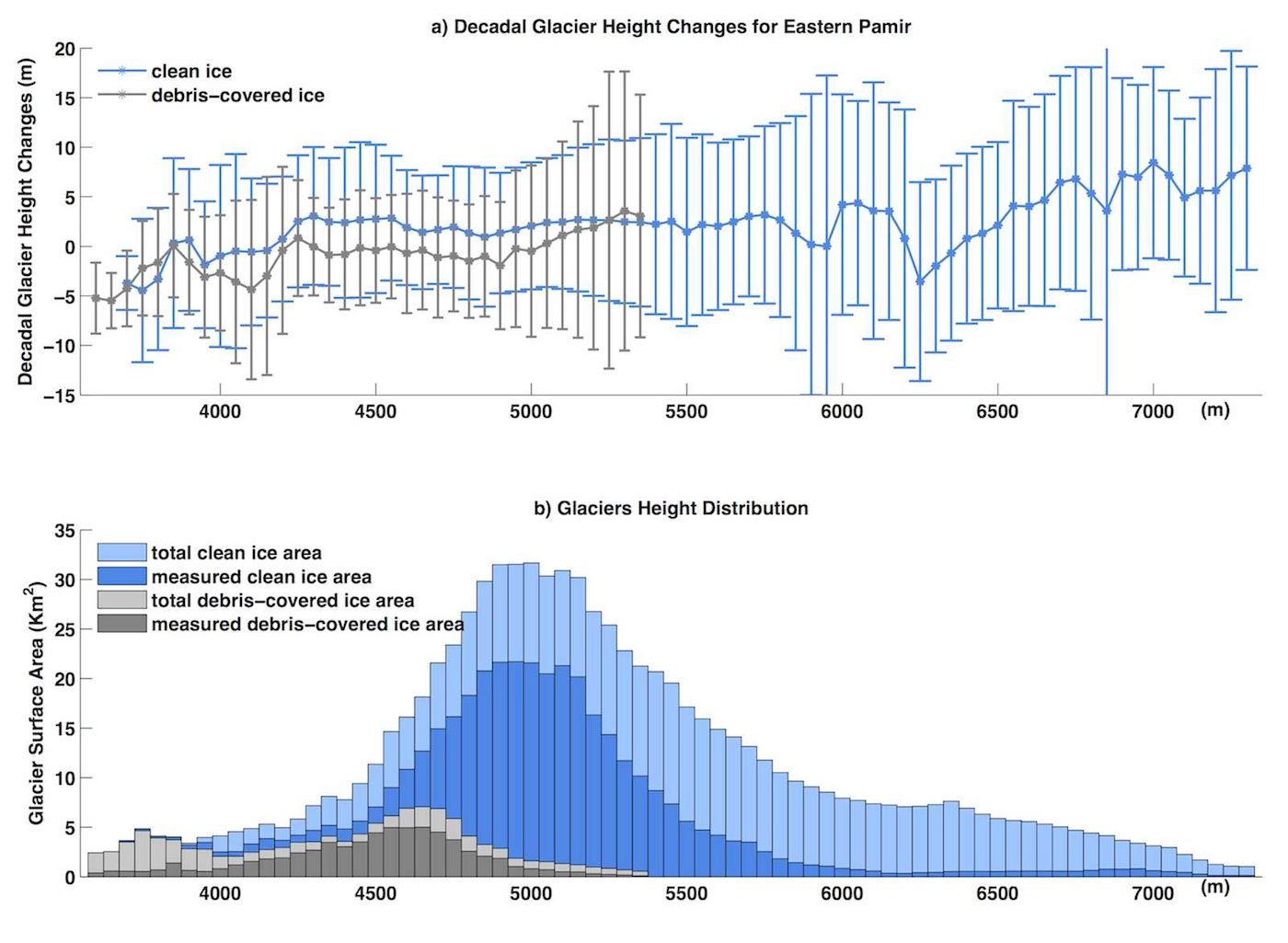


Supplementary Figure S26. Same as Figure S18 but for Eastern Pamir.


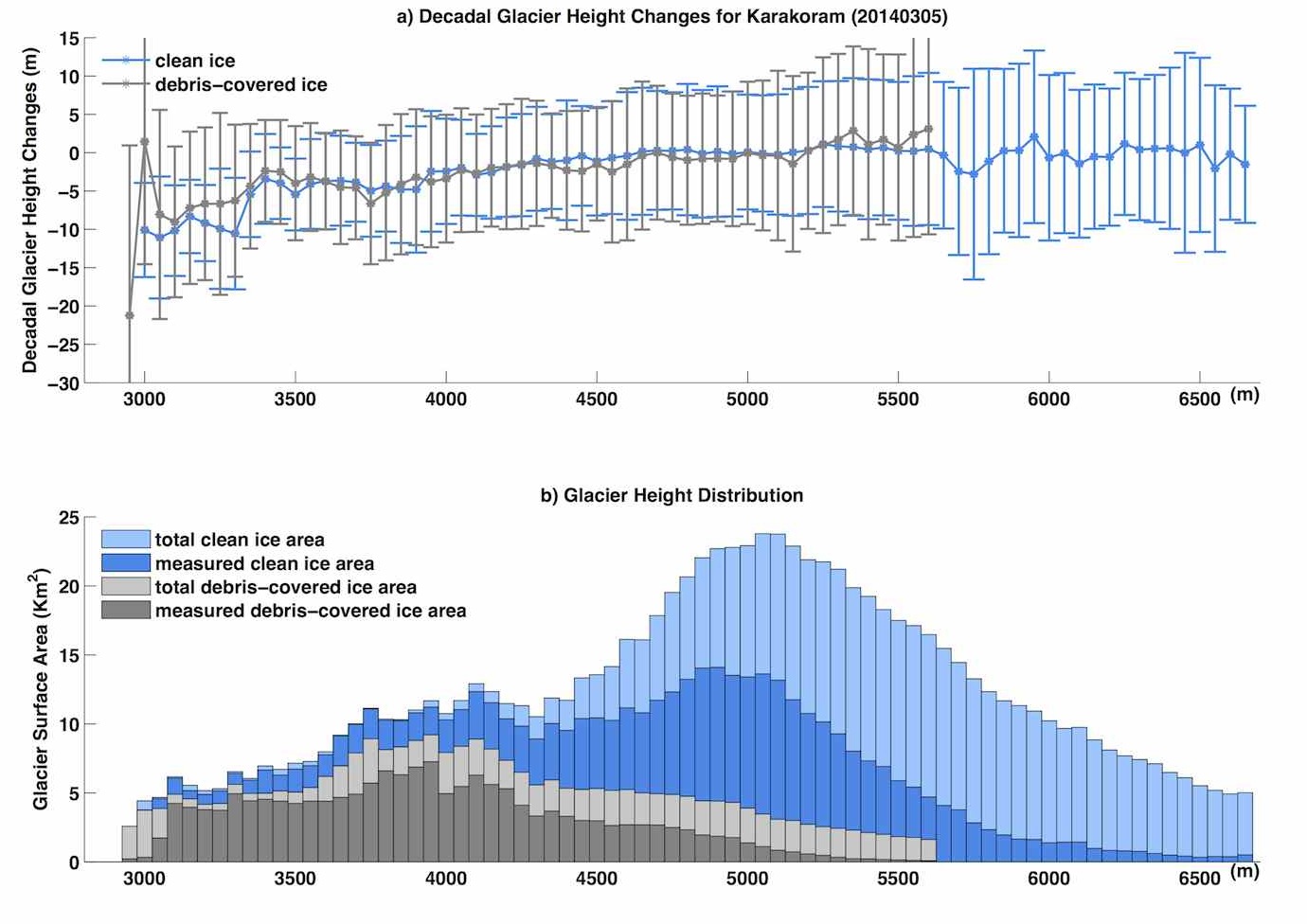


Supplementary Figure S27. Same as Figure S18 but for image coverage 2013-03-05 in Western Karakoram.


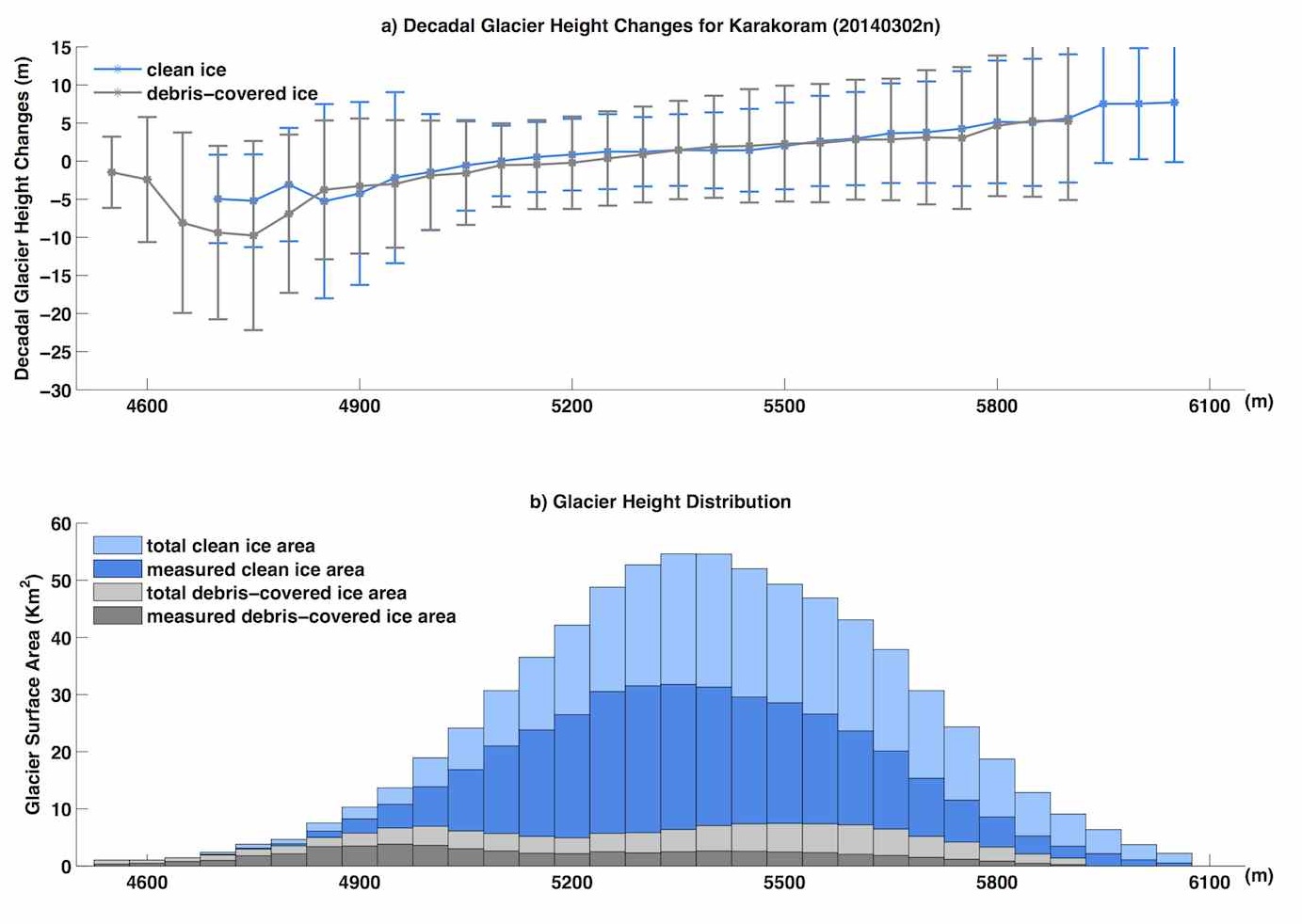


Supplementary Figure S28. Same as Figure S18 but for image coverage 2014-03-02n in Western Karakoram.


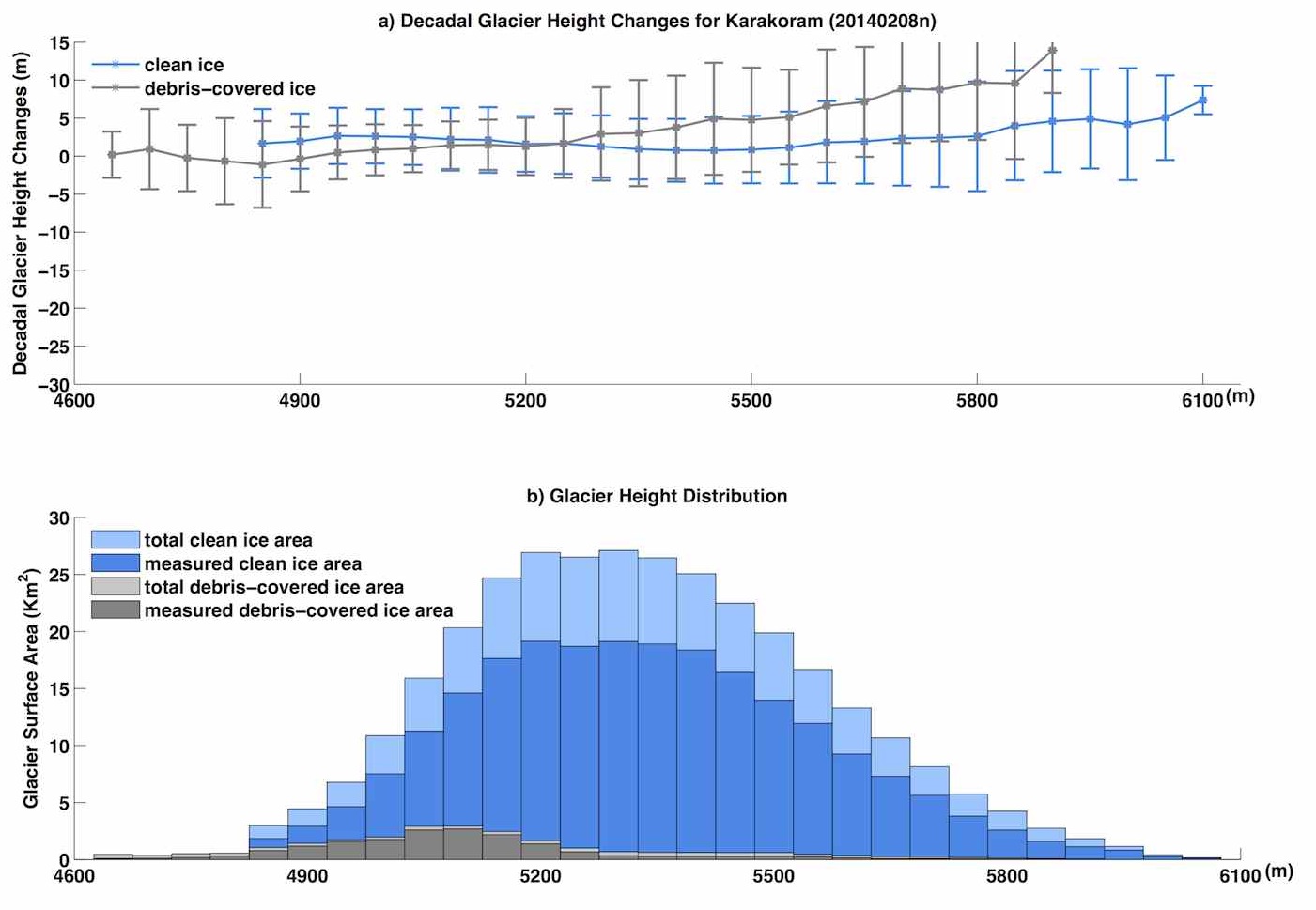


Supplementary Figure S29. Same as Figure S18 but for image coverage 2014-02-08n in Western Karakoram.


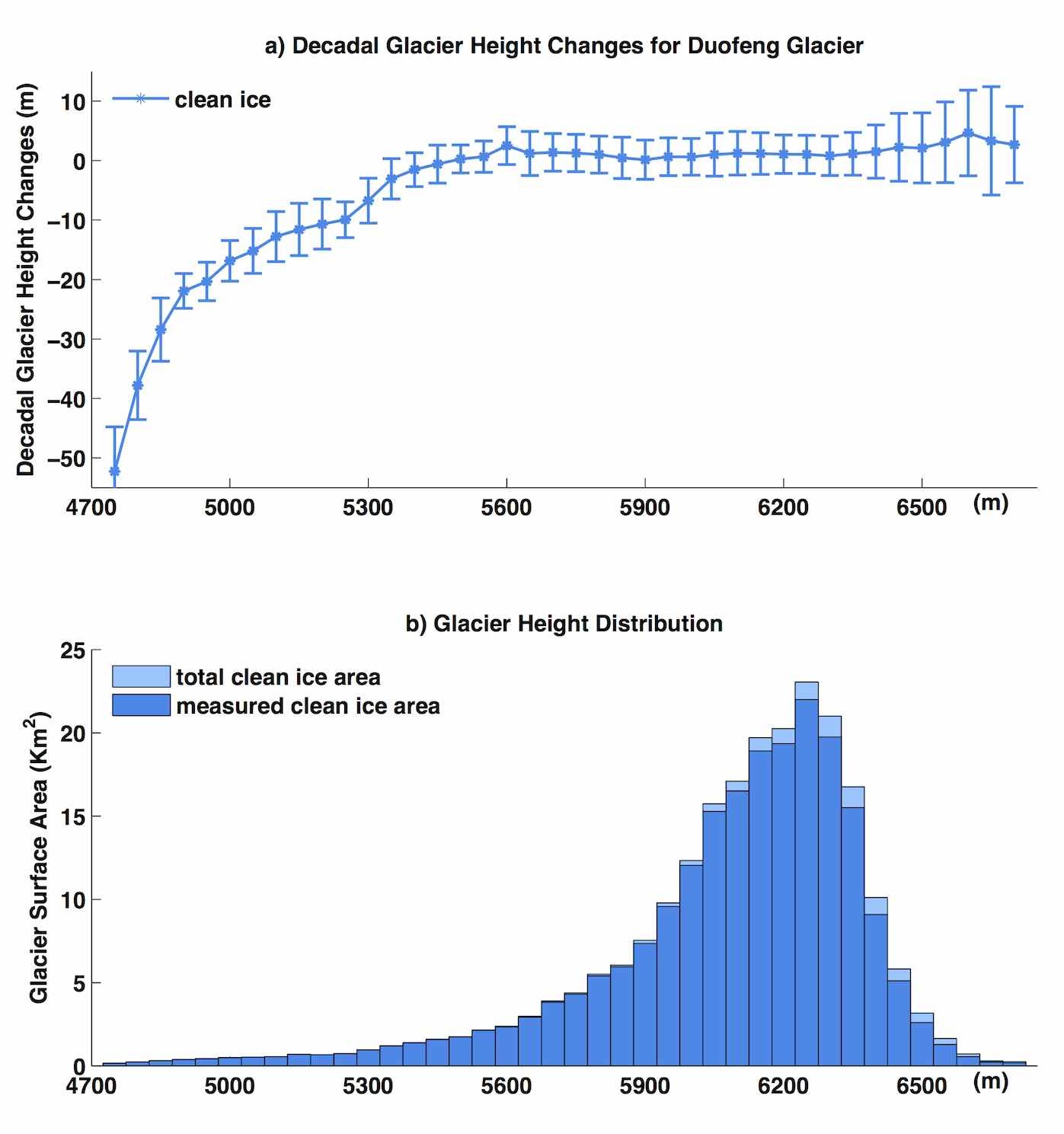


Supplementary Figure S30. Same as Figure S18 but for the Duofeng Glacier in West Kunlun.


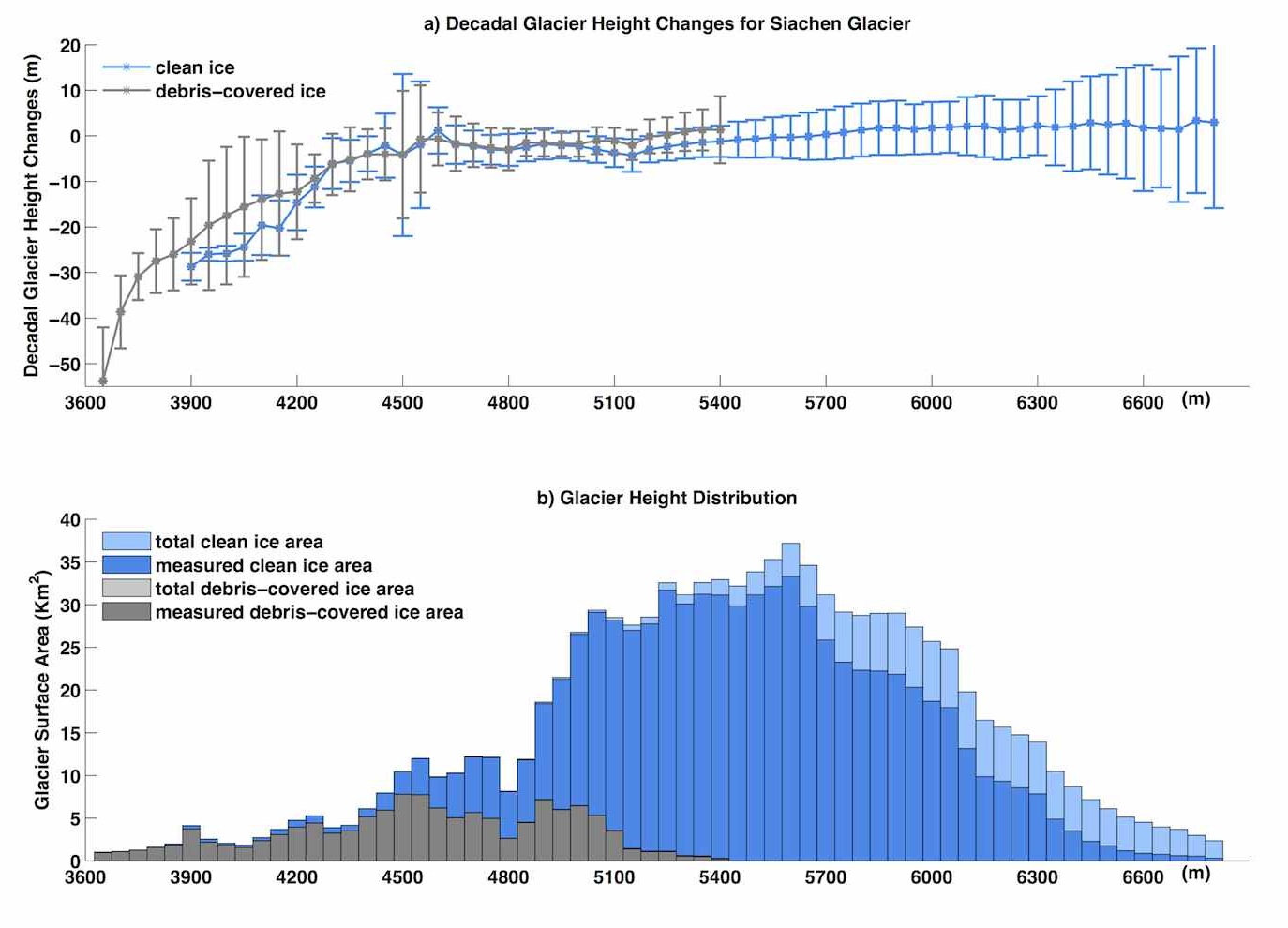


Supplementary Figure S31. Same as Figure S18 but for the Siachen Glacier in Eastern Karakoram.


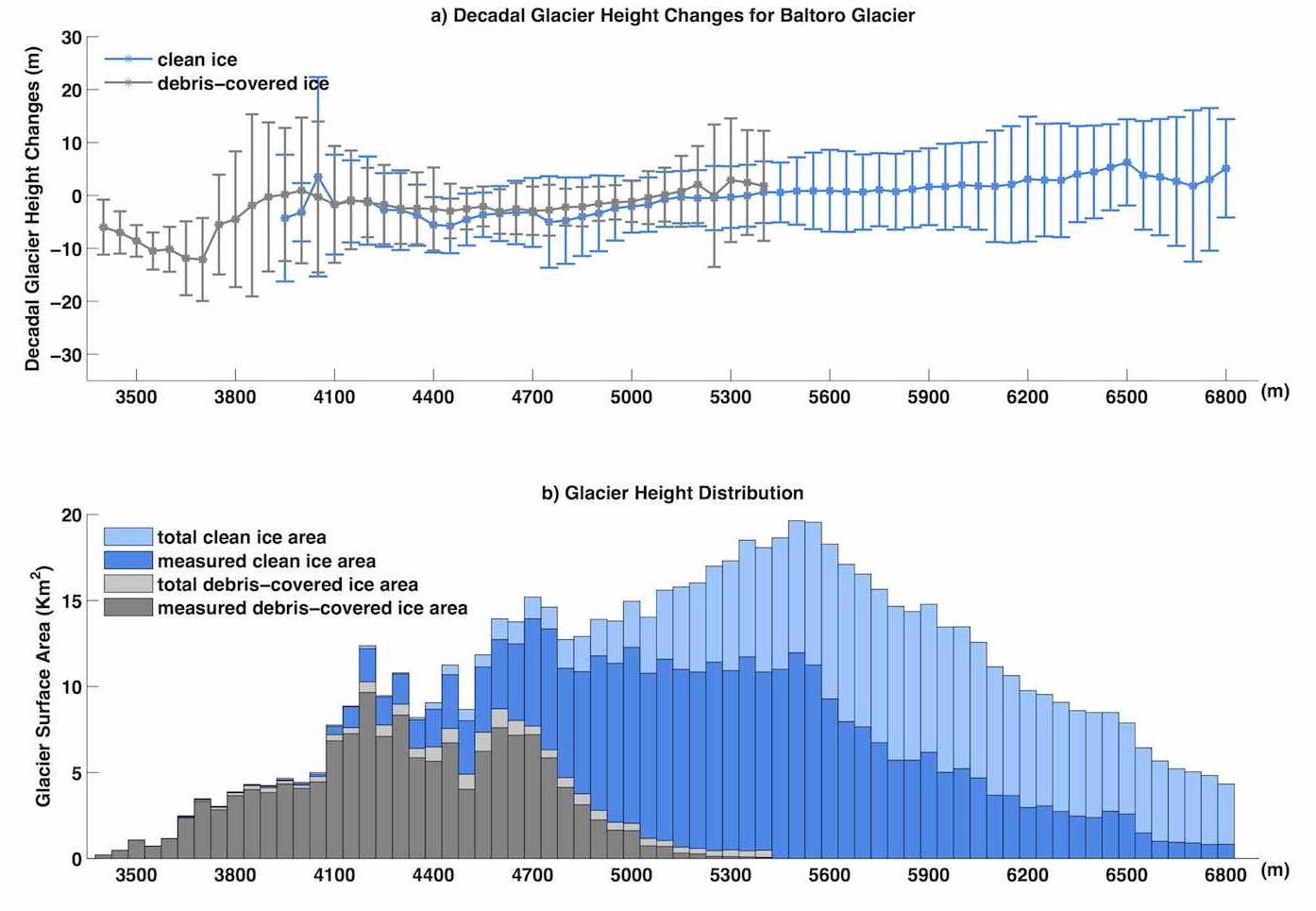


Supplementary Figure S32. Same as Figure S18 but for the Baltoro Glacier in Eastern Karakoram.


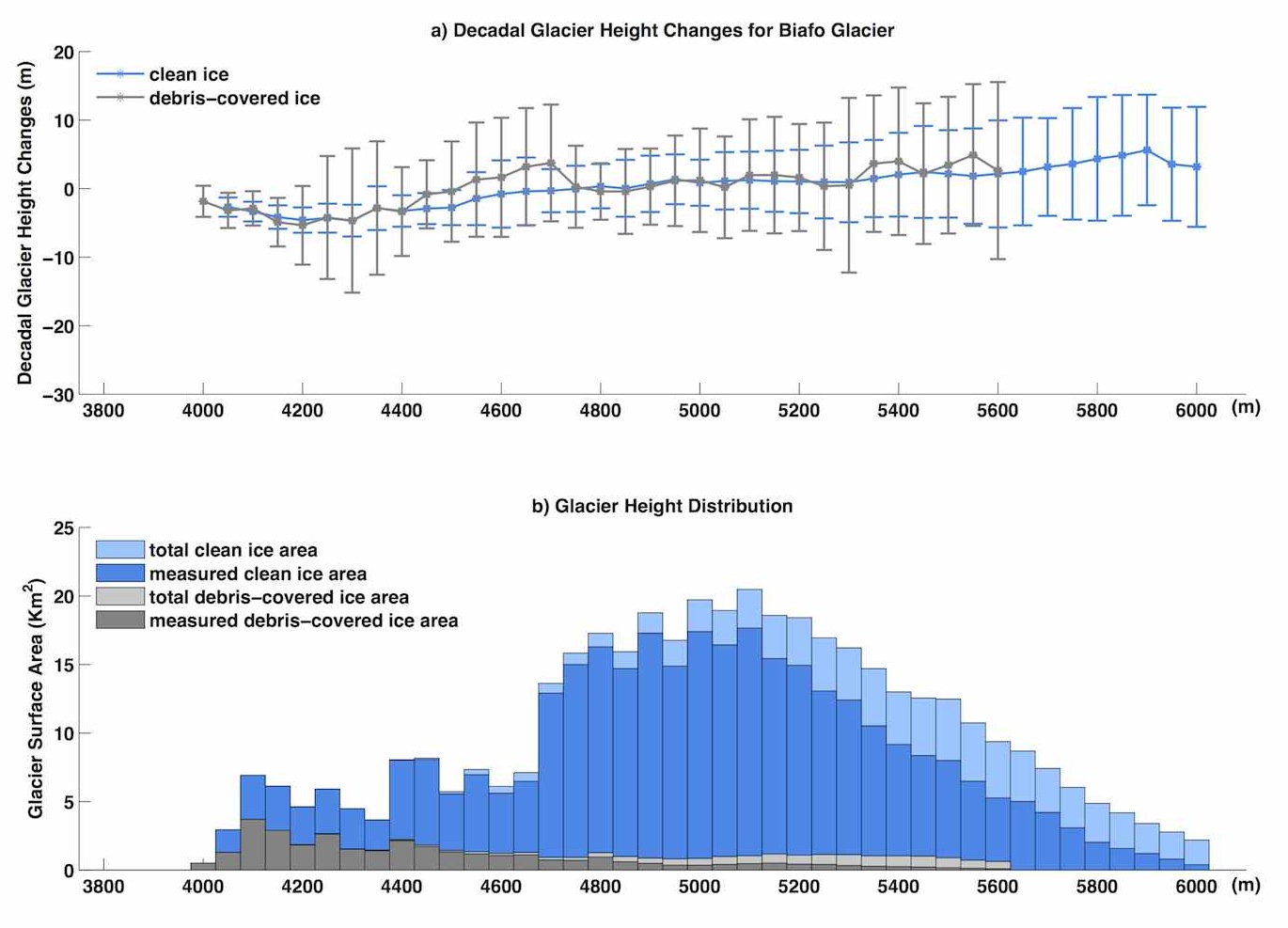


Supplementary Figure S33. Same as Figure S18 but for the Biafo Glacier in Western Karakoram.


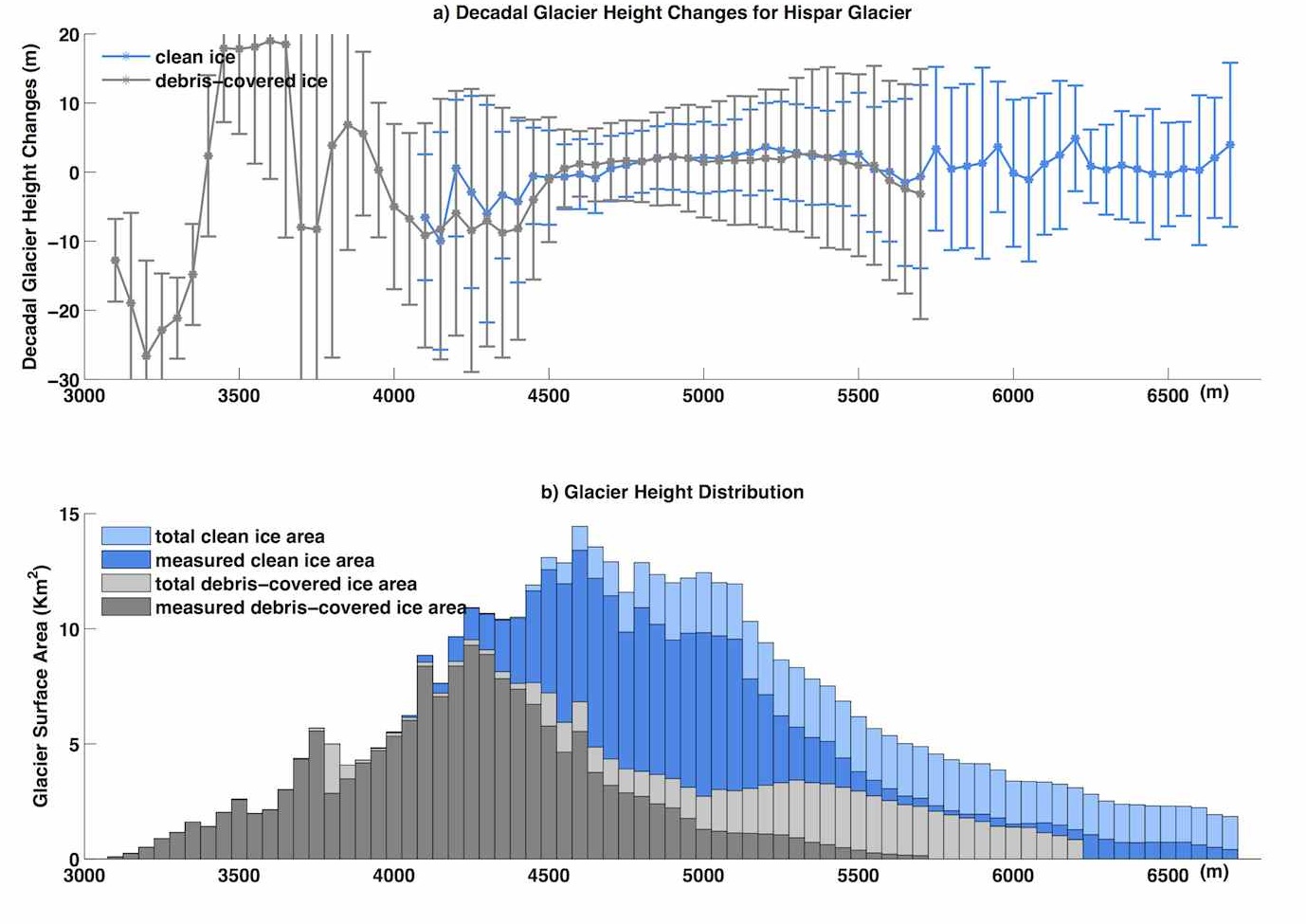


Supplementary Figure S34. Same as Figure S18 but for for the Hispar Glacier in Western Karakoram.


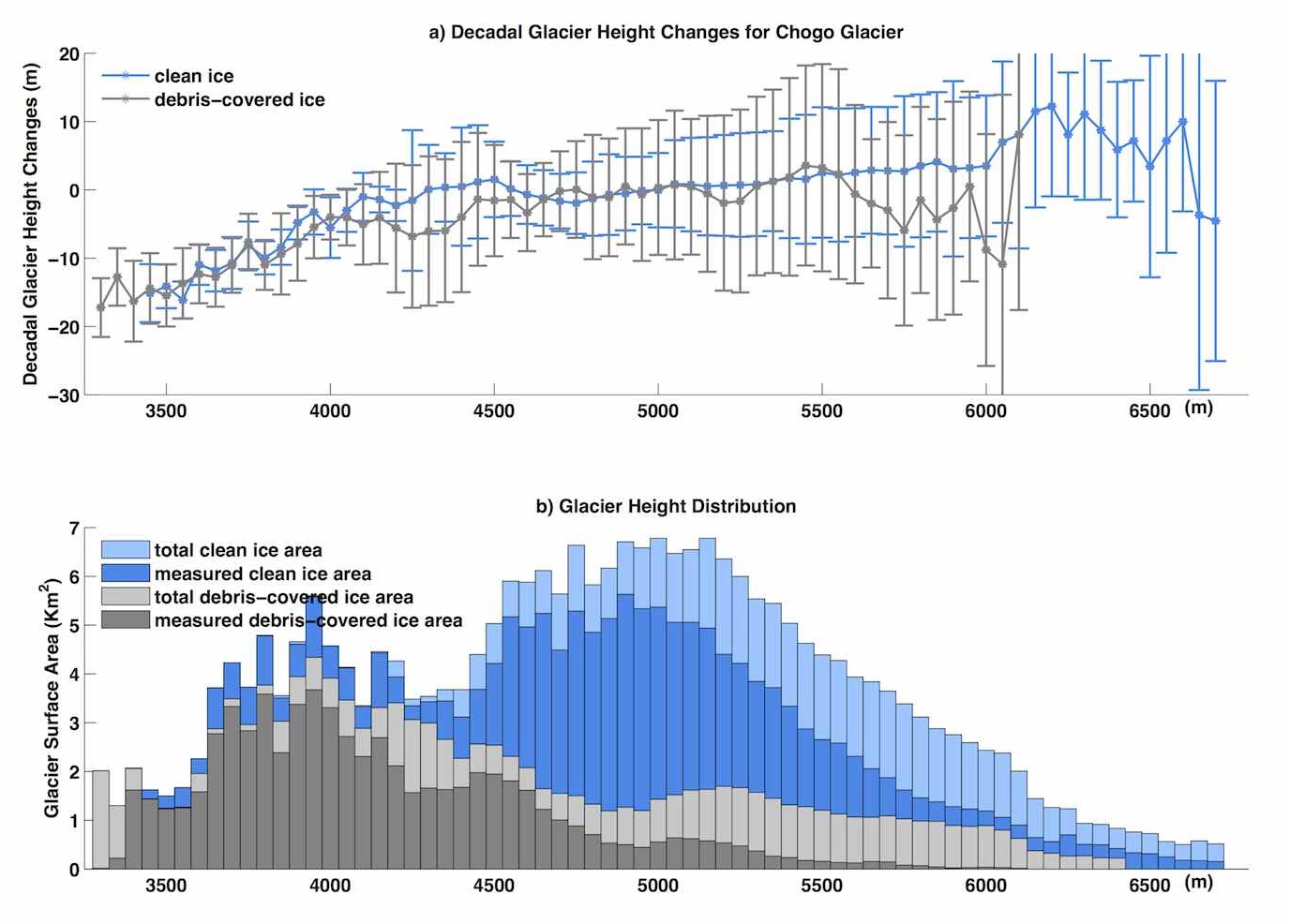


Supplementary Figure S35. Same as Figure S18 but for the Chogo Lungma Glacier in Western Karakoram.


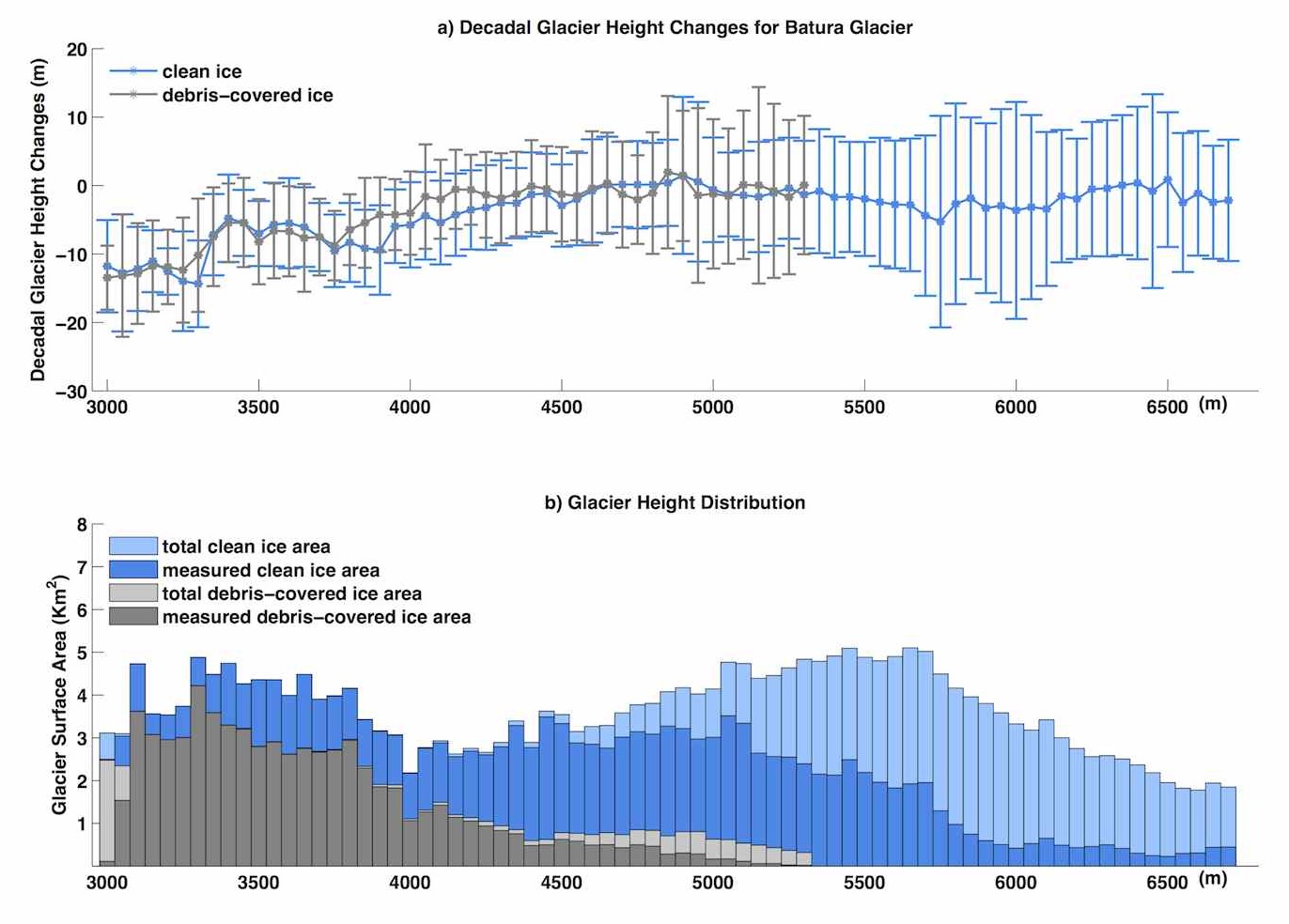


Supplementary Figure S36. Same as Figure S18 but for the Batura Glacier in Western Karakoram.


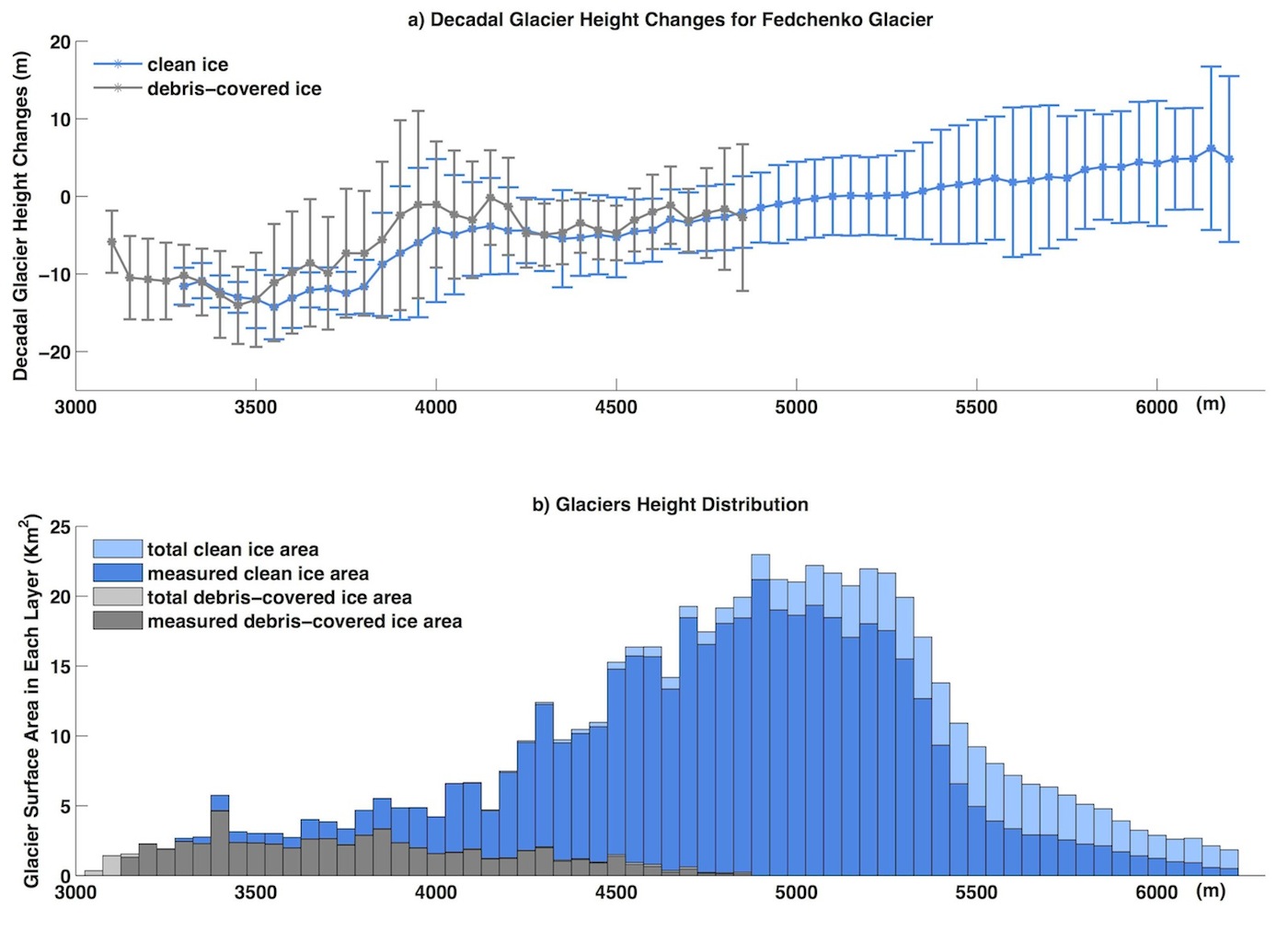


Supplementary Figure S37. Same as Figure S18 but for the Fedchenko Glacier in Western Pamir.


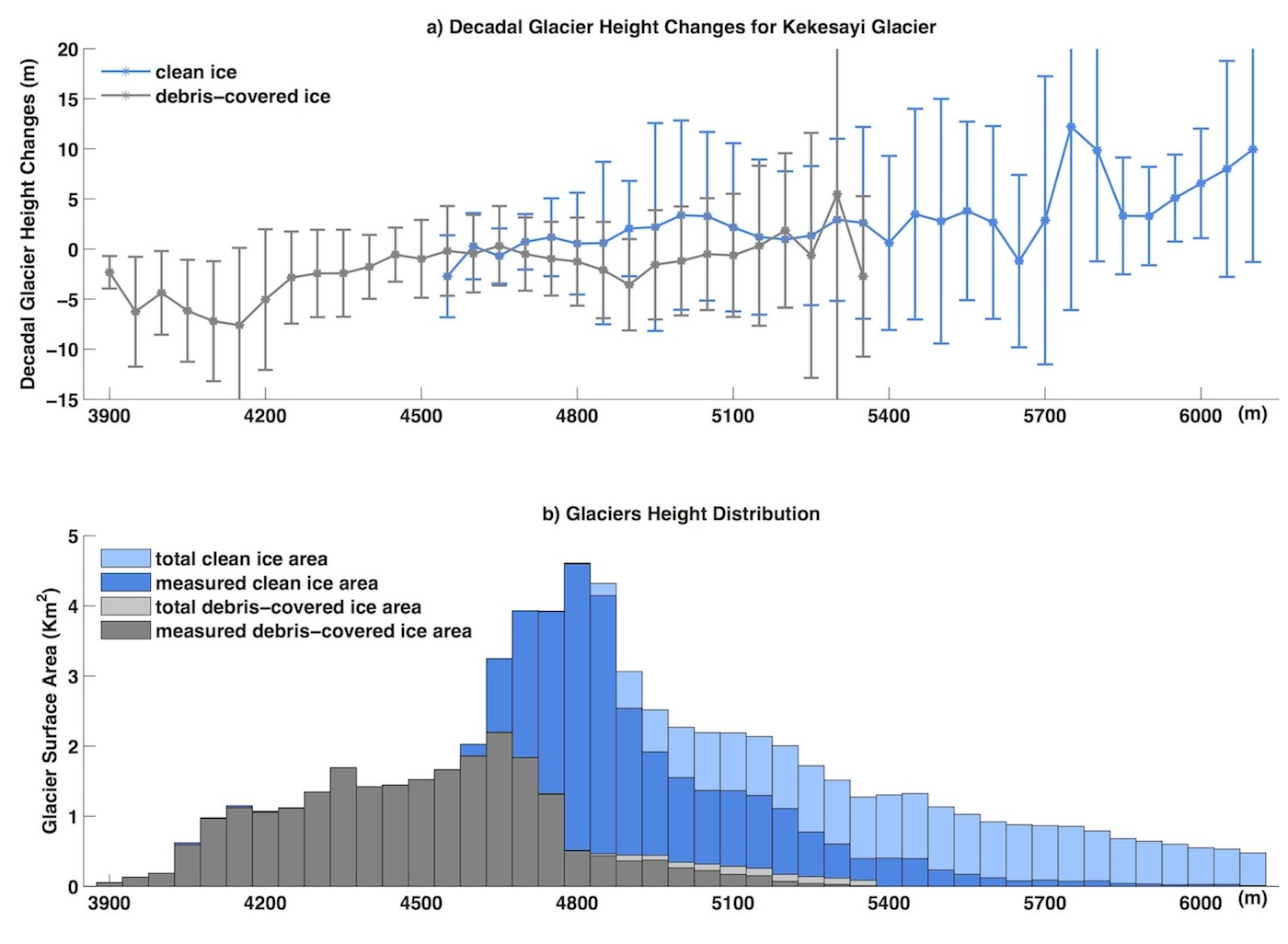


Supplementary Figure S38. Same as Figure S18 but for the Kekesayi Glacier in Eastern Pamir.

Supplementary Table S5. Glacier mass balance in unit of m w.e. yr^-1^ for the West Kunlun (WK) region. S stands for south, and belongs to the Inner Tibetan Plateau endorheic region, N stands for north, and belongs to the Upper Tarim Basin. W, C and E stand for west, central and east group.

| Region | Name | Sub-group | Length/Area | HMax/Min | Mass Balance |
| --- | --- | --- | --- | --- | --- |
| WK | Quanshui | S & W | 10.894/29.87 | 6352/5471 | 0.219 |
| WK | Bingshuihe | S & W | 15.839/85.53 | 5451/6482 | 0.186 |
| WK | Duota | S & W | 15.347/81.00 | 5461/6375 | 0.157 |
| WK | Gongxing | S & C | 18.997/108.18 | 5363/6657 | 0.005 |
| WK | Litian | S & C | 10.884/22.71 | 5388/6611 | -0.056 |
| WK | Zhongfeng | S & C | 24.140/237.46 | 5353/7122 | 0.116 |
| WK | Guozha | S & C | 12.490/32.67 | 5369/6486 | 0.097 |
| WK | Chongce | S & C | 29.008/166.08 | 5325/6825 | -0.048 |
| WK | S2 | S & C | 16.010/46.117 | 5460/6732 | 0.119 |
| WK | Guliya | S & E | 12.529/111.37 | 5479/6648 | 0.230 |
| WK | Alakesayi | N & E | 16.812/84.97 | 5365/6737 | 0.314 |
| WK | Xiezhi | N & E | 12.927/41.89 | 5456/6335 | 0.229 |
| WK | Yulong | N & E | 31.030/135.00 | 5117/6720 | 0.100 |
| WK | N7 | N & E | 17.750/24.89 | 4720/6587 | -0.345 |
| WK | N6 | N & C | 24.952/81.327 | 5032/6814 | -0.014 |
| WK | N5 | N & C | 18.742/61.91 | 4912/6671 | 0.076 |
| WK | Duofeng | N & C | 29.226/236.77 | 4728/6851 | 0.040 |
| WK | N3 | N & C | 18.956/90.26 | 5150/6647 | 0.163 |
| WK | Kunlun | N & C | 23.978/199.09 | 4892/6707 | 0.111 |
| WK | West Yulong | N & C | 22.219/120.51 | 5216/6593 | 0.139 |
| WK | N2 | N & W | 14.675/44.20 | 5289/6444 | 0.390 |
| WK | West Kunlun E | N & W | 16.776/51.478 | 5253/6421 | 0.389 |
| WK | West Kunlun W | N & W | 16.303/76.92 | 5280/6401 | 0.302 |

Supplementary Table S6. Glacier mass balance in unit of m w.e. yr^-1^ for the Eastern Karakoram (EKK) region. S stands for south, and belongs to the Upper Indus region; N stands for north, and belongs to the Upper Tarim Basin. C and E stand for central and east group. Glacier numbers are marked in figure S14.

| Region | No. | Name | Sub-group | Length/Area | HMax/Min | Mass Balance |
| --- | --- | --- | --- | --- | --- | --- |
| EKK | 1 | South Shukpa Kunchhang I | S & E | 20.586/87.68 | 7433/4739 | -0.271 |
| EKK | 2 | Sakang Lungpa | S & E | 13.393/39.35 | 4761/7254 | -0.135 |
| EKK | 3 | South Phukpo | S & E | 12.734/38.18 | 4903/7572 | -0.076 |
| EKK | 4 | Aktash | S & E | 14.622/25.23 | 4590/6960 | -0.446 |
| EKK | 5 | Kichik Kumdan | S & E | 21.572/66.24 | 4621/7441 | -0.219 |
| EKK | 6 | Chong Kumdan I | S & E | 19.275/44.56 | 4718/7427 | 0.149 |
| EKK | 7 | Chong Kumdan II | S & E | 17.392/62.32 | 4926/6959 | -0.463 |
| EKK | 8 | Mamostong | S & E | 14.728/63.47 | 4524/7245 | -0.159 |
| EKK | 9 | South Terong | S & E | 27.833/182.12 | 4356/7168 | -0.184 |
| EKK | 10 | Rimo | S & E | 46.263/439.62 | 4921/7290 | -0.194 |
| EKK | 11 | North Terong | S & C | 21.319/117.05 | 3980/7334 | 0.033 |
| EKK | 12 | Siachen | S & C | 76.288/1078.0 | 3596/7579 | -0.083 |
| EKK | 13 | Singkhu | N & C | 26.884/116.15 | 4517/5587 | -0.141 |
| EKK | 14 | Unnamed (K1) | N & C | 28.662/76.13 | 4419/7176 | -0.096 |
| EKK | 15 | Urbak | N & C | 27.078/81.14 | 4276/8022 | -0.035 |
| EKK | 16 | Gasherbrum | N & C | 25.843/108.12 | 4238/7945 | -0.073 |
| EKK | 17 | Unnamed (K2) | N & C | 15.415/41.62 | 4554/7474 | -0.084 |
| EKK | 18 | Unnamed (K3) | N & C | 21.866/93.858 | 4119/8535 | +0.068 |
| EKK | 19 | Baltoro | S & C | 63.448/809.11 | 3385/8569 | -0.010 |
| EKK | 20 | Aling | S & C | 18.141/56.26 | 3649/6432 | -0.224 |
| EKK | 21 | Ghandogoro | S & C | 21.564/66.13 | 3476/7050 | -0.067 |
| EKK | 22 | Bilafond | S & C | 21.762/132.55 | 3840/7112 | -0.031 |
| EKK | 23 | Gyong | S & C | 18.725/53.97 | 4164/6437 | -0.099 |

Supplementary Table S7. Glacier mass balance in unit of m w.e. yr^-1^ for the Western Karakoram (WKK) and the Hindu Kush (HK) regions. S stands for south, and belongs to the Upper Indus region; N stands for north, and belongs to the Tarim Basin. C and W stand for central and west group. All glaciers in the Hindu Kush region belong to the Upper Indus basin. Glacier numbers are marked in figure S15 & S16.

| Region | No. | Name | Sub-group | Length/Area | HMax/Min | Mass Balance |
| --- | --- | --- | --- | --- | --- | --- |
| WKK | 1 | Yanatsugat | N & C | 29.909/199.17 | 4123/7323 | 0.069 |
| WKK | 2 | Sugatyanatjilga | N & C | 41.178/393.02 | 3984/7179 | -0.077 |
| WKK | 3 | Unnamed (K4) | N & C | 15.573/57.58 | 4410/7010 | -0.092 |
| WKK | 4 | Panmah | S & C | 33.298/334.69 | 3516/7054 | -0.066 |
| WKK | 5 | Braldu | N & C | 35.951/175.98 | 3990/6461 | 0.070 |
| WKK | 6 | Unnamed (K5) | N & C | 14.381/36.526 | 4393/6276 | 0.134 |
| WKK | 7 | Virjerab | S & C | 40.178/167.43 | 3592/6368 | 0.089 |
| WKK | 8 | Khurdopin | S & C | 31.640/203.34 | 3314/7718 | 0.241 |
| WKK | 9 | Biafo | S & C | 67.807/559.81 | 3045/7148 | 0.044 |
| WKK | 10 | Yazgil | S & C | 31.403/133.75 | 3234/7709 | 0.246 |
| WKK | 11 | Hispar | S & C | 54.453/495.65 | 3110/7794 | -0.019 |
| WKK | 12 | Sosoun | S & C | 16.465/74.25 | 3680/6390 | -0.293 |
| WKK | 13 | Soiu | S & C | 15.601/59.80 | 3364/6300 | -0.081 |
| WKK | 14 | East Hucho Alcho | S & C | 14.411/38.39 | 3661/6195 | -0.049 |
| WKK | 15 | West Hucho Alcho | S & C | 12.077/43.00 | 3712/6056 | -0.095 |
| WKK | 16 | Chogo Lungma | S & C | 47.345/295.32 | 2769/7008 | -0.130 |
| WKK | 17 | Momhil | S & C | 30.252/73.26 | 2881/7506 | 0.178 |
| WKK | 18 | Gharesa | S & W | 21.541/78.00 | 3825/7523 | -0.164 |
| WKK | 19 | Barpu | S & W | 29.588/105.04 | 2810/7432 | -0.187 |
| WKK | 20 | Pasu | S & W | 23.651/62.19 | 2578/7487 | -0.134 |
| WKK | 21 | Batura | S & W | 59.662/311.65 | 2595/7725 | -0.263 |
| WKK | 22 | Kukki Jerab | S & W | 19.994/41.11 | 3597/6828 | -0.063 |
| WKK | 23 | Unnamed (K6) | S & W | 20.516/91.16 | 3005/7688 | -0.057 |
| WKK | 24 | Shuwart I Yaz | N & C | 9.364/23.74 | 4858/6067 | 0.058 |
| WKK | 25 | Unnamed (K7) | S & C | 12.041/41.08 | 4656/6170 | 0.106 |
| WKK | 26 | Unnamed (K8) | N & C | 8.695/28.47 | 4630/6028 | 0.073 |
| WKK | 27 | Unnamed (K9) | N & C | 9.770/17.17 | 4588/6169 | 0.076 |
| HK | HK1 | Perkhin | - | 12.035/11.21 | 3300/6047 | -0.057 |
| HK | HK2 | - | - | 10.213/13.36 | 3460/6029 | 0.205 |
| HK | HK3 | Chhateboi | - | 18.006/35.67 | 3552/6341 | -0.133 |
| HK | HK4 | - | - | 8.222/11.94 | 4352/5859 | -0.336 |
| HK | HK5 | - | - | 5.702/6.54 | 4647/4988 | -0.446 |
| HK | HK6 | - | - | 7.449/10.36 | 4202/5899 | -0.106 |
| HK | HK7 | - | - | 6.434/7.95 | 4175/5700 | -0.325 |
| HK | HK8 | - | - | 4.407/8.23 | 4366/5671 | -0.183 |
| HK | HK9 | - | - | 6.928/5.57 | 4348/5317 | -0.357 |
| HK | HK10 | - | - | 34.870/186.11 | 3689/6186 | -0.313 |
| HK | HK11 | - | - | 5.826/5.32 | 4250/5527 | -0.017 |

Supplementary Table S8. Glacier mass balance in unit of m w.e. yr^-1^ for the Pamir region. WPMR, CPMR and EPMR stand the Western, Central and Eastern Pamir. W means glaciers located west of theFedchenko Glacier and SE means southeast of the Fedchenko Glacier. Glacier Numbers are marked in figure S17 – S20.

| Region | No. | Name | Sub-group | Length/Area | HMax/Min | Mass Balance |
| --- | --- | --- | --- | --- | --- | --- |
| WPMR | 1 | Grumm-grzhimaylo | SE | 37.217/152.13 | 3615/6814 | -0.010 |
| WPMR | 2 | P1 | SE | 14.920/19.77 | 3862/6192 | -0.092 |
| WPMR | 3 | P2 | SE | 12.364/16.06 | 4097/6196 | -0.206 |
| WPMR | 4 | P3 | SE | 13.336/32.27 | 4048/5950 | -0.379 |
| WPMR | 5 | Fedchenko | - |  |  | -0.147 |
| WPMR | 6 | P4 | W | 25.305/129.38 | 2976/6712 | 0.034 |
| WPMR | 7 | P5 | W | 18.606/52.92 | 3750/6440 | 0.196 |
| WPMR | 8 | P6 | W | 21.734/81.38 | 2620/6537 | -0.225 |
| WPMR | 9 | P7 | W | 9.084/16.71 | 3600/5595 | -0.141 |
| WPMR | 10 | P8 | W | 10.586/16.23 | 3663/5291 | 0.091 |
| WPMR | 11 | P9 | W | 10.375/13.11 | 3528/5118 | -0.384 |
| WPMR | 12 | P10 | W | 10.694/19.39 | 3224/5147 | -0.010 |
| WPMR | 13 | P11 | W | 8.888/12.762 | 3264/5650 | 0.127 |
| WPMR | 14 | P12 | W | 12.520/26.50 | 3025/5812 | 0.006 |
| WPMR | 15 | P13 | W | 17.754/32.79 | 3279/5624 | 0.099 |
| WPMR | 16 | P14 | W | 16.174/38.352 | 3042/5880 | 0.012 |
| WPMR | 17 | P15 | W | 14.380/23.40 | 2924/5838 | 0.017 |
| WPMR | 18 | P16 | W | 12.844/27.24 | 3455/5945 | 0.089 |
| WPMR | 19 | P17 | W | 15.873/28.32 | 3408/6180 | 0.092 |
| WPMR | 20 | P18 | W | 16.547/37.50 | 3382/6205 | -0.154 |
| WPMR | 21 | P19 | SE | 11.710/28.59 | 3524/6348 | -0.081 |
| WPMR | 22 | P20 | SE | 11.424/28.46 | 3979/6374 | 0.063 |
| WPMR | 23 | P21 | SE | 10.567/21.785 | 3264/5551 | -0.304 |
| WPMR | 24 | P22 | SE | 9.219/20.055 | 4555/5738 | -0.420 |
| CPMR | 25 | P23 | SE | 7.271/7.59 | 4495/5879 | -0.245 |
| CPMR | 26 | P24 | SE | 8.142/9.65 | 4528/5826 | -0.141 |
| EPMR | EP1 | - | - | 15.185/44.79 | 4282/7156 | 0.225 |
| EPMR | EP2 | Qimugan | - | 18.619/86.63 | 3079/7566 | 0.212 |
| EPMR | EP3 | - | - | 11.619/22.95 | 4339/6180 | 0.016 |
| EPMR | EP4 | - | - | 13.162/26.47 | 3843/6411 | 0.258 |
| EPMR | EP5 | - | - | 8.850/12.763 | 4269/6083 | 0.206 |
| EPMR | EP6 | Kekesayi | - | 22.062/77.73 | 3910/7520 | 0.055 |
| EPMR | EP7 | Kuokuosele | - | 10.243/19.89 | 4454/6811 | 0.176 |
| EPMR | EP8 | - | - | 4.608/6.54 | 4809/5552 | 0.246 |
| EPMR | EP9 | - | - | 6.910/9.90 | 4705/5474 | 0.155 |
| EPMR | EP10 | - | - | 5.934/4.24 | 4241/5760 | 0.145 |
| EPMR | EP11 | - | - | 6.368/8.44 | 4297/5808 | 0.186 |

Reference

1. Gardelle J, Berthier E, Arnaud Y. Slight mass gain of Karakoram glaciers in the early twenty-first century. *Nature geoscience*. **5**, 322-325 (2012).

2. Gardelle, J, Berthier, E, Arnaud, Y, Kääb, A. Region-wide glacier mass balances over the Pamir-Karakoram-Himalaya during 1999–2011. *The Cryosphere*. **7**, 1263–1286 (2013).

3. Kääb A, Berthier E, Nuth C, et al. Contrasting patterns of early twenty-first-century glacier mass change in the Himalayas. *Nature*. **488**, 495-498 (2012).

4. Kääb A, Treichler D, Nuth C, et al. Brief Communication: Contending estimates of 2003–2008 glacier mass balance over the Pamir–Karakoram–Himalaya. *The Cryosphere*. **9**, 557-564 (2015).

5. Gardner A S, Moholdt G, Cogley J G, et al. A reconciled estimate of glacier contributions to sea level rise: 2003 to 2009. *Science*. **340**, 852-857 (2013).

6. Arendt, A.et al, Randolph Glacier Inventory – A Dataset of Global Glacier Outlines: Version 5.0. Global Land Ice Measurements from Space, Boulder Colorado, USA. (Digital Media, 2015).

7. Jaber W A, Floricioiu D, Rott H, et al. Dynamics of fast glaciers in the Patagonia Icefields derived from TerraSAR-X and TanDEM-X data. *Geoscience and Remote Sensing Symposium (IGARSS)*: 3226-3229 (2012).

8. Neckel N, Braun A, Kropáček J, et al. Recent mass balance of the Purogangri Ice Cap, central Tibetan Plateau, by means of differential X-band SAR interferometry. *The Cryosphere*, **7**, 1623-1633 (2013).

9. Rignot E, Echelmeyer K, Krabill W. Penetration depth of interferometric synthetic-aperture radar signals in snow and ice. *Geophysical Research Letters*. **28**, 3501-3504 (2001).

10. Moreira A, Prats-Iraola P, Younis M, et al. A tutorial on synthetic aperture radar. *IEEE Geoscience and Remote Sensing Magazine*. **1**, 6-43 (2013).

11. Hooper A, Bekaert D, Spaans K, et al. Recent advances in SAR interferometry time series analysis for measuring crustal deformation. *Tectonophysics*. **514**: 1-13 (2012).

12. Nuth C, Kääb A. Co-registration and bias corrections of satellite elevation data sets for quantifying glacier thickness change. *The Cryosphere*. **5**, 271-290, (2011).

13. Paul F, Bolch T, Kääb A, et al. The glaciers climate change initiative: Methods for creating glacier area, elevation change and velocity products. *Remote Sensing of Environment*. **162**. 408-426 (2015).

14. Hoffmann J, Walter D. How complementary are SRTM-X and-C band digital elevation models?. *Photogrammetric Engineering & Remote Sensing.* **72,** 261-268 (2006).

15. Yasuda T, Furuya M. Short-term glacier velocity changes at west kunlun shan, northwest tibet, detected by synthetic aperture radar data. *Remote Sensing of Environment*. **128**, 87-106 (2013).
